# Outpatient or Inpatient Setting for Cervical Ripening Before Induction of Labour: An Individual Participant Data Meta‐Analysis

**DOI:** 10.1111/1471-0528.18253

**Published:** 2025-06-11

**Authors:** Malitha Patabendige, Fei Chan, Michelle R. Wise, John M. D. Thompson, Michael Beckmann, Antonio F. Saad, George R. Saade, Akila Subramaniam, Alan Tita, Catarina Policiano, Nuno Clode, Amanda Henry, Henna Haavisto, Kirsi Rinne, Vicky Chen, Penelope Sheehan, Katherine Kohari, Hillary Hosier, Rebecca Pierce‐Williams, Vincenzo Berghella, Daniel L. Rolnik, Ben W. Mol, Wentao Li

**Affiliations:** ^1^ Department of Obstetrics and Gynaecology Monash University Melbourne Australia; ^2^ Monash Women's Monash Health Melbourne Australia; ^3^ Department of Obstetrics, Gynaecology and Reproductive Sciences, Faculty of Medical and Health Sciences The University of Auckland Auckland New Zealand; ^4^ Department of Paediatrics: Child and Youth Health, Faculty of Medical and Health Sciences The University of Auckland Auckland New Zealand; ^5^ Mater Health and Mater Research The University of Queensland Queensland Australia; ^6^ Perinatal Research Unit Inova Health Fairfax Falls Church Virginia USA; ^7^ Division of Maternal‐Fetal Medicine University of Texas Medical Branch Galveston Texas USA; ^8^ Department of Obstetrics and Gynecology Eastern Virginia Medical School Norfolk Virginia USA; ^9^ Center for Women's Reproductive Health and Department of Obstetrics and Gynecology University of Alabama at Birmingham Birmingham Alabama USA; ^10^ Department of Obstetrics, Gynecology and Reproductive Medicine Hospital de Santa Maria, Unidade Local de Saúde de Santa Maria Lisboa Portugal; ^11^ Universitary Clinic of Obstetrics and Gynecology Medical School, University of Lisbon Lisbon Portugal; ^12^ Discipline of Women's Health School of Clinical Medicine, UNSW Medicine & Health Sydney Australia; ^13^ Department of Obstetrics and Gynecology Turku University Hospital and University of Turku Turku Finland; ^14^ Department of Obstetrics and Gynaecology Eastern Health Melbourne Victoria Australia; ^15^ Department of Obstetrics, Gynecology and Reproductive Sciences Yale School of Medicine and Yale New Haven Hospital New Haven Connecticut USA; ^16^ Department of Obstetrics and Gynaecology Emory University Hospital Atlanta Georgia USA; ^17^ Department of Obstetrics and Gynecology Sidney Kimmel Medical College of Thomas Jefferson University Philadelphia USA; ^18^ National Perinatal Epidemiology and Statistics Unit (NPESU), Centre for Big Data Research in Health, and School of Clinical Medicine, Faculty of Medicine University of New South Wales Sydney Australia

**Keywords:** ambulatory, cervical ripening, home, individual participant data, induction of labour, IPD, meta‐analysis, outpatient, randomised trials

## Abstract

**Background:**

The optimal methods and settings for induction of labour (IOL) in terms of effectiveness, safety, and women's experience are still not elucidated.

**Objective:**

To compare the effectiveness and safety of outpatient versus inpatient cervical ripening settings for IOL.

**Search Strategy:**

MEDLINE, Embase, Emcare, CINAHL Plus, Scopus, Cochrane Library, WHO ICTRP and clinicaltrials.gov from inception to July 2024.

**Selection Criteria:**

Randomised controlled trials, viable singleton gestation, no language restrictions, all the published and unpublished data.

**Data Collection and Analysis:**

An individual participant data meta‐analysis.

**Main Results:**

Eleven out of 18 (61.1%) eligible RCTs shared IPD, totalling 2593 pregnant individuals undergoing IOL (62.2% of all participants in the published RCTs). Among the shared RCTs, four used balloon catheters alone in both groups. Three RCTs compared outpatient balloon catheter with inpatient balloon catheter plus oxytocin. Another three RCTs compared outpatient balloon catheter to inpatient vaginal dinoprostone. One RCT used Dilapan‐S in both groups. No trials evaluating outpatient use of vaginal prostaglandins were identified. Vaginal birth (11 RCTs, 2584 women, 67.8% vs. 70.2%, aOR 0.95, 95% CI 0.70; 1.30), composite perinatal outcome (9 RCTs, 2525 women, 11.1% vs. 11.7%, aOR 0.93, 95% CI 0.75; 1.16) and composite maternal (10 RCTs, 2480 women, 14.3% vs. 15.4%, aOR 0.89, 95% CI 0.65; 1.20) outcome did not differ between outpatient and inpatient groups. The outpatient group had a lower risk of acidosis, more epidural analgesia, and more oxytocin. There were no perinatal deaths in either group.

**Conclusions:**

Overall effectiveness, perinatal and maternal safety are comparable between outpatient setting cervical ripening with a mechanical method and inpatient with any method.

**Trial Registration:**

PROSPERO: CRD42022313183

## 1 Introduction

Induction of labour (IOL) is one of the most common interventions for childbirth worldwide. It is performed when medical intervention is deemed necessary compared to expectant management for the well‐being of the mother and/or the baby. Approximately 25%–30% of women undergo IOL, with significant country‐wide proportional variations [1].

Pharmacological prostaglandin‐based methods are still popular in most settings. However, there is a growing body of evidence supporting the use of mechanical methods (balloon catheters, osmotic cervical dilators) and the combination of mechanical and pharmacological methods [2, 3, 4, 5, 6, 7, 8, 9]. Currently, in most global settings, cervical ripening and IOL are typically performed after hospital admission. However, outpatient cervical ripening before IOL, utilising mechanical and pharmacological methods, is gaining popularity in some regions [10]. It is important to investigate the potential impacts of these outpatient interventions on effectiveness, safety, length of hospital stay, patient experience, and cost‐effectiveness.

However, the limited data on its effectiveness, safety, and patient perceptions, along with highly variable global adoption, remain significant challenges [11, 12]. Studies have also shown women's preferences for outpatient settings [13], potential cost‐savings [14] and positive insights from midwives [15]. A few aggregate data meta‐analyses, including a Cochrane review, have been published on outpatient cervical ripening [11, 16, 17]. Their results on the balloon catheter comparison in both outpatient and inpatient groups have shown probable beneficial outcomes for the outpatient setting. However, there is still uncertainty about the important outcomes, as these aggregate data meta‐analyses cannot reach an agreement. This is where an IPD meta‐analysis is needed.

We aimed to evaluate inpatient versus outpatient settings (including different pharmacologic and mechanical methods) in women having cervical ripening and IOL for the outcomes of vaginal birth, composite adverse perinatal outcome, and composite adverse maternal outcome.

## 2 Methods

This international collaborative IPD meta‐analysis followed a prospectively registered protocol (PROSPERO on 27‐04‐2022) and a statistical analysis plan produced in advance of data lock and analysis (Appendix S1). Findings are reported following the Preferred Reporting Items for Systematic Review and Meta‐Analyses of individual participant data (PRISMA‐IPD) Statement [18].

### 2.1 Search Strategy

The initial search was conducted in April 2022, and the search was updated in September 2022 and again in July 2024. Databases included Ovid MEDLINE, Ovid Embase, Ovid Emcare, CINAHL Plus, Scopus, Cochrane Pregnancy and Childbirth Group's Trials Register, the World Health Organisation (WHO) International Clinical Trials Registry Platform (ICTRP), and clinicaltrials.gov. The search strategy is outlined in Table S1. Reference lists of retrieved studies were searched further to identify eligible RCTs. The 2020 Cochrane review on ‘Home versus inpatient induction of labour for improving birth outcomes’ was also cross‐checked for any outstanding eligible RCTs [11]. There were no language restrictions, and all the published and unpublished studies were eligible. Two investigators (M.P. and F.C.) independently reviewed the identified titles and abstracts, followed by the full texts for eligibility, with disagreements solved by a third reviewer (W.L.).

### 2.2 Eligibility Criteria

RCTs comparing outpatient and inpatient cervical ripening and IOL were eligible for inclusion. Cluster randomised trials, cross‐over, and quasi‐experimental trials were excluded. All current pharmacological and mechanical methods used for cervical ripening and IOL were eligible. Only RCTs with active interventions in both arms were included, excluding trials involving placebo, expectant management, or physician's discretion. Pregnant individuals with an unfavourable cervix, as defined by the modified Bishop score, were eligible for inclusion [19]. In each RCT, singleton foetuses at or beyond 34 weeks of gestation were included, regardless of membrane status and previous caesarean delivery.

### 2.3 Data Access

We approached investigators of all potentially eligible RCTs to further confirm eligibility and requested those who authored eligible RCTs to share individual participant‐level data. Investigators' contact details were obtained through the published articles or the websites of their associated institutions, and invitations to participate were sent by e‐mail at least four times if there was no response. Where the primary or corresponding authors' contact details were unavailable or no response was obtained, attempts were made to contact other authors involved in the RCTs, and lead/co‐authors were copied in at least every month until March 2024. In addition to e‐mailing authors, we used social media platforms and contacted institutions or clinics by e‐mail or phone when there was no response. Our academic contacts in certain countries were called to contact the non‐responding authors/institutions. Journal editors or funding bodies were contacted as the last resort for some studies. Once RCT investigators responded that they were interested in participating, regular e‐mails were sent every month, coordinating the data‐sharing process and clarifying details. The author group followed the same process successfully for their previous IPD meta‐analysis projects [4, 20, 21].

RCT investigators supplied deidentified participant‐level data, which were harmonised and recoded according to the pre‐defined IPD meta‐analysis codebook (Appendix S2). Data were requested for all women randomised, even if excluded from original trial analyses. Where possible, the received data were examined for missing data, errors, internal consistency, consistency with the study publications, and pattern of treatment allocation and data presentation. Identified issues were communicated with RCT investigators for a solution before acceptance in the IPD meta‐analysis dataset. Eligible RCTs were also subjected to a trustworthiness assessment using the Trustworthiness in RAndomised Controlled Trials (TRACT) trustworthiness checklist [22, 23, 24].

### 2.4 Outcomes and Effect Modifiers

The primary outcomes (Table 1) included vaginal birth, a composite measure of adverse perinatal outcomes, and a composite measure of adverse maternal outcomes. The composite adverse perinatal outcome included at least one of stillbirth, neonatal death, neonatal Apgar score < 7 at 5 min, acidosis (arterial umbilical cord pH < 7.1), neonatal seizures, hypoxic–ischemic encephalopathy of any stage (HIE), neonatal intensive care unit (NICU) admission, meconium aspiration syndrome, neonatal infection either clinically suspected (as defined by neonatal antibiotic administration) or proven neonatal infection (culture‐proven), cord prolapse, endotracheal intubation, and external cardiac compressions. The composite adverse maternal outcome included any of the following: admission to the intensive care unit (ICU), maternal infection (temperature ≥ 38°C at any time during labour or delivery, antibiotics use or clinically diagnosed infection, such as endometritis), postpartum haemorrhage (PPH) ≥ 1000 mL, maternal death, and uterine rupture. When calculating composite outcomes, the presence of one or more of the pre‐defined components of the composite outcome was considered as the presence of composite perinatal or maternal outcomes.

**TABLE 1:** Inclusion and exclusion criteria of the included trials.

Study	Inclusion criteria	Exclusion criteria
Use of balloon catheters in both outpatient and inpatient groups		
Policiano, 2016—Portugal	Primip + multip with a singleton, vertex presentation, Bishop score less than 6, ≥ 41 weeks of gestation and high‐risk pregnancy indicating elective induction at earlier gestational age	Non‐vertex presentation, an indication for elective CS, spontaneous labour, (AFI ≥ 25), nonreassuring cardiotocograph, multiple pregnancies, rupture of membranes, active vaginal bleeding, indication for prophylaxis of Streptococcus group B or HIV infection, cervical injury or previous caesarean section
Kuper, 2018—United States	Multiparous low‐risk women with a singleton, vertex presentation, Bishop score less than 7, at least 18 years old with their estimated gestational age between 39 and 42 weeks of gestation, reliable transportation, access to a telephone and resides less than 30 min from the hospital, cervix 3 cm or less or, if 2–3 cm dilated, less than 80% effaced and reassuring fetal heart rate monitoring	FGR, oligohydramnios, polyhydramnios, prior CS or uterine surgery of the myometrium, chronic hypertension requiring more than one antihypertensive medication, diabetes mellitus (other than diet‐controlled gestational diabetes), gestational hypertension, pre‐eclampsia, hepatitis B or C or HIV, fetal anomalies or IUFD, non‐reassuring CTG, established labour, latex allergy, non‐English‐speaking, contraindications to vaginal delivery, or conditions requiring immediate hospitalisation and serious medical conditions precluding outpatient cervical ripening
Chen, 2019—Australia^a^	Primip + multip low‐risk women with a singleton, vertex presentation, 37–42 weeks of gestation, Bishop score less than 7 with intact membranes	Inadequate transport, CS scar, any contraindication to vaginal delivery or induction
Ausbeck, 2020—United States	Primiparous low‐risk women with a singleton, vertex presentation, Bishop score less than five and cervical dilation 2 cm or less immediately before randomisation, 39–41 6/7 weeks of gestation, at least 18 years old, reliable transportation, telephone access and lives within 30 min of the hospital	IUFD, major anomalies, FGR, suspected macrosomia, oligohydramnios, polyhydramnios, nonreassuring fetal status (BPP score 6/10 or less), prior uterine surgery involving the myometrium, gestational hypertension or pre‐eclampsia, uncontrolled pregestational diabetes mellitus, hepatitis B or C or HIV, latex allergy
Haavisto, 2020—Finland	Primip + multip low‐risk women with a singleton, vertex presentation, Bishop score less than 6, 37–41 5/7 weeks of gestation, uncomplicated pregnancy, intact membranes, normal CTG, previous CS, live within 30 min drive from hospital, sufficient knowledge of the Finnish language	Medical conditions or pregnancy complications: medically treated gestational diabetes, hypertension, preeclampsia or FGR, or other signs of fetal distress
Kohari, 2021—United States^b^	Primip + multip low‐risk women with a singleton, vertex presentation, Bishop score less than 6, > 37 weeks of gestation, intact membranes, normal vital signs and at least 18 years old	Any contraindication for vaginal birth, multiple gestation, history of CS, oligohydramnios/polyhydramnios/anhydramnios (MVP < 2 cm), rupture of membranes, poorly controlled diabetes, poorly controlled chronic hypertension, gestational hypertension or preeclampsia, vaginal bleeding, any conditions that require continuous electronic fetal monitoring (intrahepatic cholestasis of pregnancy, FGR, abnormal non‐stress test), fetal anomaly, IUFD, HIV infection, presence of genital herpetic lesion, history of substance abuse during this pregnancy, history of precipitous delivery, poor access to care, inability to give informed consent, strong preference for inpatient and not literate on English or Spanish
Pierce‐Williams, 2022—United States	Primip + multip women with a singleton, vertex presentation, ≥ 37 weeks, Bishop score less than 6	Gestational hypertension, preeclampsia, non‐reassuring fetal testing, multi‐fetal gestation, oligohydramnios, fetal anomaly, less than 37 weeks, Bishop score > 6, ruptured membranes, contraindication to vaginal delivery including: active Herpes lesion, HIV VL > 1000 copies/mL, placenta previa, vasa previa, breech presentation, prior classical CS or transfundal myomectomy, poor access to telephone or transportation, latex allergy, any other condition for which the managing physician or investigator deem outpatient management inappropriate, currently participating in another clinical trial
Use of balloon catheter in the outpatient groups versus vaginal dinoprostone in the inpatient group		
Henry, 2013—Australia	Primip + multip low‐risk women with a singleton, vertex presentation, ≥ 18 years old, ≥ 37 weeks of gestation, Bishop Score < 7 and cervical dilation < 2 cm, normal CTG	Unsuitable for outpatient management, Unsuitable for randomisation to either PGE2 (e.g., previous CS) or catheter use (e.g., latex allergy), or prior attempted IOL in this pregnancy, Bishop score ≥ 7 or cervical dilatation ≥ 2 cm, ruptured membranes, or evidence of regular uterine contractions at time of booked induction, multiple pregnancy or non‐vertex presentation, Unable to give informed consent
Beckmann, 2019—Australia	Primip + multip low‐risk women with a singleton, vertex presentation, ≥ 37 weeks of gestation, undergoing IOL for low‐risk indications including post‐term, ‘social’ or ‘elective’ reasons and advanced maternal age (≥ 40 years) and Bishop Score < 7	Major congenital abnormality, SGA, previous CS, Bishops score ≥ 7, high head (≥ 4/5), residing > 60 min from hospital, or any contraindication to vaginal birth
Wise, 2023—New Zealand	Primip + multip low‐risk women with a singleton, vertex presentation, ≥ 37 weeks of gestation, intact membranes, normal non‐stress test, Bishop score < 7, and able to remain within 1 h of the hospital with someone who could speak sufficient English	Previous CS, major fetal anomaly, suspected severe FGR, and a maternal or fetal condition for which the clinician felt that outpatient care was contraindicated
Use of cervical osmotic dilators (Dilapan‐S) in both outpatient and inpatient groups		
Saad, 2022—United States	Primip + multip low‐risk women with a singleton, live, vertex presentation, ≥ 37 weeks of gestation, intact membranes, a cervix not more than 3 cm dilated and not more than 60% effaced and ≥ 18 years old	Active labour, active genital herpes, chorioamnionitis, transfundal uterine or cervical surgery, previous CS, nonreassuring fetal status, Need for continuous maternal or fetal monitoring during ripening, contraindication for vaginal delivery, active vaginal bleeding, abnormal placental location or adherence (placenta previa or unresolved low‐lying placenta), EFW > 5000 g (nondiabetic) or > 4500 g (diabetic), FGR, oligohydramnios, fetal anomaly, need for inpatient care (e.g., hypertension, insulin‐dependent diabetes), limited access to a telephone, lack of support person for accompany to hospital or cannot be placed in the hotel (if they lived more than 60 min)

Abbreviations: AFI, amniotic fluid index; CS, caesarean section; CTG, cardiotocogram; EFW, estimated fetal weight; FGR, fetal growth restriction; HIV, human immunodeficiency virus; IUFD, intrauterine fetal death; MVP, maximum vertical pocket; PGE2, prostaglandin E2; SGA, small for gestational age.

^a^ This trial was published as an abstract and the data collection continued after the abstract, giving a slightly larger sample here.

^b^ The datasheet was from an unpublished trial that was halted due to poor recruitment.

Secondary outcomes are found in Table 1. Delivery outcomes included the mode of delivery (unassisted/spontaneous vaginal, assisted/instrumental vaginal, caesarean), indication for an instrumental vaginal delivery, indication for caesarean delivery (failure to progress or fetal compromise), and the cumulative rate of vaginal and total number of days of inpatient stay. Labour progression outcomes included uterine tachysystole, uterine hyperstimulation, the need for oxytocin, the presence of meconium‐stained amniotic fluid, and the need for a second method for cervical ripening. Secondary neonatal safety outcomes assessed individually included Apgar score < 7 at 5 min, arterial umbilical cord pH < 7.10, and NICU admission. Secondary maternal safety outcomes assessed individually included maternal infection, severe PPH (≥ 1000 mL) and maternal ICU admission. Pain during cervical ripening and overall satisfaction were also assessed under patient experience outcomes. The international multistakeholder Delphi study on the core outcome set for induction labour (COSIOL) was taken into consideration in defining these outcomes [25, 26].

Potential effect modifiers of interest for the primary outcome of vaginal birth included parity, maternal age, body mass index (BMI), initial Bishop score, and indications for labour induction (Table 1).

### 2.5 Risk of Bias of Included Studies and Certainty of Evidence

The Risk of Bias (RoB) was independently assessed by two researchers (M.P. and F.C.) using the Cochrane RoB‐2 tool [27]. Disagreements were resolved by consensus; if the information was insufficient, trialists were sought for clarification. We used the GRADE (Grading of Recommendations Assessment, Development and Evaluation) approach to assess the overall certainty of the evidence for the primary outcomes [28].

### 2.6 Data Synthesis

For each outcome, an intention‐to‐treat analysis was performed using all available data comparing the outpatient setting to the inpatient setting. Analysis was conducted for all the methods as an overall comparison and for the use of balloon catheters in both groups separately. The inpatient setting was considered the reference group for all outcomes. A pre‐defined statistical analysis plan has been added as Appendix S1.

Our primary analysis strategy involved a two‐stage meta‐analysis method to synthesise the IPD. However, for outcomes that had no events in any intervention group of any included RCT, a one‐stage method was used as the primary analysis. In the first step of the two‐stage method, we compared outcomes between the outpatient and inpatient settings for each included study, adjusting for maternal age and parity. For binary outcomes, we calculated adjusted odds ratios (aORs) along with 95% confidence intervals (CIs) using logistic regression. For the cumulative rate of vaginal births, which is a time‐to‐event outcome, we estimated sub‐distribution hazard ratios (sHRs) and 95% CIs using the sub‐distribution hazard (Fine and Grey) model, which considered caesarean birth as a competing risk. The interpretation of this model focuses on the sub‐distribution hazard, which may not have a clear causal interpretation [29]. The total number of days of hospital stay for the mother and neonate was analysed using negative binomial regression. In the second step, we combined the generated relative estimates using random‐effects models (restricted maximum likelihood estimator with Hartung‐Knapp‐Sidik‐Jonkman variance correction) [30, 31]. For the one‐stage method, we used multilevel mixed‐effects logistic regression (a stratified intercept by study and a random treatment effect, covariates as fixed effects, and maximum likelihood estimator) adjusting for maternal age and parity [31]. We tested treatment‐covariate interactions for vaginal birth using interaction terms between treatment and potential effect modifiers, also adjusting for maternal age and parity. We tested multiplicative treatment‐covariate interactions for vaginal birth using interaction terms between treatment and potential effect modifiers, also adjusting for maternal age and parity. A product of treatment*covariate was included in the regression models for each RCT. The interaction ORs were pooled across studies using the same two‐stage methods as the main effects of the treatment. Compared to the additive scale, the multiplicative interaction scale is more stable across studies and does not depend on baseline risk levels, which vary substantially across studies [32, 33]. Therefore, the multiplicative interaction scale was chosen for its relevance for clinical interpretation of effect modification. To avoid ecological bias, we only considered within‐trial interaction. All variables besides the identification variable were checked for missing values and entries outside the expected ranges. In the event of missing values for covariates or potential effect modifiers in any RCT, we performed multiple imputations using chained equations (10 imputed datasets) within the RCT before the meta‐analysis.

The funnel plot of all RCTs was plotted to assess the publication bias. We extracted summary data for vaginal birth from all eligible RCTs, including those that did not contribute to IPD and those with IPD‐shared RCTs. We performed an aggregate data meta‐analysis using the same random‐effects model to assess the risk of data unavailability bias of the IPD meta‐analysis. Sensitivity analyses for the primary outcomes were conducted using one‐stage meta‐analyses to assess the impact of the meta‐analytical method on the pooled estimates. Additionally, an as‐treated analysis was performed to compare with the intention‐to‐treat analysis.

Statistical analyses were performed using the R statistical environment (R Foundation for Statistical Computing, Vienna, Austria) [34]. The ‘meta’ package was used for two‐stage meta‐analysis and aggregate data meta‐analysis [35], the ‘lme4’ package was used for one‐stage meta‐analysis [36], the ‘cmprsk’ package was used for competing risk analysis [37], the ‘MASS’ package was used for negative binomial regression [38] and the ‘lmtest’ package was used for the likelihood ratio test for comparing two nested models in treatment‐covariate interaction analysis [39].

## 3 Results

### 3.1 Study Selection and Participants

**FIGURE 1:**
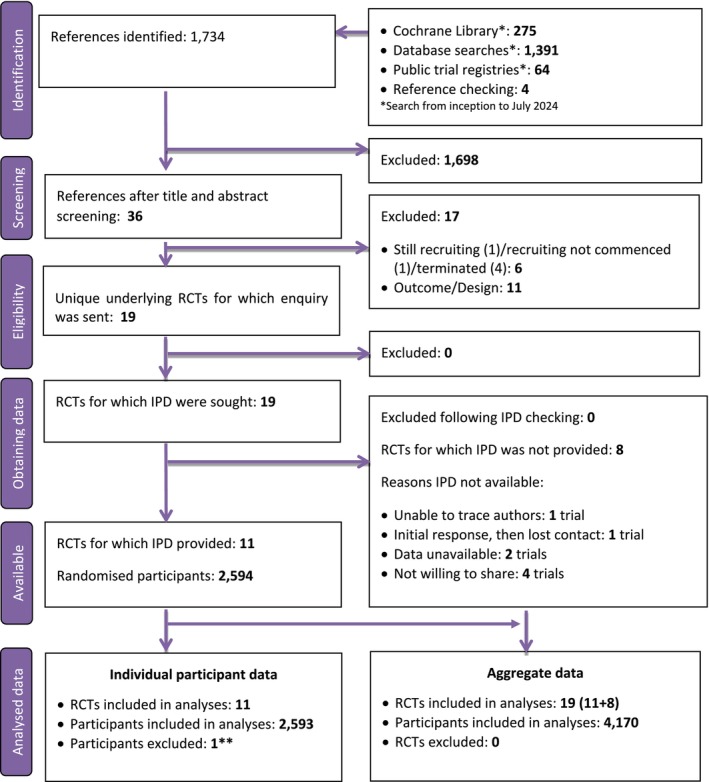
Trial identification (PRISMA‐IPD flow diagram). RCTs, Randomised controlled trials. **Early withdrawal in Saad et al. did not qualify for intention‐to‐treat analysis.

Our systematic search identified 1734 unique references. The PRISMA‐IPD flow diagram is shown in Figure 1. After screening, 19 RCTs were potentially eligible for inclusion. Enquiries were sent, and eight RCTs did not participate in this IPD meta‐analysis [40, 41, 42, 43, 44, 45, 46, 47]. The authors of these eight RCTs did not share data for the following reasons: four author groups declined to share the data, raw data were unavailable from two RCTs, one author group initially responded but then discontinued contact and one author group was unable to be traced. Table S3 shows characteristics of RCTs that did not contribute to the IPD meta‐analysis. After trustworthiness assessment, non‐shared RCT, Rahman et al. [46] were identified as ‘not‐meeting trustworthiness criteria’.

### 3.2 Study Characteristics

Following data checks, we included all 11 studies that shared IPD (2593 women) in this meta‐analysis [3, 48, 49, 50, 51, 52, 53, 54, 55, 56, 57, 58]. Out of 11 RCTs comparing outpatient versus inpatient methods for cervical ripening, seven RCTs used balloon catheters for both groups [17, 48, 50, 51, 52, 53, 55], with three also using intravenous oxytocin in addition to the balloon catheters for the inpatient group [17, 51, 52]. Another three RCTs used balloon catheters for the outpatient group and vaginal dinoprostone for the inpatient group [49, 54, 58] and one RCT used cervical osmotic dilators (Dilapan) for both groups [3]. Table 2 summarises the characteristics of the 11 RCTs included.

**TABLE 2:** Characteristics of included trials.

Author	Year	Country	Outpatient arm	Inpatient arm	No. of participants
Use of balloon catheters in both outpatient and inpatient groups					
Policiano	2016	Portugal	Outpatient 16 F standard latex Foley Catheter inflated with 40 mL of sterile water for a maximum of 24 h; discharged after reassuring CTG	Inpatient 16 F standard latex Foley Catheter inflated with 40 mL of sterile water for a maximum of 24 h	130
Kuper	2018	United States	Outpatient 16 F standard latex Foley Catheter inflated with 30 mL of sterile water for a maximum of 24 h; discharged after reassuring CTG	Inpatient 16 F standard latex Foley Catheter inflated with 30 mL of sterile water for a maximum of 24 h. Oxytocin infusion was begun concurrently with the Foley catheter	129
Chen^a^	2019	Australia	Outpatient double‐balloon catheter (Cook) inflated with 80 mL of sterile water for a maximum of 12 h; discharged after reassuring CTG	Inpatient double‐balloon catheter (Cook) inflated with 80 mL of sterile water for a maximum of 12 h	38
Ausbeck	2020	United States	Outpatient 16 F standard latex Foley Catheter inflated with 30 mL of sterile water for a maximum of 24 h; discharged after reassuring CTG	Inpatient 16 F standard latex Foley Catheter inflated with 30 mL of sterile water for a maximum of 24 h. Oxytocin infusion was begun concurrently with the Foley catheter	126
Haavisto	2020	Finland	Outpatient double‐balloon catheter (Cook) inflated with 80 mL of sterile water for a maximum of 24 h	Inpatient double‐balloon catheter (Cook) inflated with 80 mL of sterile water for a maximum of 24 h	113
Kohari^b^	2021	United States	Outpatient standard latex Foley Catheter for a maximum of 12 h	Inpatient standard latex Foley Catheter for a maximum of 12 h	53
Pierce‐Williams	2022	United States	Outpatient standard latex Foley Catheter for a maximum of 12 h	Outpatient standard latex Foley Catheter for a maximum of 12 h. Oxytocin infusion was begun concurrently with the Foley catheter	30
Use of balloon catheter in the outpatient groups versus vaginal dinoprostone in the inpatient group					
Henry	2013	Australia	Outpatient 16 F standard latex Foley Catheter inflated with 30 mL of sterile water; discharged after reassuring CTG	Inpatient 1 mg PGE2 gel and repeated in 6 h, if required	101
Beckmann	2019	Australia	Outpatient double‐balloon catheter (Cook) inflated with 80 mL of sterile water for a maximum of 12 h; post‐balloon CTG was not routinely done, only 30 min of observation	Inpatient PGE2 either as 2 mg Dinoprostone vaginal gel (Prostin) or 10 mg Dinoprostone controlled‐release vaginal tape (Cervidil) and repeated two more doses 6 hourly if required	448
Wise	2023	New Zealand	Outpatient single 50 mL Foley balloon catheter (Bard, 2‐way, 20F) for 18 to 24 h; not stated about post‐balloon monitoring	Inpatient PGE2 either as Dinoprostone gel (Prostin E2) or a controlled‐release pessary (Cervidil)	1087
Use of cervical osmotic dilators (Dilapan‐S) in both outpatient and inpatient groups					
Saad	2022	United States	Outpatient osmotic dilators were left for at least 12 h: discharged after reassuring CTG	Inpatient osmotic dilators were left for at least 12 h	338

Abbreviations: BS, Bishops' score; CS, caesarean section; CTG, cardiotocography; Multip, multiparous women; NS, not stated; Primip, primiparous women; ROM, rupture of membranes.

^a^ This trial was published as an abstract and the data collection continued after the abstract, giving a slightly larger sample here.

^b^ The datasheet was from an unpublished trial that was halted due to poor recruitment.

### 3.3 Quality and Data Trustworthiness of Included Studies

**FIGURE 2:**
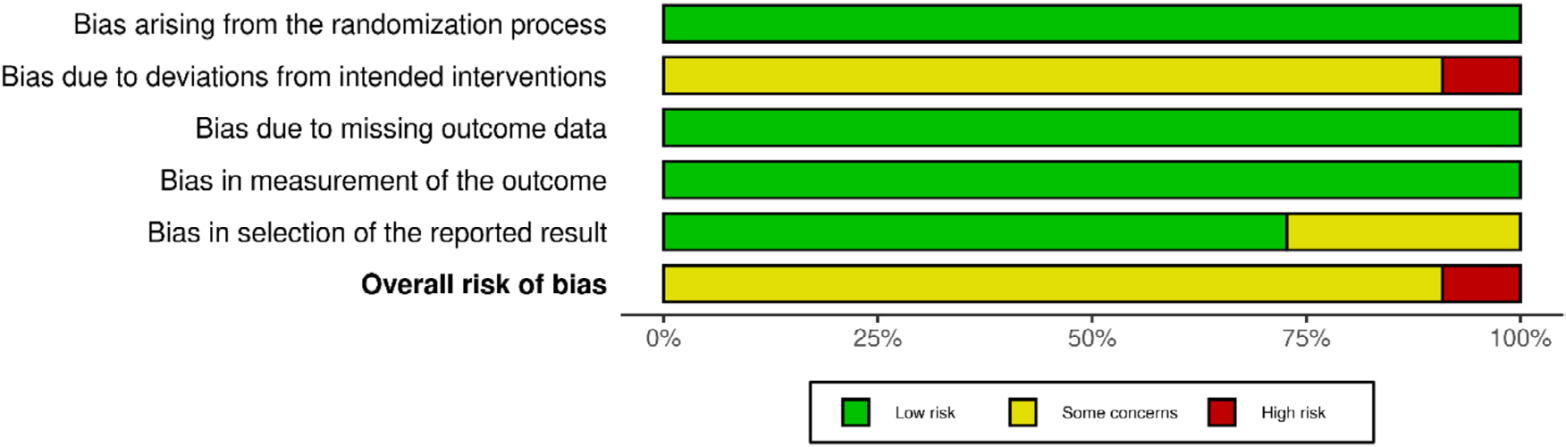
Risk of bias assessment of included RCTs in the intention‐to‐treat population.

In screening for risk of bias, RCTs were mostly identified as having overall ‘some concerns’ (Figure 2 and Figure S1), largely owing to the inability to blind the participants and/or investigator due to the nature of the intervention. One RCT had an overall ‘high risk’ owing to per protocol rather than intention‐to‐treat analysis [59]. All the included RCTs were published between 2013 and 2023. All except three RCTs had prospective trial registration; two trials had a retrospective registration [50, 56], and another had no registration [53]. However, after IPD checking, there was no reason to exclude these three RCTs. Figure S2 shows the risk of bias summary for trials that did not provide IPD.

### 3.4 Descriptive Analysis of Participants

Of the 2593 included participants, 1284 women (49.5%) were allocated to outpatient cervical ripening, and 1309 women (50.5%) to inpatient cervical ripening. Baseline participant characteristics of each included RCT are available in Table S4.

### 3.5 Synthesis of Results

#### 3.5.1 Primary Outcomes

##### 3.5.1.1 Overall Comparison of Outpatient and Inpatient Cervical Ripening

**FIGURE 3:**
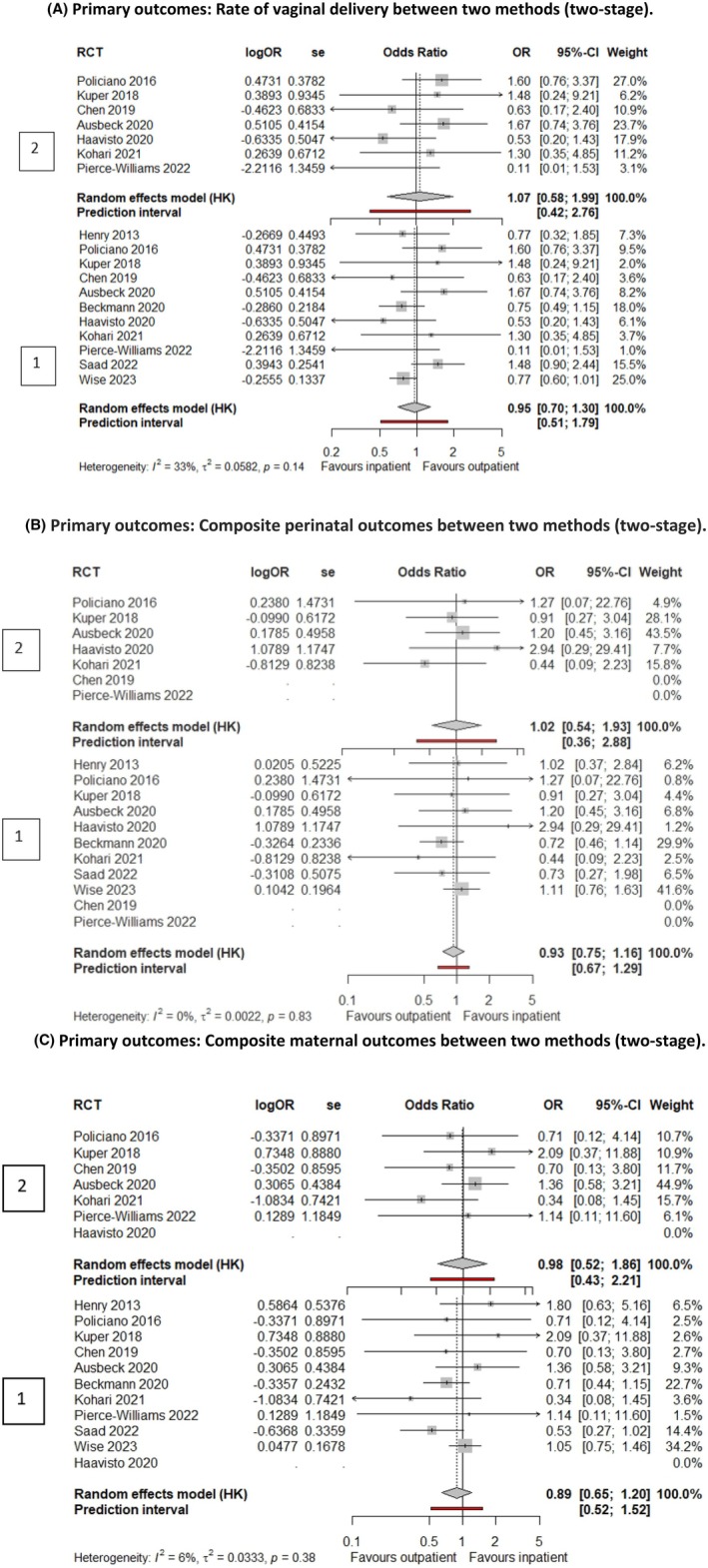
Forest plots showing the results of the primary outcomes of this IPD meta‐analysis (Plot 1: Shows overall comparison with all the methods and Plot 2: Shows the use of balloon catheters in both settings).

The odds of vaginal birth were not significantly different between outpatient and inpatient groups (11 RCTs, 2584 women, 67.8% vs. 70.2%, aOR 0.95, 95% CI 0.70; 1.30, *p* = 0.74, *I* ^2^ = 33%: moderate certainty evidence as downgraded due to inconsistency, Figure 3A). The odds of composite adverse perinatal outcome were comparable between the two groups (9 RCTs, 2525 women, 11.1% vs. 11.7%, aOR 0.93, 95% CI 0.75; 1.16, *p* = 0.48, *I* ^2^ = 0%: moderate certainty evidence as downgraded due to concerns on data completeness, Figure 3B) and composite adverse maternal outcome was also comparable between the two groups (10 RCTs, 2480 women, 14.3% vs. 15.4%, aOR 0.89, 95% CI 0.65; 1.20, *p* = 0.39, *I* ^2^ = 6%: moderate certainty evidence as downgraded due to concerns on data completeness, Figure 3C).

##### 3.5.1.2 Use of Balloon Catheters in Both Outpatient and Inpatient Settings

The odds of vaginal birth were similar between outpatient and inpatient groups (7 RCTs, 616 women, 77.9% vs. 76.3%, aOR 1.07, 95% CI 0.58; 1.99, *p* = 0.79, *I* ^2^ = 23%: low certainty evidence as downgraded due to concerns on data completeness and imprecision, Figure 3A). The odds of composite adverse perinatal composite outcome (5 RCTs, 551 women, 8.3% vs. 7.2%, aOR 1.02, 95% CI 0.54; 1.93, *p* = 0.94, *I* ^2^ = 0%: low certainty evidence as downgraded due to concerns on data completeness and imprecision, Figure 3B), and composite adverse maternal outcome were comparable between the two groups (6 RCTs, 506 women, 10.5% vs. 9.8%, aOR 0.98, 95% CI 0.52; 1.86, *p* = 0.94, *I* ^2^ = 0%: low certainty evidence as downgraded due to concerns on data completeness and imprecision, Figure 3C).

#### 3.5.2 Secondary Outcomes

##### 3.5.2.1 Overall Comparison of All the Methods for Cervical Ripening and Labour Induction

Table 3 and Figures S3–S10 show the secondary outcomes results for the overall comparison with all the methods for outpatient versus inpatient cervical ripening. There was no difference in the rates of unassisted vaginal births (Table 3 and Figure S4) between the two methods. Caesarean delivery for fetal distress and caesarean delivery for failure to progress (Table 3) were comparable between the two settings. Time‐to‐event analysis of the subdistribution hazard ratio of vaginal birth, accounting for caesarean delivery as a competing event, did not show a statistically significant difference and demonstrated substantial heterogeneity (Table 3 and Figure S5). There was no significant difference in maternal or neonatal inpatient length of stay (Table 3). Women in the outpatient group were significantly more likely to receive oxytocin once they returned to the hospital (11 RCTs, 2587 women, aOR 1.72, 95% CI 1.41; 2.09, *p* < 0.01).

**TABLE 3:** Outpatient compared to inpatient settings for cervical ripening and induction of labour for secondary outcomes—overall comparison.

Secondary outcome	No. of trials	No. of women	Crude incidence (%)—outpatient versus inpatient methods	aOR (95% CI)	*I* ^2^ (95% CI)	Analysis method^a^	Certainty of the evidence (GRADE)
Delivery outcomes							
Caesarean delivery	11	2584	31.8 vs. 29.7	1.02 (0.75; 1.37)	30 (0.0; 66)	Two‐stage	⊕⊕⊕⊕ High
Caesarean delivery for failure to progress	11	2584	18.6 vs. 16.9	1.04 (0.77; 1.42)	—	One‐stage	⊕⊕⊕⊕ High
Caesarean delivery for fetal distress	11	2584	10.3 vs. 10.2	0.99 (0.77; 1.29)	—	One‐stage	⊕⊕⊕⊕ High
Unassisted vaginal birth	11	2584	51.1 vs. 55.5	0.91 (0.64; 1.29)	49 (0.0; 75)	Two‐stage	⊕⊕⊕⊝^e^ Moderate
Instrumental vaginal birth	11	2584	16.7 vs. 14.8	1.15 (0.92; 1.43)	—	One‐stage	⊕⊕⊕⊝^e^ Moderate
Instrumental vaginal birth for failure to progress in the second stage	11	2475	5.4 vs. 4.6	1.19 (0.82; 1.74)	—	One‐stage	⊕⊕⊕⊝^e^ Moderate
Instrumental vaginal birth for fetal distress	11	2475	9.2 vs. 8.4	1.10 (0.83; 1.47)	—	One‐stage	⊕⊕⊕⊝^e^ Moderate
Time to vaginal birth (hours)	10	2379	30.1 vs. 26.3^c^	sHR 0.76 (0.44; 1.31)	89 (82; 93)	Two‐stage	⊕⊕⊕⊝^e^ Moderate
Total maternal hospital stay (hours)	6	2032	67.8 vs. 71.0^c^	Incidence rate ratio 0.97 (0.94; 1.01)	0 (0; 75)	Two‐stage	⊕⊕⊕⊝^e^ Moderate
Total neonatal hospital stay (hours)	3	1633	46.1 vs. 41.3^c^	Incidence rate ratio 1.10 (0.91; 1.33)	19 (0; 92)	Two‐stage	⊕⊕⊝⊝ Low^d^, ^f^
Labour progression outcomes							
Uterine tachysystole	3	419	1.5 vs. 3.3	0.32 (0.04; 2.62)	0 (0; 90)	Two‐stage	⊕⊝⊝⊝ Very low^d^ ^–^ ^f^
Uterine hyperstimulation	5	2024	6.9 vs. 7.1	0.94 (0.67; 1.34)	—	One‐stage	⊕⊕⊝⊝ Low^d^, ^f^
Oxytocin augmentation	11	2587	78.7 vs. 69.7	1.72 (1.41; 2.09)	—	One‐stage	⊕⊕⊕⊕ High
Meconium‐stained amniotic fluid	5	794	13.6 vs. 14.4	0.92 (0.61; 1.37)	—	One‐stage	⊕⊕⊝⊝ Low^d^, ^f^
Use of epidural analgesia	9	2422	74.6 vs. 67.7	1.45 (1.16; 1.80)	0 (0; 65)	Two‐stage	⊕⊕⊕⊝ Moderate^d^
Use of more methods for cervical ripening (second methods)	6	2149	21.9 vs. 11.7	1.58 (0.77; 3.25)	81 (60; 91)	Two‐stage	⊕⊕⊝⊝ Low^d^, ^f^
Maternal safety outcomes							
Maternal infection	11	2592	8.4 vs. 7.9	1.10 (0.76; 1.60)	—	One‐stage	⊕⊕⊕⊝ Moderate^d^
Severe postpartum haemorrhage	10	2470	7.3 vs. 9.3	0.76 (0.56; 1.01)	—	One‐stage	⊕⊕⊕⊝ Moderate^e^
Maternal ICU admission	8	2349	0.1 vs. 0.2	0.50 (0.05; 5.56)		One‐stage	⊕⊝⊝⊝ Very low^d^ ^–^ ^f^
Neonatal safety outcomes							
Stillbirths	—	—	No stillbirths were reported in either group	—	—	—	—
Neonatal deaths	—	—	No neonatal deaths were reported in either group	—	—	—	—
Cord prolapse	5	1920	0.3 vs. 0.3	1.00 (0.16; 6.10)	—	One‐stage	⊕⊝⊝⊝ Very low^d^ ^–^ ^f^
Apgar score < 7 at 5 min	11	2581	2.2 vs. 1.5	1.44 (0.80; 2.57)	—	One‐stage	⊕⊕⊝⊝ Low^d^, ^f^
Arterial umbilical cord pH < 7.10	7	1466	3.8 vs. 6.3	0.58 (0.36; 0.94)	—	One‐stage	⊕⊕⊕⊝ Moderate^e^
NICU admission	10	2476	6.3 vs. 6.4	0.97 (0.70; 1.34)	—	One‐stage	⊕⊕⊕⊝ Moderate^e^
Patient satisfaction outcomes							
Pain during cervical ripening^b^	4	589	2.7 vs. 3.0^c^	SMD 0.16 (−0.59; 0.90)	17 (0; 87)	Two‐stage	⊕⊝⊝⊝ Very low^d^ ^–^ ^f^
Overall satisfaction score^b^	3	464	6.0 vs. 6.0^c^	SMD 0.14 (−1.59; 1.87)	43 (0; 83)	Two‐stage	⊕⊝⊝⊝ Very low^d^ ^–^ ^f^

Abbreviations: aOR, adjusted odds ratio; ICU, intensive care unit; NICU, neonatal intensive care unit; RR, Rate ratio; sHR, sub‐distribution hazard ratio; SMD, standardised mean difference.

^a^ Two‐stage as the primary strategy, one‐stage used when zero events are encountered in any arm of any included study.

^b^ Pain and satisfaction outcomes have been reported using visual analogue scales [1, 2, 3, 4, 5, 6, 7, 8, 9, 10]. All analyses were adjusted for maternal age and parity.

^c^ Median values have been reported.

^d^ Downgraded one level for imprecision.

^e^ Downgraded one level for inconsistency.

^f^ Downgraded one level for concerns about data completeness.

There was high imprecision due to wide CIs between both settings for cord prolapse, Apgar score, and admission to NICU between the two settings (Table 3). There was also low certainty regarding the impact of either setting on maternal infection and maternal intensive care admissions (Table 3). The outpatient setting resulted in significantly lower acidosis in umbilical arterial cord blood (7 RCTs, 1466 women, aOR 0.58, 95% CI 0.36; 0.94, *p* = 0.03), a trend towards less severe postpartum haemorrhage (10 RCTs, 2470 women, aOR 0.76, 95% CI 0.56; 1.01, *p* = 0.06) and more epidural analgesia (9 RCTs, 2422 women, aOR, 1.45, 95% CI 1.16; 1.80, *p* = 0.005) than those in the inpatient group (Table 3 and Figure S9). There was no significant difference in patient experience outcomes (Table 3 and Figures S7 and S8).

##### 3.5.2.2 Use of Balloon Catheters in Both Outpatient and Inpatient Settings

Table S5 and Figures S11–S13 show the results of secondary outcomes with the use of balloon catheters in outpatient and inpatient groups. The above‐reported adverse outcomes with all the methods in the inpatient group regarding acidosis and severe postpartum haemorrhage were not observed with the use of balloon catheters in both groups. The estimates for the differences in the rates of acidosis in umbilical arterial cord blood and severe postpartum haemorrhage were uncertain between the two groups and had low precision (Table S5). The oxytocin use was comparable (Table S5). Women in the outpatient group with a balloon catheter used less epidural analgesia (5 RCTs, 448 women, aOR 0.67, 95% CI 0.48; 0.93, *p* = 0.03) than those in the inpatient group with a balloon catheter.

There was no statistically significant difference in the rates of unassisted vaginal births (Table S5 and Figure S12) between the two groups. We found no statistically significant difference in caesarean delivery for fetal distress or caesarean delivery for failure to progress (Table S5). There was high imprecision in the results for instrumental vaginal births for fetal distress and instrumental vaginal births for failure to progress (Table S5). Time‐to‐event analysis for the hazard ratio of vaginal birth demonstrated substantial heterogeneity, with wide CIs reflecting uncertainty in the timing of vaginal birth (Table S5 and Figure S13). There was insufficient data on inpatient length of stay. The available evidence was too imprecise to determine whether there were clinically significant differences between groups for cord prolapse, Apgar score < 7 at 5 min, admission to NICU, and maternal infection (Table S5). There was insufficient data regarding rates of maternal intensive care admissions. There was insufficient data on patient experience outcomes.

### 3.6 Intervention‐Covariate Interactions

No differential impact of treatment on the rate of vaginal birth (Table S6) was identified for maternal parity (nulliparous versus multiparous, 5 RCTs, 2116 women, p for interaction = 0.37), maternal age (11 RCTs, 2593 women, p for interaction = 0.33), gestational age at delivery (10 RCTs, 2563 women, p for interaction = 0.33) or initial Bishop score (9 RCTs, 1468 women, p for interaction = 0.63). There was an increased, but not statistically significant, effect on the potential impact of BMI (11 RCTs, 2593 women, p for interaction = 0.09). Therefore, a sub‐group analysis of the primary outcomes for three BMI categories (Underweight/Normal weight: BMI < 25 kg/m^2^, Overweight: BMI 25–29.9 kg/m^2^, and Obese: BMI ≥ 30 kg/m^2^) was performed. In the overall comparison of all the methods in both groups (Figure S14A–C) and with the use of balloon catheters in both groups (Figure S15A–C), there was no differential impact according to the three BMI categories on all three primary outcomes. Both strata‐specific and joint effects, with and without treatment*covariate interactions have been presented in Tables S6 and S7.

### 3.7 Publication and IPD Unavailability Biases

The funnel plot of all RCTs (participating and non‐participating) shows no discernible asymmetry, suggesting a low risk of publication bias (Figure S16). We could extract summary data for vaginal birth from six RCTs that did not share data (Figure S17) and 11 shared RCTs. Data on vaginal delivery could not be extracted from two non‐shared RCTs as not reported [45, 47]. Aggregate data meta‐analysis combining all the RCTs found comparable vaginal delivery between the outpatient and inpatient groups (17 RCTs, 4089 women, OR 1.00, 95% CI 0.83; 1.21, *p* = 0.99, *I* ^2^ = 18%) (Tables S8 and S9).

### 3.8 Sensitivity Analyses

Sensitivity analyses for primary outcomes using one‐stage models produced similar findings (Table S8). Similarly, ‘As Treated’ analyses of the primary outcomes (Table S9) for seven of the included RCTs did not lead to different conclusions compared to the main analyses for vaginal delivery, perinatal and maternal composite adverse outcomes.

## 4 Discussion

### 4.1 Main Findings

This IPD meta‐analysis, which included 11 RCTs with 2593 women having cervical ripening and IOL, found that there was no difference in vaginal delivery, perinatal and maternal safety between the outpatient and inpatient settings. Outpatient cervical ripening with a mechanical method showed significantly less acidosis in the babies while more use of epidural compared to inpatient with any method. Women in the outpatient group were significantly more likely to receive oxytocin once they returned to the hospital. The rate of severe postpartum haemorrhage (≥ 1000 mL) was lower in the outpatient group, but this did not reach statistical significance. There was no difference in induction to interval, inpatient hospital stay or satisfaction scores.

### 4.2 Strengths and Limitations

The main strengths of this work are the predefined analysis plan, a sample size of 2593 randomised women, and the use of a thoroughly cleaned IPD database. The IPD meta‐analysis study design improved the statistical power and enabled outcome harmonisation of composite outcomes and treatment–covariate interaction analysis. Eighteen out of 19 trial investigators responded to the data‐sharing requests for this IPD meta‐analysis, and we have included 62.2% of the published data on this topic. The central coordinating team continuously collaborated with trial investigators to ensure data consistency and quality. Ten out of 11 of the included RCTs had a low risk of bias in all domains except for the lack of blinding owing to the nature of the intervention. The remaining RCT showed a ‘high risk’ of bias as the data were only available for the per‐protocol analysis due to a large number of losses to follow‐up [54]. We adjusted clinical factors associated with IOL effectiveness and outcomes (age and parity) and explored the heterogeneity of treatment effects by investigating interactions and subgroup effects. Conducting high‐quality RCTs and sharing the raw data for IPD meta‐analysis not only minimises research waste but also makes the evidence more trustworthy. We acknowledge the loss of IPD from eight RCTs despite active efforts for more than 2 years to obtain their data. Three of eight non‐shared RCTs were published before 2010, and their data were unlikely to be available.

### 4.3 Interpretation

The existing aggregate data meta‐analyses comparing outpatient and inpatient IOL have shown comparable findings to each other [11, 16, 17]. In summary, they have reported a lower likelihood of caesarean delivery in the outpatient group and a reduction in inpatient hospital stay with no significant adverse maternal or perinatal safety concerns. Importantly, in a study by Dong et al., there were more suspicious fetal heart rate tracings (30.6% vs. 21.4%) in the outpatient group [16]. However, all three of these reviews were hampered by low certainty of evidence on the effectiveness, safety and limited data on women's experience and satisfaction. Our IPD meta‐analysis showed no reduction in caesarean delivery rate with outpatient balloon catheters and no change in the hospital stay. Using thoroughly cleaned data and adjusting for age and parity probably provided more accurate results in the present IPD meta‐analysis, which has similarly reported no differences. Even with the data from RCTs, adjusting for potentially confounding factors like age and parity would ensure more accurate effect estimates, as these factors are known to be associated with the outcomes of labour induction. In the present study, more use of oxytocin and epidural analgesia could be clinically correlated to each other as clinicians tend to recommend epidural analgesia with labour augmentation. The inpatient group had 285 women (three RCTs) started on oxytocin as part of their management [17, 51, 52], which is 11.0% of all women evaluated in the present study and unlikely to have made an impact. A significantly lower rate of fetal acidosis among the outpatient group is a reassuring finding because there was no formal fetal monitoring while they stayed outpatient. A large IPD meta‐analysis comparing balloon catheters with vaginal prostaglandins has shown less fetal acidosis, less hyperstimulation and more oxytocin augmentation with balloon catheters [4]. While in a recent IPD meta‐analysis by the authors, which compared outpatient balloon catheters with inpatient vaginal dinoprostone, it was found that outpatient balloon catheters led to fewer vaginal births, especially for women with overweight and obesity [60]. The perinatal and maternal safety profiles were comparable. However, as all three included studies were from Australia and New Zealand, generalizability could be low. The present study is the main study and is different from this recent publication [60], which addressed only the head‐to‐head comparison between inpatient vaginal dinoprostone vs. outpatient balloon catheters with three RCTs. In contrast, the current paper makes four comparisons with 11 RCTs. We have mentioned this intention prospectively in the study protocol (Appendix S3). The present study included all the available active comparisons for the outpatient vs. inpatient settings, focusing on the ‘sending outpatient’ intervention irrespective of the methods used for cervical ripening. Moreover, the present study also investigated the use of balloon catheters in both settings.

### 4.4 Clinical Implications

Our present IPD meta‐analysis has not included any RCTs with outpatient vaginal dinoprostone; only mechanical methods were included in the outpatient group. Available international‐wide clinical guidelines have added several recommendations for outpatient cervical ripening for IOL. WHO recommendations on outpatient settings for IOL have emphasised the low certainty of evidence for routine use and recommend careful patient selection based on transport resources and geographical location before offering this model of care; most existing data are from high‐income countries with limited statistical power for many important clinical outcomes [61]. WHO also mentions the role of shared decision‐making in balancing the existing evidence and potential risks and benefits [61].

The COVID‐19 pandemic has changed the way healthcare interventions are delivered [62]. Outpatient use of cervical ripening for IOL has recently re‐emerged with increasing popularity during the COVID‐19 pandemic, as it minimises hospital gathering with pandemic‐imposed restrictions on labour support persons and reduces virus transmission risks [63]. This approach allows women to initiate labour at home, aligning with public health measures and supporting women's preferences for a less medicalised birthing experience. The same can be considered for the post‐pandemic period. Furthermore, outpatient cervical ripening might support the current trend of climate‐resilient and environmentally sustainable healthcare models with regard to health workforce, cost and resources. It also has potential economic advantages for both the woman and the hospital, as well as for women's birth experiences. These are the current top considerations for any healthcare intervention. Robust evidence from diverse settings in this regard is a dire need for this common day‐to‐day obstetric intervention. All the existing RCTs have been performed in high‐ or upper‐middle‐income countries. The impact of this intervention on low‐ and low‐middle‐income countries has yet to be explored. Potential logistic factors like transport difficulties and higher background morbidity and mortality rates are possible challenges for these countries to implement outpatient cervical ripening before IOL.

## 5 Conclusion

The overall effectiveness and perinatal and maternal safety of outpatient cervical ripening before IOL are comparable to those of inpatient cervical ripening. Cervical ripening in an outpatient setting leads to less fetal acidosis, more maternal epidural analgesia, and more oxytocin use than inpatient.

## Author Contributions

M.P. principally managed the project and the collaborative process and carried out design, data collection, IPD checking, data synthesis, writing, editing, finalising, submitting and revising of the manuscript. B.W.M. and W.L. conceived the research idea and were responsible for overseeing all aspects of conduct. As the second and third reviewers, F.C. and W.L. contributed to eligibility screening and risk of bias assessment. D.L.R. and B.W.M. provided clinical oversight. M.P. designed and conducted the literature searches. All authors were involved in the decision to submit the manuscript. All contributing trial investigators had opportunities to comment on the initial scope, draft protocol, and draft statistical analysis plan and participated in teleconference meetings as the project progressed. Trial investigators also prepared and supplied data and answered questions about their RCTs.

## Ethics Statement

Ethics approval was obtained from the Monash Health Human Research Ethics Committee on 27 April 2022 (RES‐22‐0000‐119Q‐84527).

## Conflicts of Interest

M.P. is supported by a Research Training Stipend, provided by the Australian Government. B.W.M. declared grants from NHMRC, personal fees from ObsEva, personal fees from Merck, personal fees from Guerbet, others from Guerbet, and grants from Merck, outside the submitted work. D.L.R. has received fees to participate in Advisory boards from Alexion, travel support, and lecture fees from the International Society of Ultrasound in Obstetrics and Gynaecology (ISUOG), unrelated to this work. W.L. declared a grant from NHMRC that supports this work and received research grant funds from the Norman Beischer Medical Research Foundation, which were unrelated to this work. The protocol, statistical analysis plan, and codebook are available on request. The trial investigators who shared individual participant data for the purposes of the meta‐analysis retain ownership of their trial data, and any requests for access to individual participant data should be made directly to them.

## Supporting information


**Appendix S1.** Pre‐defined statistical analysis plan.

**Appendix S2.** Pre‐defined IPD meta‐analysis codebook.

**Appendix S3.** Pre‐defined study protocol.

Data S1.

## Data Availability

The protocol, statistical analysis plan and codebook are available on request. The trial investigators who shared individual participant data for the purposes of the meta‐analysis retain ownership of their trial data and any requests for access to individual participant data should be made directly to them.
