# Supplementary material for: Outpatient or Inpatient Setting for Cervical Ripening Before Induction of Labour: An Individual Participant Data Meta‐Analysis
Source: BJOG. 2025 Jun 11;132(13):1966–82. doi: 10.1111/1471-0528.18253 (PMC12592782; doi:10.1111/1471-0528.18253)
Supplement: Supplementary file 1 — Appendix S1. Pre‐defined statistical analysis plan. [file BJO-132-1966-s002.pdf]

Statistical Analysis Plan IPD- Outpatient versus inpatient settings for induction of labour

Title

Outpatient versus inpatient settings for induction of labour: individual participant data meta-analysis.

Members

M Patabendige, D.L. Rolnik, B.W. Mol, W. Li

Department of Obstetrics and Gynaecology, School of Clinical Sciences at Monash Health, Monash University, Clayton, VIC, Australia.

Contents

Statistical Analysis Plan IPD- vaginal dinoprostone versus vaginal misoprostol for induction of labour 1
Introduction 3
Rationale 3
Design and setting 3
Objectives 4
Methods 5
Search strategy and eligibility criteria 5
Data collection 6
Software 7
STATISTICAL ANALYSIS I: OVERVIEW ..... **Error! Bookmark not defined.**
Risk of bias, quality appraisal and data checking 7
Overall approach to IPD meta-analysis 9
Type of analysis 9

Protocol violations, withdrawals, missing data, outliers and unexpected irregularities 9
Baseline characteristics 12
Treatment outcomes 14
Effect modifiers (treatment-covariate interaction) 21
STATISTICAL ANALYSIS II: META-ANALYSIS 23
STATISTICAL ANALYSIS III: SUBGROUP ANALYSIS 24
STATISTICAL ANALYSIS III: SENSITIVITY ANALYSIS 24

PROTOCOL SIGNATURE SHEET

**Name**	**Signature**
Investigator<br>Malitha Patabendige	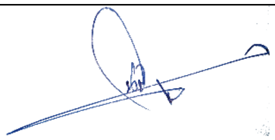
Investigator<br>Wentao Li	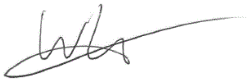
Investigator<br>Daniel L. Rolnik	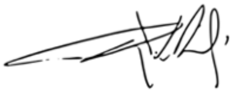
Principal investigator<br>Ben W. Mol	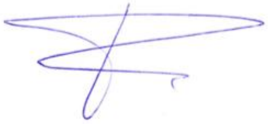

Introduction

Rationale

Induction of labour (IOL) is one of the most commonly performed obstetric procedures worldwide. It is performed when the artificial initiation of labour is deemed necessary compared to the expectant management for the well-being of both the mother and the newborn. Currently, IOL is carried out after admission to the hospital in most settings. Outpatient cervical ripening and IOL are attractive options that include both mechanical and pharmacological methods. Theoretically, outpatient cervical ripening and IOL might yield several potential advantages, such as a reduction in the length of hospital stay, higher maternal satisfaction, and a reduction in cost. However, limited data on its effectiveness and safety with a highly variable consumer uptake worldwide was available. The individual participant data meta-analysis (IPDMA) is carried out by extracting original research data from the individual researchers via direct contact (1). While the traditional systematic reviews are performed using the data extracted from the published studies along with methodological limitations of aggregate data, IPDMA generates a single central database to be analysed and combined, if appropriate, as a meta-analysis (2). For this reason, IPDMA is known as the 'gold standard of systematic reviews, yielding a more substantial amount of quality data to guide the clinical practice (1, 2).

The recent aggregate data meta-analysis comparing outpatient and inpatient cervical ripening with balloon catheters by Pierce-Williams et al. has provided some positive insights (3). However, before drawing into conclusions to change the clinical practice based on an aggregate data meta-analysis, performing an IPDMA is worthwhile as IPDMA carries better trustworthiness and wider analysis using raw data. The existing meta-analysis by Pierce-Williams et al. has not adequately assessed safety outcomes that usually lack power for analysis. This issue might be addressed in an IPDMA using raw data as composite safety outcomes could be constructed. There are a few trials comparing prostaglandins for both outpatient and inpatient arms. Cochrane review on the 'Home versus inpatient induction of labour for improving birth outcomes' has summarised randomised controlled trials studying prostaglandins and balloon catheters up to 2020 (4). In overall, they have

concluded the limited data available from relatively small trials. Similarly, a systematic review and meta-analysis by Dong et al. in 2020 summarised all the available trials for the same duration, including prostaglandins, balloon catheters, outpatient amniotomy and oxytocin infusion concerning the overall summary statistic. Evidence on the effectiveness and safety is still uncertain. All of these are aggregate data meta-analyses; hence, the value of individual participant data meta-analysis would be able to understand the exact situation of the strength of the evidence. Therefore, we have planned an IPDMA to compare the effectiveness and safety of the outpatient versus inpatient methods in IOL with different methods.

This is an IPDMA of randomised control trials (RCTs) that compare the effectiveness and safety of the outpatient versus inpatient methods in IOL with different methods.

Objectives

1. To compare the overall effectiveness and safety of induction of labour in the outpatient setting versus the inpatient setting, irrespective of the methods used.
2. To compare the effectiveness and safety of induction of labour in the outpatient setting versus the inpatient setting when balloon catheters are used for both settings.
3. To compare the effectiveness and safety of induction of labour in the outpatient setting using balloon catheters versus the inpatient setting when vaginal dinoprostone is used.
4. To compare the effectiveness and safety of induction of labour in the outpatient setting versus inpatient setting when vaginal prostaglandins are used in both settings.
5. To compare the effectiveness and safety of induction of labour in the outpatient setting versus the inpatient setting when any other method was used in one or both settings, i.e., osmotic dilators, dinoprostone, and misoprostol.
6. To compare the overall effectiveness and safety of induction of labour in the outpatient setting versus the inpatient setting when mechanical methods are used in both settings.
7. To study differences in the effectiveness of induction of labour in the outpatient setting versus the inpatient setting with:
    A. Nulliparous or multiparous

B. Various maternal age

C. Various maternal BMI

D. Various initial Bishop score

E. Different indications for induction of labour

Methods

Search strategy and eligibility criteria

Randomised controlled trials with outpatient and inpatient cervical ripening and/or induction of labour for women with unfavourable cervices were included. All pharmacological and mechanical methods that are currently being used in outpatient and inpatient settings were included, irrespective of the gestational age. Membrane sweeping, nipple stimulation, or other non-pharmacologic and non-mechanical methods not in line with contemporary obstetrical practice were excluded. In other words, only the currently recommended labour induction methods per major clinical guidelines (NICE, ACOG and RANZCOG) were included. In addition, trials with only an active intervention in both arms were included, and therefore, trials with the placebo effect, expectant management, attending physician management, etc., were excluded. Pregnant women with unfavourable cervices and singleton fetuses at or beyond 34 weeks of gestation who have an indication for induction of labour were included. Women were included irrespective of membrane status, previous caesarean or scar pregnancy. Cluster randomised trials, cross-over and quasi-experimental trials were excluded.

Potentially suitable clinical trials were identified from inception to August 2022, and the search was updated in September 2022 and again in January 2024. Databases including Ovid MEDLINE, Ovid Embase, Ovid Emcare, CINAHL Plus, Scopus, Cochrane Pregnancy and Childbirth Group's Trials Register, the WHO International Clinical Trials Registry Platform (ICTRP) and clinicaltrials.gov (for unpublished, planned and ongoing trial reports). Reference lists of all included studies, and previously conducted systematic reviews were searched for articles that might have been missed through the formal search (citation-tracking). Cochrane review on the

‘Home versus inpatient induction of labour for improving birth outcomes’ has summarised randomised controlled trials studying prostaglandins and balloon catheters up to 2020 and another systematic review and meta-analysis by Pierce et al. compared trials where balloon catheters used for both settings (3, 4). These two meta-analyses were also a guide to finding studies. There were no language barriers, and all the published and unpublished data were eligible.

Two investigators independently reviewed the identified papers for eligibility, with disagreements solved by a third reviewer. The principal authors of eligible trials were subsequently invited to contribute their raw data for analysis.

Population

Pregnant women in a third trimester with an unfavourable cervix who require induction of labour.

Intervention/treatment

The intervention measured in each study was an outpatient setting (sending the woman home) after starting the process of cervical ripening/induction of labour.

Comparator/control

The comparator was the use of an inpatient setting for the induction of labour.

Outcomes

Studies were eligible if they reported the rate of vaginal delivery for each group.

Data collection

The principal investigators of all eligible trials identified were contacted via email to participate in this study. If there was no response for two weeks, another gentle reminder was emailed. They were asked to share the individual patient data collected from their trials. If they failed to respond, the co-authors, last authors, institute and journal editors were contacted via email. The principal investigators were asked to complete an Excel datasheet or send their data with a document clearly stating the definitions used for variables. The collected data should contain baseline patient

characteristics including age, gestational age, parity, ethnicity, body mass index (BMI) as pre-pregnancy and/or at the time of randomisation, smoking status, and the modified Bishop score before intervention. Coding of the data guaranteed that the key to linking IPD to patient identity remained in the possession of each research group. For those studies for which IPD cannot be obtained, we sought to collect the reasons for the non-availability of IPD.

Software

SPSS and R will be used for all statistical analyses.

STATISTICAL ANALYSIS I: META-ANALYSIS

Flow chart

We will construct the PRISMA-IPD flow diagram(5) detailing the process of study selection and collection of IPD and aggregate data. This diagram will report the number of studies identified, screened for eligibility, and included or excluded, together with the number of participants in these studies and the reasons for exclusion at each stage. The flow diagram will also report the number of studies that provided IPD and those that did not, along with the reasons why IPD could not be obtained in the latter case and the number of participants for which IPD could or could not be obtained.

Risk of bias, quality appraisal and data checking

- Risk of bias

Two review authors will independently assess study quality and risk of bias. If there are disagreements, a third author will be consulted. The risk of bias will be assessed using Version 2

of the Cochrane risk-of-bias tool for randomised trials (RoB 2) (6). Additionally, we will test for data availability bias by constructing the summary effect estimates with or without aggregate data of RCTs not providing IPD.

- Quality of the data, Integrity Check and trustworthiness assessment

Data will be checked for missing values and entries that are outside the expected ranges, and internal consistency checks will be performed. The pattern of missing data will be analysed separately for each dataset using the patterns chart. All variables besides the ID variable will be selected. We will analyse variables that are missing >0.01% of data. First, we will check which variables have missing data. Then, using the missing value pattern chart, we will look for monotonicity. If missing data is concentrated in the upper left and lower right corner, there is a systematic pattern in missing data. If there are only patches or islands of missing values, the data is likely missing at a random pattern.

The quality of the data will be assessed by comparing the provided IPD with the published baseline data and results. The baseline table will be reconstructed for each study. The outcome data will be analysed, and the results will be compared with the published analyses. In case of any discrepancies or queries which may arise, the principal investigator will be contacted, and corrections will be made if necessary. Key design features and demographic characteristics of the included trials will be summarised in a table.

We will also check the trustworthiness concerns on the studies using the Trustworthiness in RAndomised Controlled Trials (TRACT) trustworthiness checklist (11). This screening tool aims to help identify and triage studies at risk of integrity issues. The checklist includes seven domains that apply to every study: governance, author group, plausibility of intervention usage, timeframe, drop-out rates, baseline characteristics and outcomes. Two review authors will perform the assessment for all the studies published in full-text form and disagreements will be resolved by consensus. We adopted a scoring system for this TRACT checklist ('*No Concerns*' - 0 score, '*Some Concerns/No Information*' - 1 score, '*Major concerns'* - 2 scores) to make it easier for the

categorisation. The categorisation of *'not meeting our trustworthiness criteria'* and '*meeting our trustworthiness criteria'* for studies will be based on the TRACT score, published year, critical items on the TRACT checklist and the overall quality of the paper.

Overall approach to IPD meta-analysis

Our primary analysis will be a 'two-stage' meta-analysis method to synthesise the IPD. This will be conducted to obtain forest plots of the effect estimates and a measure of between-study heterogeneity. However, for outcomes that had zero events in any intervention group in any included trial, a 'one-stage' method will be used. A 'one-stage' meta-analysis will also be performed for primary outcomes as a sensitivity analysis strategy.

Type of analysis

Primarily, an intention to treat analysis will be performed. An as treated (AT) analysis will be carried out as part of a sensitivity analysis strategy.

- Intention to treat (ITT): All participants will be included in the analysis and will be evaluated according to the treatment to which they were randomised.

- As treated (AT): All participants will be analyzed according to the treatment they actually received. If participants became lost to follow-up or withdrew their informed consent before the intervention could have taken place, they will be excluded from all analyses.

Protocol violations, withdrawals, missing data, outliers and unexpected irregularities

<u>Criteria</u>

- **Protocol violations:**

- ✓ Wrongfully included patients will be excluded from the full analysis set.
- ✓ Cross-over: patients receiving the treatment of the arm to which they were not randomised will primarily be analysed according to the treatment to which they were allocated (i.e. intention to treat analysis). In the secondary as treated analysis, these patients will be analysed in the arm corresponding to the treatment they actually received.

➢ **Withdrawals:**

- ✓ Withdrawals will be included in the data analysis up to when the participant withdrew informed consent.
- ✓ Missing covariate data after withdrawal of informed consent will be marked as 'missing' and imputed.
- ✓ Participants with missing outcome data after withdrawal of informed consent will be excluded from analysis for the missing outcome(s).

➢ **Lost to follow-up:**

- ✓ Data will be included until the time at which the participant became 'lost to follow up' during the follow-up period of the study.
- ✓ Missing covariate data for participants who became lost to follow-up will be marked as 'missing' and imputed.
- ✓ Participants with missing outcome data after loss to follow-up will be excluded from analysis for the missing outcome(s).

➢ **Missing data:**

- ✓ No available data at all for a participant: exclusion from the full analysis set.
- ✓ Missing outcome data: exclusion from analysis for the missing outcome(s).
- ✓ Sporadically missing covariate data: multiple imputation.
- ✓ Entire variables missing from one study (systematically missing data): no imputation.
- ✓ For further details on imputation see <u>Methods for imputation of missing data</u> below.

➢ **Unexpected irregularities:**

- ✓ The strategy will be decided on when an unexpected irregularity is found in the dataset. This will be clearly described in the final manuscript.

- ➢ **Outliers:**
- ✓ If outliers cannot be resolved, they will be marked as 'missing' and imputed.

The abovementioned is summarized in the following table:

**Category**	**Problem**	**Action**
Protocol violation	Wrongfully included patients	Exclude from all analyses
	Cross-over	ITT analysis: analyze according to allocation<br>AT analysis: analyze according to received<br>treatment
Withdrawals<br>***And loss to follow-up***	Before the time point for which intervention<br>was scheduled	Exclude from all analyses
	After the time point for which intervention was scheduled	Include in all analyses of outcomes for which outcome data is available
Missing data	No available data at all	Exclude
	Sporadically/randomly missing data	Determine likely missing pattern<br>Multiple imputation within studies if MAR
	Systematically missing data	No imputation

Unexpected irregularities	Not yet known	To be determined on a case-by-case basis
Outliers	Outliers will primarily be tried to resolve. It may sometimes be impossible to resolve.	Mark as missing and impute

Criteria for handling protocol violations, missing data, outliers and unexpected irregularities in the datasets. Abbreviations: MAR = missing at random

<u>Methods for imputation of missing data</u>

- Only missing covariate data will be imputed with multiple imputation. No outcome will be imputed.
- Sporadically missing data will be imputed *within each study* by means of chained equations (10 datasets).
- Multiple imputation techniques assume MAR (missing at random) pattern. If data is likely to be MNAR (missing not at random) or MCAR (missing completely at random), we will conduct a sensitivity analysis in order to assess the robustness of the imputed datasets under different hypotheses for the missingness pattern.

Baseline characteristics

<u>Methods</u>

- Baseline tables will be constructed separately for each study and will display only the baseline characteristics of those participants included in the analysis.
- In all cases where we aim to report a mean (±SD), a median with interquartile ranges will be given instead of mean with standard deviation if the variable is non-normally distributed. Binary and categorical variables will be presented as the number of total and percentage.

- No statistical test will be performed to determine if the groups are significantly different from each other or not, as we assume any baseline differences after randomization have occurred due to chance.
- Overall baseline characteristics will be presented per group.

Missing values
- The number of missing values will be indicated in the table.

Variables

- The following variables will be reported:

1. Maternal age in years
2. Maternal Body Mass Index in kg/m2
3. Female parity, n (%)

- 0
- 1
- ≥2

4. Indication for induction of labour

- Hypertensive disorders, n (%)
- Post-dates gestation, n (%)
- Diabetes/gestational diabetes, n (%)
- Intrauterine growth restriction/fetal growth restriction, n (%)
- Oligohydramnios, n (%)
- Advanced maternal age, n (%)
- Elective, n (%)
- More than one indication, n (%)
- Other/unknown, n (%)

5. Initial Bishop score

Treatment outcomes

We will study the effect of treatment for the following outcomes:

<u>Primary:</u>

1. Vaginal delivery rate

2. Composite of adverse perinatal outcome: a composite measure of stillbirth, neonatal death, neonatal Apgar score <7 at 5 minutes, acidosis (pH<7.1), neonatal seizures, hypoxic ischaemic encephalopathy of any stage (HIE), neonatal intensive care unit (NICU) admission for any duration, meconium aspiration syndrome, neonatal infection either clinically suspected (as defined by neonatal antibiotic administration) or proven neonatal infection (culture proven), cord prolapse, endotracheal intubation and/or external cardiac compressions.

3. Composite of adverse maternal outcome: a composite measure of admission to ICU for any period, maternal infection (defined as a temperature $\geq 38°C$ at any time during labour or delivery or antibiotic use or clinically diagnosed infection, such as endometritis), postpartum haemorrhage ≥1000 mL, maternal death, uterine rupture (either full or partial thickness damage as defined by the trialists).

<u>Secondary:</u>

1. Delivery outcomes:

- Mode of delivery (spontaneous vaginal birth, instrumental vaginal birth and caesarean section)

- Caesarean section indication (i.e., fetal compromise or failure to progress. If both fetal compromise and failure to progress apply as indications, fetal compromise will prevail.

- Instrumental vaginal birth and indication for instrumental vaginal birth- fetal compromise or failure to progress. If both fetal compromise and failure to progress apply as indications, fetal compromise will prevail.

- Time from commencement of labour induction to vaginal delivery (cumulative rate of vaginal birth)
- Time from admission to hospital to delivery

2. Labour progression outcomes:
- Change in modified Bishop Score
- Uterine tachysystole, hypertonus
- Uterine hyperstimulation
- Oxytocin augmentation
- Meconium-stained amniotic fluid
- Unsuccessful induction (defined as a change in induction method from the originally allocated method)
- Total days of hospital stay for mother and neonate
- Use of epidural analgesia and opioid analgesia during labour

3. Maternal safety outcomes:
- Individual components of the primary composite maternal outcome

4. Neonatal safety outcomes
    - Individual components of the primary composite perinatal outcome

5. Satisfaction
    - Maternal satisfaction

Additionally, other parameters such as neonatal birth trauma (mechanical causes during childbirth), bowel obstruction, thrombo-embolic events, stroke, hypertensive disorders, hysterectomy for any complications resulting from birth, Damage to internal organs (bladder,

bowel, ureters), cardio-respiratory arrest and other maternal postpartum conditions requiring treatment will be assessed. However, these will be analysed depending on the availability of adequate data.

The way outcomes are recorded, and their corresponding variables and possible values are summarized in the following table:
FHR: Fetal heart rate; MOD: Mode of delivery.

**Primary and secondary delivery outcomes**	mod<br>(mode of delivery- MOD)	Categorical<br>2 = instrumental<br>1 = caesarean section<br>0 = vaginal unassisted
	instru-ind<br>(indication for vacuum or forceps extraction)	Categorical<br>0 = not applicable- other MOD<br>1 = failure to progress (FTP) in the second stage<br>2 = fetal distress<br>3 = Fetal distress +FTP both<br>4= poor maternal effort<br>5= maternal medical complications<br>6= other
	Instru_ftp<br>Instrumental delivery for failure to progress	Categorical<br>0 = no, 1 = yes
	Instru_fhr<br>instrumental delivery for abnormal FHR	Categorical<br>0 = no, 1 = yes
	cs-ind<br>(indication for caesarean section)	Categorical<br>0 = not applicable: other MOD<br>1 = failure to progress (FTP)

		2 = fetal distress<br>3 = failed induction<br>4 = failed instrumental<br>5 = fetal distress + FTP<br>6=maternal complication-specify)<br>7= other<br>8=cord prolapse<br>9=abruption<br>10= fetal distress + FTP
	Cs_ftp_new<br>caesarean section for failure to progress	Categorical<br>0 = no, 1 = yes
	cs_abn_fhr<br>caesarean section for abnormal FHR	Categorical<br>0 = no, 1 = yes
	mech-dura<br>(duration of mechanical method in-situ in hours)	Continuous
	More_methods<br>(use of more than one method)	Categorical<br>0 = no, 1 = yes
	Failed_ind<br>failed_induction (use of other methods for IOL after originally allocated method)	Categorical<br>0 = no, 1 = yes
	Other_methods<br>(other methods for IOL after the originally allocated method)	Categorical<br>0= no other methods<br>1 = balloon catheter<br>2 = Amniotomy<br>3 = Oxytocin<br>4 = Dilapan-S<br>5 = Amniotomy and oxytocin both<br>(please combine if necessary like in 9)

	Idi_hr (induction to delivery interval in hours)	Continuous
	Idi_min (induction to delivery interval in min)	Continuous
	adm_deli_inte_hr (admission to delivery interval in min)	Continuous
	Total maternal stay in hours total_hos_stay_mat	Continuous
	Total neonatal stay in hours total_hos_stay_neo	Continuous
	bs_max (maximum Bishop score recorded/post-intervention)	Continuous
**Secondary labour progression outcomes**	Ut_tachy (uterine tachysystole)	Categorical 0 = no, 1 = yes
	Ut_hypersti (uterine hyperstimulation)	Categorical 0 = no, 1 = yes
	ut_ hypertonus hypertonus	Categorical 0 = no, 1 = yes
	Oxy_aug (use of oxytocin during labour)	Categorical 0 = no, 1 = yes
	Mec_amnio (meconium stained amniotic fluid)	Categorical 0 = no, 1 = yes
	analge (analgesia during labour)	Categorical 0 = no, 1 = yes

	analge_cat<br>(analgesia during labour)	Categorial<br>0 = None<br>1= Epidural use<br>2 = Parental opioids<br>3 = Entonox<br>4 = Other, specify
	epidural<br>Epidural use	Categorical<br>0 = no, 1 = yes
**Secondary maternal morbidity outcomes**		
	M_anbx (maternal antibiotics given)	Categorical<br>0 = no, 1 = yes
	M_fever (maternal temperature ≥38C)	Categorical<br>0 = no, 1 = yes
	m_infec<br>(suspected/proven maternal infection)<br><br>m_infec_total = composite of fever, infection all to be used for maternal composite	Categorical<br>0 = no, 1 = yes
	Endomet<br>(endometritis suspected or proven)	Categorical<br>0 = no, 1 = yes
	ebl<br>(estimated blood loss in mL)	Continuous
	pph-1000<br>(severe post-partum haemorrhage 1000mL or more)	Categorical<br>0 = no, 1 = yes
	Pph500<br>(post-partum haemorrhage 500ml or more)	Categorical<br>0 = no, 1 = yes
	uti<br>(urinary tract infection)	Categorical<br>0 = no, 1 = yes
	bowel<br>(bowel obstruction)	Categorical<br>0 = no, 1 = yes
	vte	Categorical

	(thromboembolic event)	0 = no, 1 = yes
	hyperten<br>(hypertensive disorder in pregnancy)	Categorical<br>0 = no, 1 = yes
	pet<br>(pre-eclampsia/HELLP)	Categorical<br>0 = no, 1 = yes
	icu<br>(maternal ICU admission)	Categorical<br>0 = no, 1 = yes
	Ut_rupture<br>(uterine rupture)	Categorical<br>0 = no, 1 = yes
	M_death<br>(maternal death)	Categorical<br>0 = no, 1 = yes
	oth-cx<br>(other postpartum condition requiring hospital admission)	Categorical<br>0 = no, 1 = yes
**Secondary neonatal morbidity outcomes**	stillbirth	Categorical<br>0 = no, 1 = yes
	nnd	Categorical<br>0 = no, 1 = yes
	Apgar<br>APGAR score recorded	Continuous
	Apgar_cat<br>(APGAR score recorded as <7 at 5 minutes)	Categorical<br>0 = no (Good APGAR 7-10 at 5 min)<br>1 = yes (Bad APGAR 0-6 at 5 min)
	fever_neonatal (neonatal temperature ≥38C)	Categorical<br>0 = no, 1 = yes
	Ph_cat<br>(arterial umbilical cord pH <7.10)	Categorical<br>0 = no (pH 7.10 or greater)<br>1 = yes (pH <7.09 or less)
	ph	Continuous
	lactate	Categorical<br>0 = no (<4.79)<br>1 = yes (4.8 or greater)
	nicu	Categorical<br>0 = no, 1 = yes

	(admission to neonatal intensive care unit for any duration)	
	seizures<br>(neonatal seizures)	Categorical<br>0 = no, 1 = yes
	Mech_venti<br>(mechanical ventilation)	Categorical<br>0 = no, 1 = yes
	Neo_infec<br>(suspected/proven neonatal infection)	Categorical<br>0 = no, 1 = yes
	Neo_anbx<br>(neonatal antibiotics given)	Categorical<br>0 = no, 1 = yes
	Meconium_syndrome<br>(meconium aspiration syndrome)	Categorical<br>0 = no, 1 = yes
	hie<br>Hypoxic ischaemic encephalopathy	Categorical<br>0 = no, 1 = yes
	respi_dis<br>Severe neonatal respiratory compromise	Categorical<br>0 = no, 1 = yes
	Cord<br>Cord Prolapse	Categorical<br>0 = no, 1 = yes
	Ecc<br>External cardiac compression	Categorical<br>0 = no, 1 = yes

<u>Effect modifiers (treatment-covariate interaction)</u>

Effect modifiers will be studied on the ITT population only. Only within-study interactions will be considered. We will study the following hypothesized individual-level modifiers of treatment outcome in terms of caesarean section:

- Nulliparous or multiparous women: Parity will be recorded as such for each woman and will be analysed as a binary variable.
- Maternal age: In years with one decimal and will be analysed as a continuous variable.
- Gestational age: In weeks as integer values and will be analysed as a continuous variable.
- Maternal BMI: In kg/m2 with one decimal and will be analysed as a continuous variable.
- Initial Bishop score: Integer values and will be analysed as a continuous variable.
- Indications for IOL (if data are available enough):

Interested indications include hypertensive disorders, post-date gestation, diabetes/gestational diabetes, intrauterine growth restriction/ fetal growth restriction, and advanced maternal age. All indications will be compared to elective IOL (reference) as a categorical variable.

These interactions and their corresponding variables and possible values are summarized in the following table:

Variable	Type	Values	Description
parcode	Binary	0 = "Nulliparous"<br>1 = "Multiparous"	Nulliparous, parity=0<br>Multiparous, parity>=1
age	Continuous	One decimal	Maternal age in years
gestage	Continuous	Integer	Gestational age in weeks
bmi	Continuous	One decimal	Maternal BMI in kg/m2
bs_begi	Continuous	Integer	Bishop score at baseline
iol-ind	Categorical	0 = no, 1 = yes for the following indications separately.<br><br>Post-dates gestation<br>Hypertensive disorders<br>Diabetes/gestational diabetes<br>IUGR/fetal growth restriction<br>Advanced maternal age<br>Obstetric cholestasis<br>Oligohydramnios<br>Other<br>Elective/social	Indications for induction of labour. Other indications will not be considered.

Hypothesized interactions (effect modifiers, subgroups) that will be studied.

STATISTICAL ANALYSIS II: META-ANALYSIS

<u>Two-stage method</u>

Step 1

For each included study, the outcomes will be compared between the treatment (outpatient group) and control (inpatient group) groups. All models will adjust for maternal age and parity. For binary outcomes, odds ratios with 95% confidence intervals will be calculated using logistic regression. For time to vaginal delivery (induction to vaginal delivery interval), we will estimate the sub-distribution hazard ratio (HRs) and 95% CIs using the sub-distribution hazard competing risk model, which considers caesarean section as a competing risk. Total days of hospital stay for the mother and neonate will be analysed using negative binomial regression separately.

For adverse outcomes, an OR >1.0 indicates that the outpatient group may confer a greater risk, whereas, for positive outcomes (for example, vaginal birth), an SHR or OR >1.0 indicates that the outpatient group may be more favourable. Effects estimates of the above-mentioned within-study interactions will be computed for primary outcomes.

Step 2

The generated summary estimates of studies will be combined using a random-effects model, as we assume differences in treatment effect due to between-study heterogeneity.

- Heterogeneity variance τ2 will be estimated with the method of restricted maximum likelihood (REML) estimator with Hartung-Knapp-Sidik-Jonkman variance correction.
- The I_2 statistic will be calculated to provide an additional measure of between-study heterogeneity.
- 95% confidence intervals will be calculated.
- We will calculate 95% prediction intervals.

- The results will be presented in forest plots: Labour and delivery outcomes, composite maternal and neonatal outcomes will be presented in forest plots separately.

One-stage method

One-stage method will be the main analysis for outcomes with zero event in any group in any study (e.g., postpartum haemorrhage > 1L, uterine hyperstimulation, tachysystole, Apgar score <7 at 5 min, arterial umbilical cord pH <7·1 and admission to neonatal intensive care unit). For these outcomes, random effects logistic regression will be used. The models will have a stratified intercept and a random treatment effect, covariates including age and parity will have fixed effects. Study-specific centering will be applied to control downward bias.

STATISTICAL ANALYSIS III: SUBGROUP ANALYSIS

Subgroup effects for the primary outcomes will be assessed using interaction terms between treatment and baseline covariates. Interactions will be computed within each study and then pooled using random-effects models. This is recommended to avoid ecological bias (aggregation bias). Subgroup effects for parity (nulliparous versus multiparous), maternal age, body mass index, and gestational age at induction (as a continuous and grand-mean centered variable) will be explored.

STATISTICAL ANALYSIS III: SENSITIVITY ANALYSIS

1. 'One-stage' analysis for primary outcomes

Sensitivity analysis for primary outcomes using a one-stage method will be produced to see any difference between two-stage methods. We will use random effects logistic regression to allow for heterogeneity across trials. The models will have a stratified intercept and a random treatment effect. Study-specific centering and adjusting for maternal age and parity will be applied to control for downward bias.

2. IPD unavailability bias

We will extract aggregate data on vaginal birth from publications of all trials irrespective of data sharing status and combine these estimates in a two-stage meta-analysis. This aggregate data meta-analysis using the random-effects model (REML with H-K-S-J variance correction) will be compared to the results of the IPD meta-analysis. This method will not be possible for the two composite outcomes of maternal and neonatal safety. We will also do aggregate data meta-analysis for secondary outcomes.

3. Publication bias

Funnel plots will be created for all eligible RCTs to see whether all RCTs fall within the expected 95% range, giving a symmetric view.

4. As-treated (AT) analysis

AT analysis for primary outcomes will be done to investigate the effect of the meta-analytical method on the pooled estimates compared to the intention-to-treat analysis.

References

1. Tierney JF, Vale C, Riley R, Smith CT, Stewart L, Clarke M, et al. Individual Participant Data (IPD) Meta-analyses of Randomised Controlled Trials: Guidance on Their Use. PLoS Med. 2015;12(7):e1001855.
2. Ventresca M, Schünemann HJ, Macbeth F, Clarke M, Thabane L, Griffiths G, et al. Obtaining and managing data sets for individual participant data meta-analysis: scoping review and practical guide. BMC Med Res Methodol. 2020;20(1):113.
3. Pierce-Williams R, Lesser H, Saccone G, Harper L, Chen V, Sciscione A, et al. Outpatient Cervical Ripening with Balloon Catheters: A Systematic Review and Meta-analysis. Obstet Gynecol. 2022;139(2):255-68.
4. Alfirevic Z, Gyte GM, Nogueira Pileggi V, Plachcinski R, Osoti AO, Finucane EM. Home versus inpatient induction of labour for improving birth outcomes. Cochrane Database Syst Rev. 2020;8:CD007372.
5. Stewart LAea. 'Preferred Reporting Items for Systematic Review and Meta-Analyses of individual participant data: the PRISMA-IPD Statement.'. JAMA. 2015;313(16), pp. 1657–65.
6. Sterne JACea. 'RoB 2: A revised tool for assessing risk of bias in randomised trials'. The BMJ. 2019(366):1-8.
