# Supplementary material for: Outpatient or Inpatient Setting for Cervical Ripening Before Induction of Labour: An Individual Participant Data Meta‐Analysis
Source: BJOG. 2025 Jun 11;132(13):1966–82. doi: 10.1111/1471-0528.18253 (PMC12592782; doi:10.1111/1471-0528.18253)
Supplement: Supplementary file 2 — Appendix S2. Pre‐defined IPD meta‐analysis codebook. [file BJO-132-1966-s003.pdf]

IPD-MA data dictionary for *"Outpatient versus inpatient methods for induction of labour: individual participant data meta-analysis"*

**Identification**	sid<br>(Study ID)	Continuous
	oldpid<br>(old participant ID)	Continuous<br>Study ID used in the original study
	Author, published year	Wise 2023 etc
**Induction modality**	Induction modality1; im1<br>(randomised group)<br>Ind_cat	Categorical<br>0 = inpatient<br>1 = outpatient
	Induction modality2; im2<br>(randomised group)<br>ind_cat_subgroup	Categorical<br>0 = outpatient balloon catheter + inpatient balloon catheter<br>1 = outpatient balloon catheter + inpatient PGE2<br>2 = outpatient Dilapan + inpatient Dilapan<br>3 = outpatient balloon catheter + inpatient balloon catheter and oxytocin
	Induction modality3; im3<br>Actually received method of labour induction<br>(As-treated group-<br>trt_received) "as definition"	Categorical<br>0 = inpatient<br>1 = outpatient
	Actually received method of labour induction<br>(As-treated group-<br>trt_received_subgroup)	Categorical<br>0 = Dinoprostone/PGE2<br>1 = Balloon<br>2 = Dilapan<br>3 = Misoprostol
**Baseline variables**	age	Continuous
	Bmi kg/m2 at induction	Continuous
	Weight /kg	Continuous
	Height/cm	Continuous
	BMI at booking kg/m2	Continuous
	gestage<br>(gestational age in weeks)	Continuous
	Ethnicity	Categorical<br>0 = Caucasian<br>1 = Hispanic<br>2 = Asian<br>3 = Black<br>4 = other (specify)

	smoking<br>(Smoking status)	Categorical<br>0 = no, 1 = yes
	parity	Continuous
	parcode<br>(parity code)	Categorical<br>0 = nulliparous<br>1 = multiparous
	Ind_iol (indication for IOL)	
	Categorical<br>0 = other/unknown<br>1 = hypertensive disorders<br>2 = postdates<br>3 = diabetes/gestational diabetes<br>4 = IUGR/fetal growth restriction<br>5 = oligohydramnios<br>6 = advanced maternal age<br>7 = obstetric cholestasis<br>8 = elective/social<br>9 = >1 indication	Categorical<br>0 = no, 1 = yes
**Bishop score**	bs_begi<br>(initial Bishop score before intervention)	Continuous
**Singleton**	Singleton	Categorical<br>0 = singleton, 1 = multiple
**Membrane status (membrane_status)**	srom<br>Membrane status<br>Categorical	1= intact<br>0= ruptured
**Primary and secondary delivery outcomes**	mod<br>(mode of delivery- MOD)	Categorical<br>2 = instrumental<br>1 = caesarean section<br>0 = vaginal unassisted
	instru-ind<br>(indication for vacuum or forceps extraction)	Categorical<br>0 = not applicable- other MOD<br>1 = failure to progress (FTP) in the second stage<br>2 = fetal distress<br>3 = Fetal distress + FTP both<br>4= poor maternal effort<br>5= maternal medical complications<br>6= other
	Instru_ftp	Categorical<br>0 = no, 1 = yes

	Instrumental delivery for failure to progress	
	Instru_fhr<br>instrumental delivery for abnormal FHR	Categorical<br>0 = no, 1 = yes
	cs-ind<br>(indication for caesarean section)	Categorical<br>0 = not applicable: other MOD<br>1 = failure to progress (FTP)<br>2 = fetal distress<br>3 = failed induction<br>4 = failed instrumental<br>5 = fetal distress + FTP<br>6=maternal complication-specify)<br>7= other<br>8=cord prolapse<br>9=abruption<br>10= fetal distress + FTP
	cs_ftp<br>caesarean section for failure to progress	Categorical<br>0 = no, 1 = yes
	cs_fhr<br>caesarean section for abnormal FHR	Categorical<br>0 = no, 1 = yes
	cs_mat_request<br>caesarean section for maternal request	Categorical<br>0 = no, 1 = yes
	cs_chorio<br>caesarean section for chorioamnionitis as an indication	Categorical<br>0 = no, 1 = yes
	mech-dura<br>(duration of mechanical method in-situ in hours)	Continuous
	More_methods<br>(use of more than one method)<br>More_methods	Categorical<br>0 = no, 1 = yes
	more_methods<br>(more methods for cervical ripening, after the originally allocated method)	Categorical<br>0= no other methods<br>1 = balloon catheter<br>2 = Oxytocin<br>2 = Dilapan-S<br>(please combine if necessary like in 9)

	Idi_hr<br>(induction to delivery interval in hours)	Continuous
	Idi_min (induction to delivery interval in hours)	Continuous
	adm_deli_inte_hr<br>(admission to delivery interval in hours)	Continuous
	Total maternal stay in hours<br>Total_mat_stay	Continuous
	Total neonatal stay in hours<br>Total_neo_stay	
	bs_max (maximum Bishop score recorded/post-intervention)	Continuous
**Secondary labour progression outcomes**	Ut_tachy<br>(uterine tachysystole)	Categorical<br>0 = no, 1 = yes
	Ut_hypersti<br>(uterine hyperstimulation)	Categorical<br>0 = no, 1 = yes
	ut_ hypertonus<br>hypertonus	Categorical<br>0 = no, 1 = yes
	oxy_aug<br>(use of oxytocin during labour)	Categorical<br>0 = no, 1 = yes
	mec_amnio<br>(meconium stained amniotic fluid)	Categorical<br>0 = no, 1 = yes
	analge<br>(analgesia during labour)	Categorical<br>0 = no, 1 = yes
	analge_cat<br>(analgesia during labour)	Categorial<br>0 = None<br>1= Epidural use<br>2 = Parental opioids<br>3 = Entonox<br>4 = Other, specify
	epidural<br>Epidural use	Categorical<br>0 = no, 1 = yes
**Secondary maternal morbidity outcomes**		
	m_anbx (maternal antibiotics given)	Categorical<br>0 = no, 1 = yes
	m_fever (maternal temperature ≥38C)	Categorical<br>0 = no, 1 = yes

	m_infec<br>(suspected/proven maternal infection)	Categorical<br>0 = no, 1 = yes
	m_infec_total = composite of fever, infection all to be used for maternal composite	Categorical<br>0 = no, 1 = yes
	endomet<br>(endometritis suspected or proven)	Categorical<br>0 = no, 1 = yes
	ebl<br>(estimated blood loss in mL)	Continuous
	pph-1000<br>(severe post-partum haemorrhage 1000mL or more)	Categorical<br>0 = no, 1 = yes
	pph500<br>(post-partum haemorrhage 500ml or more)	Categorical<br>0 = no, 1 = yes
	uti<br>(urinary tract infection)	Categorical<br>0 = no, 1 = yes
	bowel<br>(bowel obstruction)	Categorical<br>0 = no, 1 = yes
	vte<br>(thromboembolic event)	Categorical<br>0 = no, 1 = yes
	hyperten<br>(hypertensive disorder in pregnancy)	Categorical<br>0 = no, 1 = yes
	pet<br>(pre-eclampsia/HELLP)	Categorical<br>0 = no, 1 = yes
	icu<br>(maternal ICU admission)	Categorical<br>0 = no, 1 = yes
	ut_rupture<br>(uterine rupture)	Categorical<br>0 = no, 1 = yes
	m_death<br>(maternal death)	Categorical<br>0 = no, 1 = yes
	oth-cx<br>(other postpartum condition requiring hospital admission)	Categorical<br>0 = no, 1 = yes
**Secondary neonatal morbidity outcomes**	stillbirth	Categorical<br>0 = no, 1 = yes
	nnd	Categorical<br>0 = no, 1 = yes
	Apgar<br>Apgar score recorded	Continuous

	Apgar_cat<br>(APGAR score recorded as <7 at 5 minutes)	Categorical<br>0 = no (Good APGAR 7-10 at 5 min)<br>1 = yes (Bad APGAR 0-6 at 5 min)
	fever_neonatal (neonatal temperature ≥38C)	Categorical<br>0 = no, 1 = yes
	Ph_cat<br>(arterial umbilical cord pH <7.10)	Categorical<br>0 = no (pH 7.10 or greater)<br>1 = yes (pH <7.09 or less)
	ph	Continuous
	lactate	Categorical<br>0 = no (<4.79)<br>1 = yes (4.8 or greater)
	nicu<br>(admission to neonatal intensive care unit for any duration)	Categorical<br>0 = no, 1 = yes
	seizures<br>(neonatal seizures)	Categorical<br>0 = no, 1 = yes
	mech_venti<br>(mechanical ventilation)	Categorical<br>0 = no, 1 = yes
	neo_infec<br>(suspected/proven neonatal infection)	Categorical<br>0 = no, 1 = yes
	neo_anbx<br>(neonatal antibiotics given)	Categorical<br>0 = no, 1 = yes
	meconium_syndrome<br>(meconium aspiration syndrome)	Categorical<br>0 = no, 1 = yes
	hie<br>Hypoxic ischaemic encephalopathy	Categorical<br>0 = no, 1 = yes
	respi_dis<br>Severe neonatal respiratory compromise	Categorical<br>0 = no, 1 = yes
	cord<br>Cord Prolapse	Categorical<br>0 = no, 1 = yes
	ecc<br>External cardiac compression	Categorical<br>0 = no, 1 = yes
