# Supplementary material for: Outpatient or Inpatient Setting for Cervical Ripening Before Induction of Labour: An Individual Participant Data Meta‐Analysis
Source: BJOG. 2025 Jun 11;132(13):1966–82. doi: 10.1111/1471-0528.18253 (PMC12592782; doi:10.1111/1471-0528.18253)
Supplement: Supplementary file 3 — Appendix S3. Pre‐defined study protocol. [file BJO-132-1966-s001.pdf]

Research protocol

Title

Outpatient versus inpatient methods for induction of labour: individual participant data meta-analysis.

Members

Monash University; Department of Obstetrics & Gynaecology, Monash Health, VIC, Australia.

Dr Malitha Patabendige

Dr Fei Chan

Dr Daniel Rolnik

Dr Wentao Li

Prof Ben Mol

Synopsis

This individual participant data meta-analysis is intended to evaluate the effectiveness and safety of the methods used for the induction of labour in outpatient versus inpatient settings. This study could enable clinicians to select the most appropriate setting to start the induction of labour.

Introduction

Rationale

Induction of labour (IOL) is one of the most commonly performed obstetric procedures worldwide. It is performed when the medical intervention for initiating labour is deemed necessary compared to the expectant management for the well-being of both the mother and the newborn. Currently, IOL is carried out after admission to the hospital in most settings. In high-income countries, outpatient cervical ripening and IOL are carried out for low-risk women in most metropolitan hospitals, and the balloon catheter is the preferred choice. Potential methods should be effective and safe. Theoretically, outpatient cervical ripening and IOL might yield several potential advantages, such as a reduction in the length of hospital stay, higher maternal satisfaction, and a reduction in cost.

The individual participant data meta-analysis (IPDMA) is carried out using original raw data from the individual researchers via direct contact (1). While the traditional systematic reviews are performed using the data extracted from the published studies along with methodological limitations of aggregate data, IPDMA generates a single central database to be re-analysed and combined, if appropriate, as a meta-analysis (2). For this reason, IPDMA is known as the 'gold standard of systematic reviews, yielding a more substantial amount of quality data to guide the clinical practice (1, 2).

The recent aggregate data meta-analysis comparing outpatient and inpatient cervical ripening with balloon catheters by Pierce-Williams et al. has provided some positive insights (3). However, before concluding to change the clinical practice based on an aggregate data meta-analysis, performing an IPDMA is worthwhile as it carries better trustworthiness and a more flexible analysis using raw data. The existing meta-analysis by Pierce-Williams et al. has acknowledged the smaller sample size for the assessment of perinatal safety outcomes and maternal outcomes has not been properly studied in any of the studies (3). This issue might be addressed in an IPDMA using raw data as composite safety outcomes could be constructed. There are a few trials comparing prostaglandins for both outpatient and inpatient arms. Cochrane review on the 'Home

versus inpatient induction of labour for improving birth outcomes' has summarised randomised controlled trials studying prostaglandins and balloon catheters up to 2020 (4). Similarly, a systematic review and meta-analysis by Dong et al. in 2020 mentioned similar conclusions to this Cochrane review (5). Overall, there is limited data on the effectiveness and safety of IOL in the outpatient setting, with a highly variable consumer uptake worldwide. Most available randomised controlled trials are small, and minimal data is available from low-resource settings. All of these are aggregate data meta-analyses; hence, the value of individual participant data meta-analysis would be able to understand the exact situation of the strength of the evidence. Therefore, we have planned an IPDMA to compare the effectiveness and safety of the outpatient versus inpatient methods in IOL with different methods.

Objectives

1. To compare the overall effectiveness and safety of induction of labour in the outpatient setting versus the inpatient setting, irrespective of the methods used.
2. To compare the effectiveness and safety of induction of labour in the outpatient setting versus the inpatient setting when balloon catheters are used for both settings.
3. To compare the effectiveness and safety of induction of labour in the outpatient setting versus inpatient setting when vaginal prostaglandins are used in both settings.
4. To compare the effectiveness and safety of induction of labour in the outpatient setting versus the inpatient setting when any other method was used in one or both settings, i.e., osmotic dilators, dinoprostone, and oral/vaginal misoprostol.
5. To compare the effectiveness and safety of induction of labour using balloon catheters in the outpatient setting versus vaginal prostaglandins E2 in the inpatient setting.
6. To compare the overall effectiveness and safety of induction of labour in the outpatient setting versus the inpatient setting when mechanical methods are used in both settings.

The 5th objective, the effectiveness and safety of IOL using balloon catheters in the outpatient setting versus vaginal prostaglandins E2 in the inpatient setting, are being assessed as a separate IPDMA. However, data will be used to compare this IPDMA (first objective) overall.

Methods

1. **Search strategy and eligibility criteria**

Randomised controlled trials with outpatient and inpatient cervical ripening and/or IOL for women with unfavourable cervices will be included. All pharmacological and mechanical methods currently used in outpatient and inpatient settings will be included, irrespective of the gestational age. Membrane sweeping, nipple stimulation, or other non-pharmacologic and non-mechanical methods not in line with contemporary obstetrical practice will be excluded. In other words, only the currently recommended labour induction methods per major clinical guidelines (NICE, ACOG, SOGC, and RANZCOG) will be included. In addition, trials with only an active intervention in both arms will be included, and therefore, trials with the placebo effect and expectant management will be excluded. Women will be included irrespective of gestational age, membrane status, pre-term labour and previous caesarean or scar pregnancy. Cluster randomised trials and quasi-experimental trials will be excluded.

Potentially suitable clinical trials will be identified from inception to September 2023. Databases including Ovid MEDLINE, Ovid Embase, Ovid Emcare, CINAHL Plus, Scopus, Cochrane Pregnancy and Childbirth Group's Trials Register, the WHO International Clinical Trials Registry Platform (ICTRP) and clinicaltrials.gov (for unpublished, planned, and ongoing trial reports). Reference lists of all included studies, and previously conducted systematic reviews will be searched for articles that might have been missed through the formal search (citation-tracking).
Cochrane review on the 'Home versus inpatient induction of labour for improving birth outcomes' has summarised randomised controlled trials studying prostaglandins and balloon catheters up to 2020 and another systematic review and meta-analysis by Pierce et al. compared trials where balloon catheters used for both settings (3, 4). These two meta-analyses can also be a guide to tracing the recent studies. There will be no language barriers, and all the published and unpublished data will be eligible.

Two investigators will independently review the identified papers for eligibility, with disagreements to be solved by a third reviewer. The principal authors of eligible trials will subsequently be invited to contribute their raw data for analysis. An IPDMA will be performed on available data to assess the impact on primary and secondary outcome measures, as outlined below.

2. Data extraction and the invitation to authors for IPDMA

The principal investigators of all eligible trials identified will be contacted via email to participate in this study. If there is no response for two weeks, another gentle reminder will be emailed. They will be asked to share the individual patient data collected from their trials. If they fail to respond, the co-authors recent co-authors of other publications and institutions can be contacted. The principal investigators will be asked to complete an Excel datasheet or send their data with a document clearly stating the definitions used for variables. The collected anonymous data should contain baseline patient characteristics, including age, gestational age, parity, ethnicity, body mass index (BMI), pre-pregnancy and randomisation, smoking status, and modified Bishop score before intervention. Additionally, indications for IOL and the outcomes of interest will be listed.

3. Outcomes and subgroups

The primary outcomes of this study cover the effectiveness and safety in the outpatient setting. The primary outcome for effectiveness is the vaginal birth rate. Perinatal and maternal safety will be assessed as follows.

- Adverse perinatal outcomes: a composite measure of stillbirth, neonatal death, neonatal Apgar score <7 at 5 minutes, acidosis (pH<7.1), neonatal seizures, hypoxic ischaemic encephalopathy of any stage (HIE), neonatal intensive care unit (NICU) admission, meconium aspiration syndrome, neonatal infection either clinically suspected (as defined by neonatal antibiotic administration) or proven neonatal infection (culture proven), cord prolapse, endotracheal intubation and external cardiac compressions.

- Adverse maternal outcomes: a composite measure of admission to ICU for any period, maternal infection (defined as a temperature ≥ 38°C at any time during labour or delivery or antibiotic use or clinically diagnosed infection, such as endometritis), postpartum haemorrhage ≥1000 mL, maternal death, uterine rupture.

Secondary outcomes could further explore the effectiveness and safety of IOL in each setting and maternal and neonatal complications.

- Time from commencement of labour induction to delivery

- Overall caesarean delivery rate and indication for caesarean delivery - fetal compromise or failure to progress). If both fetal compromise and failure to progress apply as indications, fetal compromise will prevail.
- Instrumental vaginal birth and indication for instrumental vaginal birth- fetal compromise or failure to progress). If both fetal compromise and failure to progress apply as indications, fetal compromise will prevail.
- Change in modified Bishop Score
- Uterine hyperstimulation, tachysystole and hypertonus
- Requirement for oxytocin infusion
- Unsuccessful induction (defined as a change in induction method from the originally allocated method)
- Unexpected home birth
- Admission to delivery interval and total hospital stay for mother and neonate
- Use of epidural analgesia during labour
- We will conduct subgroup analysis for each of the perinatal outcomes separately: stillbirth, neonatal death, neonatal Apgar score <7 at 5 minutes, acidosis (pH<7.1), neonatal seizures, hypoxic ischaemic encephalopathy of any stage (HIE), neonatal intensive care unit (NICU) admission for any duration, meconium aspiration syndrome, neonatal infection either clinically suspected (as defined by neonatal antibiotic administration) or proven neonatal infection (culture proven), cord prolapse, endotracheal intubation and/or external cardiac compressions.
- We will conduct subgroup analysis for each of the maternal parameters separately: admission to ICU for any period, maternal infection (defined as a temperature ≥ 38°C at any time during labour or delivery or antibiotic use or clinically diagnosed infection, such as endometritis), postpartum haemorrhage ≥1000 mL, maternal death, uterine rupture.
- Maternal satisfaction data

4. Data cleaning, synthesis and statistical analysis

We will construct the PRISMA-IPD flow diagram(6) detailing the study selection process and collection of IPD and aggregate data. This diagram will report the number of studies identified, screened for eligibility, and included or excluded, together with the number of participants in these studies and the reasons for

exclusion at each stage. The flow diagram will also report the number of studies that provided IPD and those that did not, along with the reasons why IPD could not be obtained in the latter case and the number of participants for which IPD could or could not be obtained.

The analysis will be performed for the–objectives separately. Data from the participating trials for the predefined variables of the primary and secondary outcomes, including baseline characteristics, will be identified. The baseline characteristics of each trial will be presented in a table with a separate column for each trial. Variables not collected in the individual trial will be indicated with a consistent code, allowing for identification and omission from relevant analyses. Each trial's data will be checked for discrepancies, range, internal consistency, missing or extreme values and errors. When there is a doubt regarding these, principal investigators will be contacted for clarification. Each individual trial will be re-analysed separately before being included in the central IPD dataset. The analysis will be on an intention-to-treat basis. Summary tables will be created and will be shared with the principal investigator of each study. Discrepancies between these summaries and the published data will be discussed with the principal investigators. Corrections will be made where relevant. If any questions are raised, the investigators will be contacted for clarification.

Outcomes will be pooled using the two-stage random-effects model as the first choice since we expect clinical and methodological heterogeneity between studies. The one-stage analysis will be performed for the primary outcomes as a sensitivity analysis. In the case of zero events in any group in any trial for an outcome, the one-stage analysis will be the first choice. For dichotomous outcomes, odds ratio (OR) and 95% CI will be used. For continuous variables, mean differences (MD) and 95% CI will be computed. For time-to-event outcomes (i.e., time to vaginal birth and time to spontaneous vaginal birth), subdistribution hazard ratio (SHR) and 95% CI using a subdistribution hazard competing-risks model, considering caesarean delivery as a competing risk will be used. Statistical heterogeneity will be assessed using the Tau^2, I^2 and Chi^2 statistics. using the I^2 statistic, treating I^2 values > 50 as having a high degree of statistical heterogeneity (7). Heterogeneity will be regarded as substantial if I^2 is greater than 50%. Either Tau^2 is greater than zero, or there is a low P value (less than 0.10) in the Chi^2 test for heterogeneity.

Subgroup analysis will also be conducted for induction to delivery interval, admission to delivery interval, and total hospital stay for the mother and neonate. Subgroup analyses for the primary outcomes will be assessed using interaction terms between treatment and baseline covariates. This will also be performed for

each of the objectives. This is recommended to avoid ecologic bias (aggregation bias) within-trial interaction concerned. Subgroup analyses will be explored for parity (nulliparous versus parous), maternal age, body mass index, and gestational age at induction (as a continuous and grand-mean-centred variable). In addition, different induction methods will be assessed in separate comparisons (if there is more than one similar study), and balloon catheters and osmotic dilators will also be analysed as mechanical methods separately. Maternal satisfaction data will be assessed if more than one study has data and the ability to harmonise qualitative or quantitative data for pooling (if sufficient data is available).

Labour and delivery outcomes and composite maternal and neonatal outcomes will be presented in forest plots separately. For adverse outcomes, an OR >1.0 indicates that the outpatient group may confer a greater risk, whereas, for positive outcomes (for example, vaginal birth), an SHR or OR >1.0 indicates that the outpatient group may be more favourable.

For a vaginal birth, the finding of this study will be compared with the findings of the meta-analysis using aggregate data of the same trials to assess the IPD availability bias. Statistical analyses will be performed using the R Foundation for Statistical Computing, Vienna, Austria (8): "meta" package will be used for the two-stage meta-analysis and aggregate data meta-analysis, “lme4”, package will be used for one stage meta-analysis and the “survival” package used for competing risk analysis. Statistical package for social sciences (IBM SPSS statistics for Windows version 28.0. Armonk, NY: IBM Corp) will be used for summary statistics. A P-value less than 0.05 will be considered as statistically significant.

5. Risk of bias and GRADE assessment

Risk of bias will be assessed according to the revised Cochrane risk-of-bias tool for randomised trials and a summary table will be prepared, including assessments for all the included studies (9). For each trial, the following domains will be assessed: randomization process; deviations from intended interventions, missing outcome data, measurement of the outcome, and selection of the reported result. These will be graded as either low risk, having some concerns or high risk and will be demonstrated in figures. Discrepancies in the assessments between the two reviewers will be resolved by discussion to reach a consensus. The Grading of Recommendations, Assessment, Development and Evaluation (GRADE) approach to assess the overall certainty of evidence for the primary outcomes (10).

Role of the funding source

This study is supported by two NHMRC Investigator Grants (GNT1176437 for BWM and GNT2016729 for WL). These funding sources had no role in the design, execution, analyses, or data interpretation for this research. MP is supported by a Research Training Stipend, provided by the Australian Government.

Governance

For an IPDMA, the data will be requested in a de-identified state from the original authors who will need/ who have local ethics approval to share the data. This project was registered at PROSPERO (CRD42022313183), and the reporting of this study will follow the Preferred Reporting Items for Systematic Review and Meta-Analyses of Individual Participant Data (PRISMA-IPD) statement. The project was approved by the Human Research Ethics Committee (ERM Reference No. 84527, Monash Health Local Reference: RES-22-0000-119Q), Monash Health, VIC, Australia.

Timeline

Dates	Aims
Feb 6 – May 15, 2022	Learning search strategies<br>Identification and collection of eligible trials<br>Formation of protocol<br>Contact with and invitation to trial authors*
May 16 – June 30, 2022	Literature review

	Collaboration with trial authors on protocol
July 1, 2022 – Jan 1, 2024	Data cleaning and statistical analysis of provided data<br>Collaboration with trial authors<br>Generating data for meta-analysis
Jan 1, 2024– onwards**	Further data cleaning and statistical analysis<br>Collaboration with trial authors<br>Formation of meta-analysis

*subject to finding contact details of the author.

**subject to late expression of interest by author, and data retraction by author.

Table 1: Approximate timeline in conducting and completing this research project.

References

1. Tierney JF, Vale C, Riley R, Smith CT, Stewart L, Clarke M, et al. Individual Participant Data (IPD) Meta-analyses of Randomised Controlled Trials: Guidance on Their Use. PLoS Med. 2015;12(7):e1001855.
2. Ventresca M, Schünemann HJ, Macbeth F, Clarke M, Thabane L, Griffiths G, et al. Obtaining and managing data sets for individual participant data meta-analysis: scoping review and practical guide. BMC Med Res Methodol. 2020;20(1):113.
3. Pierce-Williams R, Lesser H, Saccone G, Harper L, Chen V, Sciscione A, et al. Outpatient Cervical Ripening with Balloon Catheters: A Systematic Review and Meta-analysis. Obstet Gynecol. 2022;139(2):255-68.
4. Alfirevic Z, Gyte GM, Nogueira Pileggi V, Plachcinski R, Osoti AO, Finucane EM. Home versus inpatient induction of labour for improving birth outcomes. Cochrane Database Syst Rev. 2020;8:CD007372.
5. Dong S, Khan M, Hashimi F, Chamy C, D'Souza R. Inpatient versus outpatient induction of labour: a systematic review and meta-analysis. BMC Pregnancy Childbirth. 2020;20(1):382.
6. Stewart LAea. 'Preferred Reporting Items for Systematic Review and Meta-Analyses of individual participant data: the PRISMA-IPD Statement.'. JAMA. 2015;313(16), pp. 1657–65.

7. Ioannidis JP, Patsopoulos NA, Evangelou E. Uncertainty in heterogeneity estimates in meta-analyses. BMJ. 2007;335(7626):914-6.
8. R Foundation for Statistical Computing, Vienna, Austria. R Core Team. R: A language and environment for statistical computing. 2022.
9. Sterne JAC, Savović J, Page MJ, Elbers RG, Blencowe NS, Boutron I, et al. RoB 2: a revised tool for assessing risk of bias in randomised trials. BMJ. 2019;366:l4898.
10. Sch ¨unemann H BzJ, Guyatt G OA. Handbook for grading the quality of evidence and the strength of recommendations using the GRADE approach. Updated in October 2013.
