# Supplementary material for: Outpatient or Inpatient Setting for Cervical Ripening Before Induction of Labour: An Individual Participant Data Meta‐Analysis
Source: BJOG. 2025 Jun 11;132(13):1966–82. doi: 10.1111/1471-0528.18253 (PMC12592782; doi:10.1111/1471-0528.18253)
Supplement: Supplementary file 4 — Data S1. [file BJO-132-1966-s004.docx]

**Title: Outpatient or inpatient setting for cervical ripening before induction of labour: an individual participant data meta-analysis.**

**Supplementary Files: Figures and Tables**

**Supplementary Figures**

Figure S1: Risk of bias summary for trials providing IPD and included in the meta-analysis (N=11).


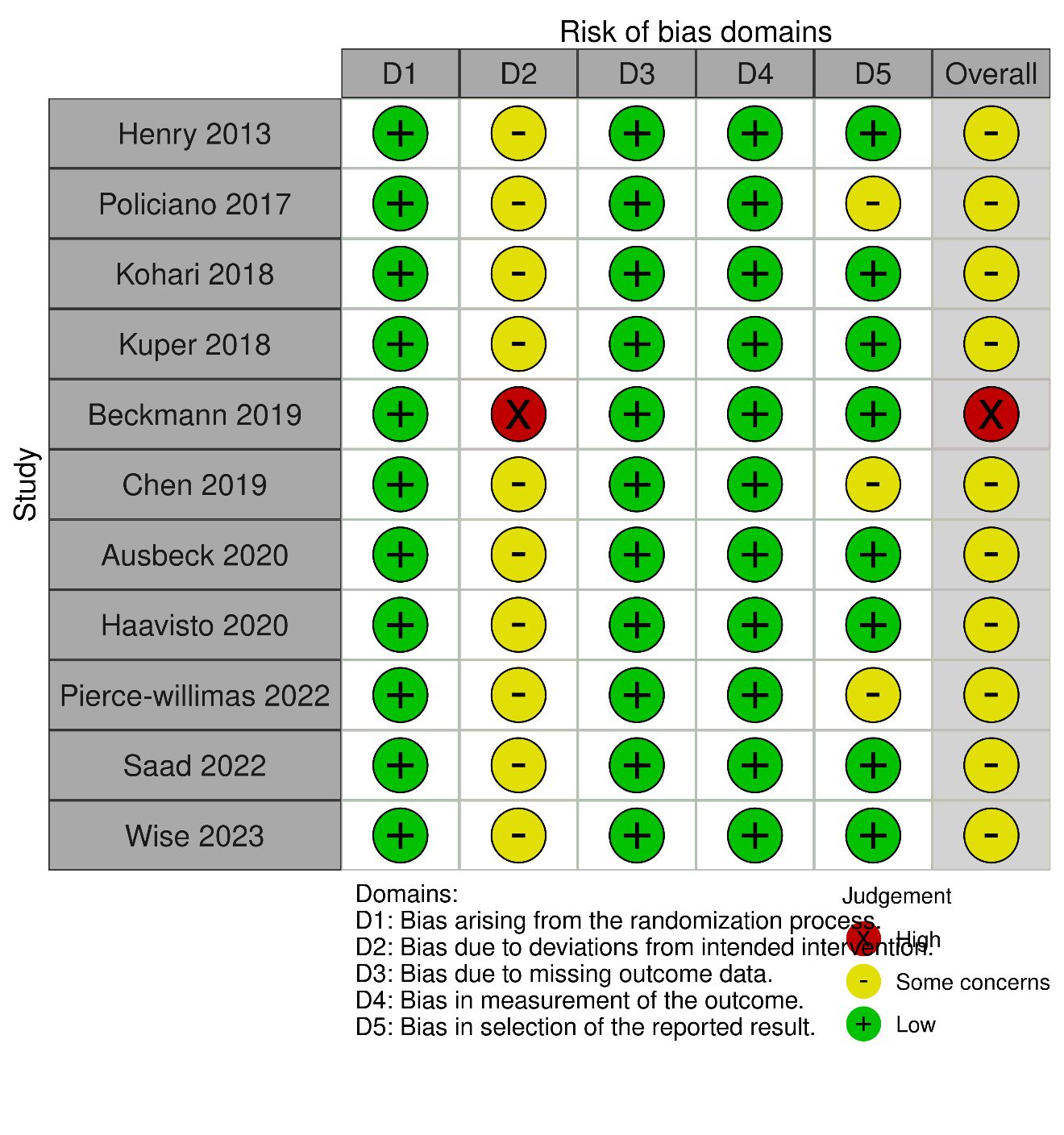


Figure S2: Risk of bias summary for trials that did not provide IPD (N=8).

**
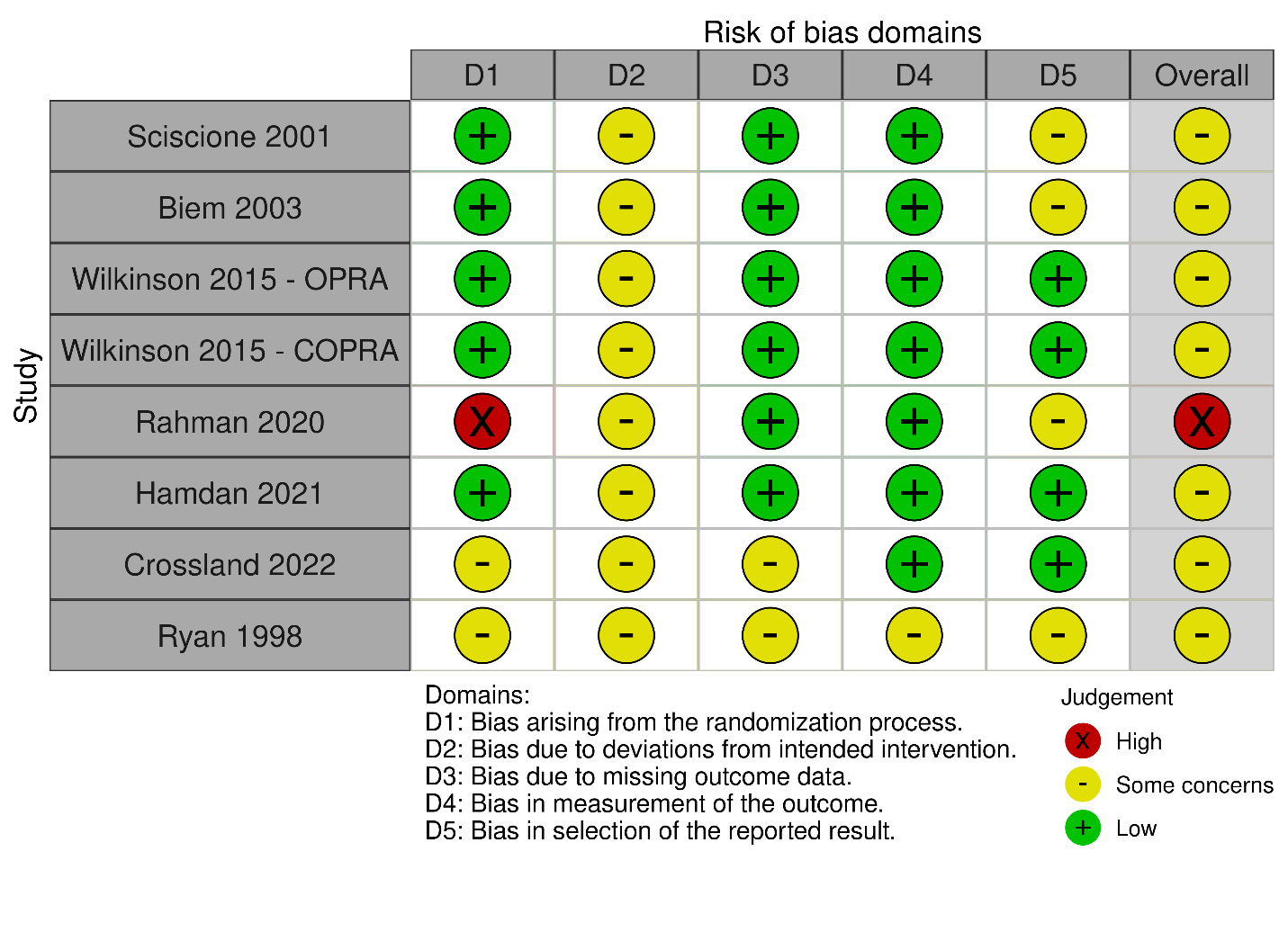
**

**Figures S3 - S10 show the secondary outcomes results for the overall comparison with all the methods for cervical ripening and labour induction.**


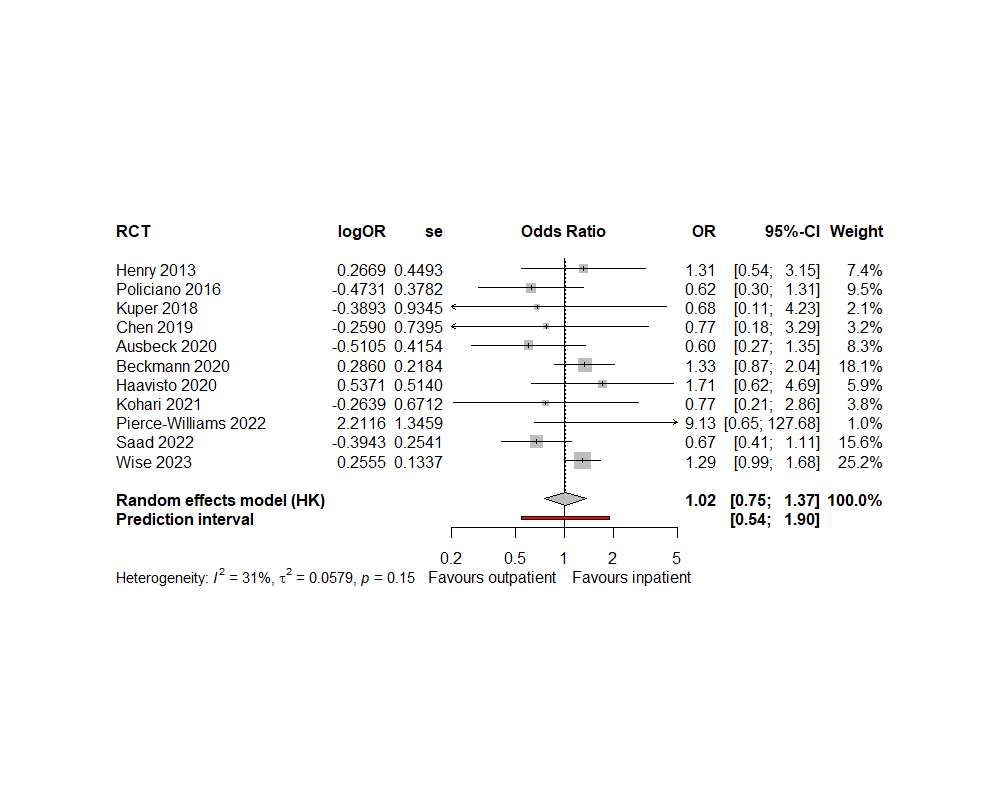
Figure S3: Rate of caesarean deliveries (two-stage meta-analysis).


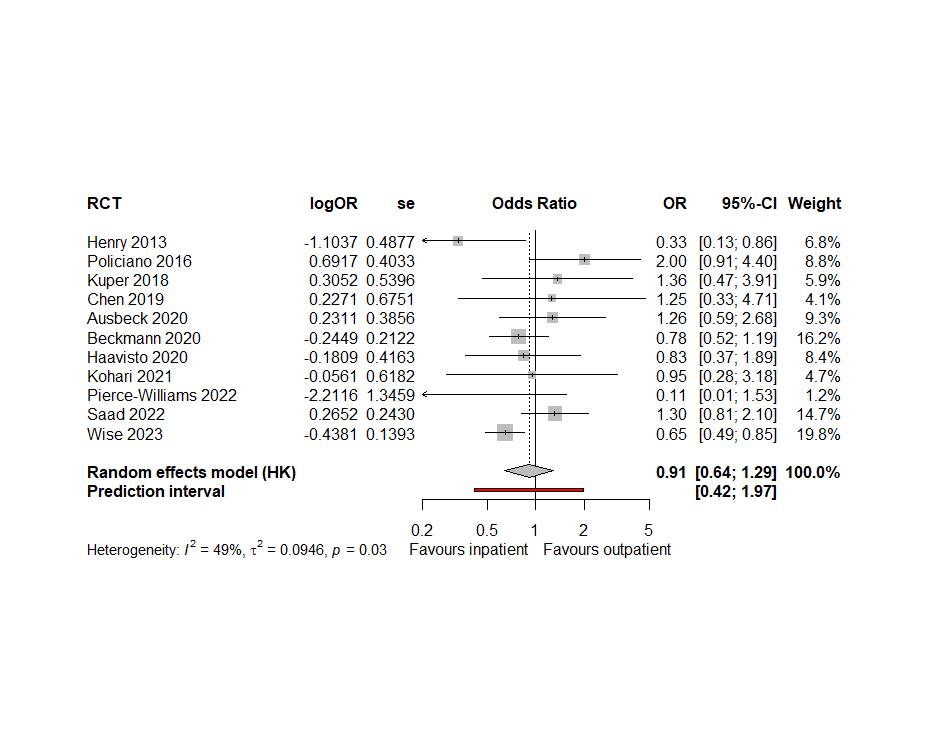
Figure S4: Rate of unassisted vaginal deliveries (two-stage meta-analysis).


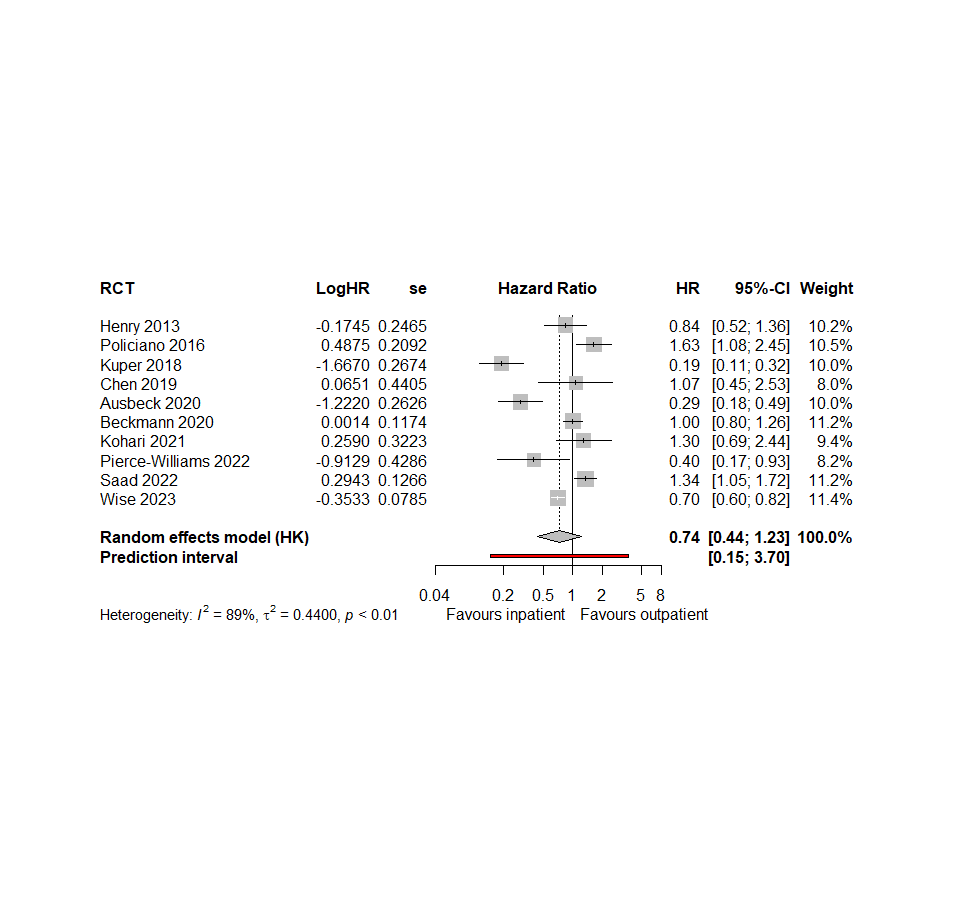
Figure S5: Time to vaginal birth (two-stage meta-analysis).


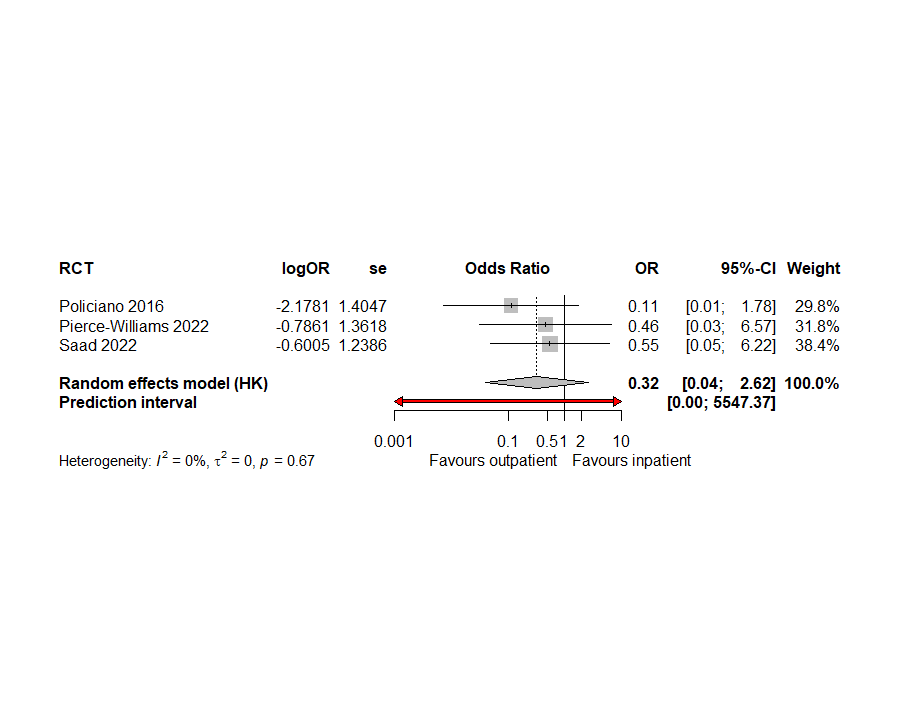
Figure S6: Uterine tachysystole (two-stage meta-analysis).


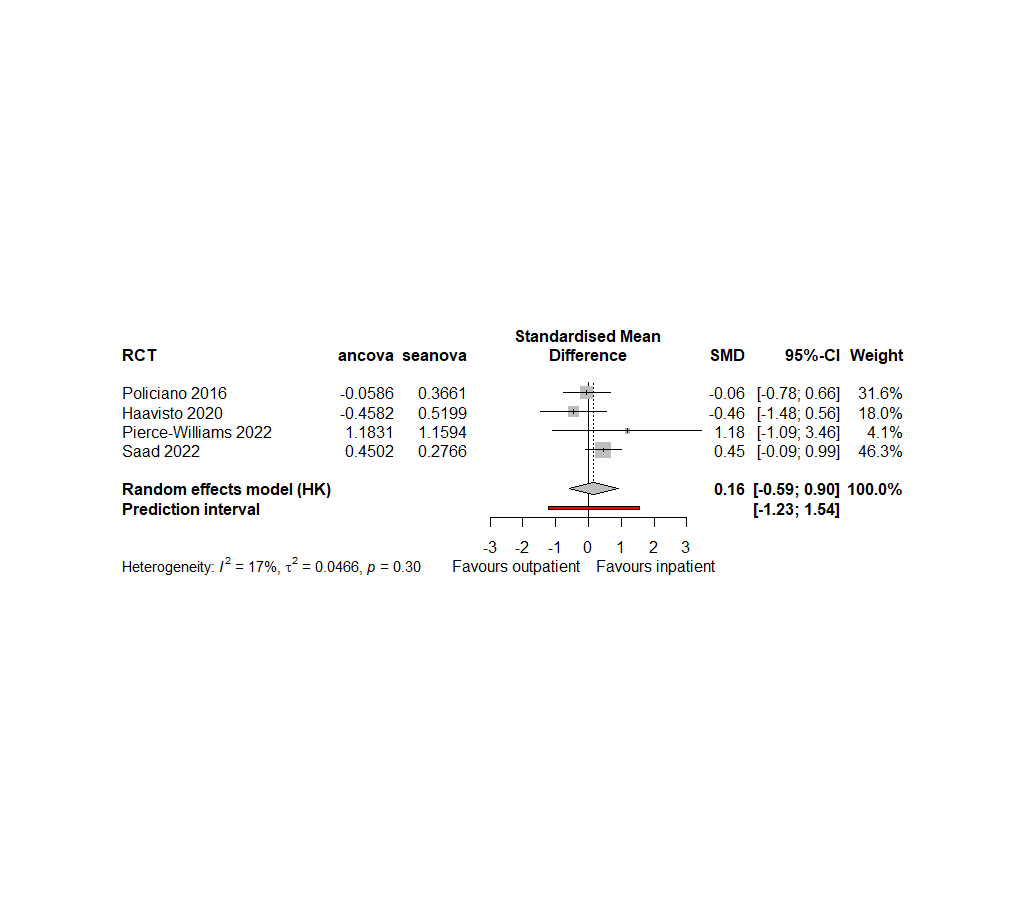
Figure S7: Pain during cervical ripening (two-stage meta-analysis).


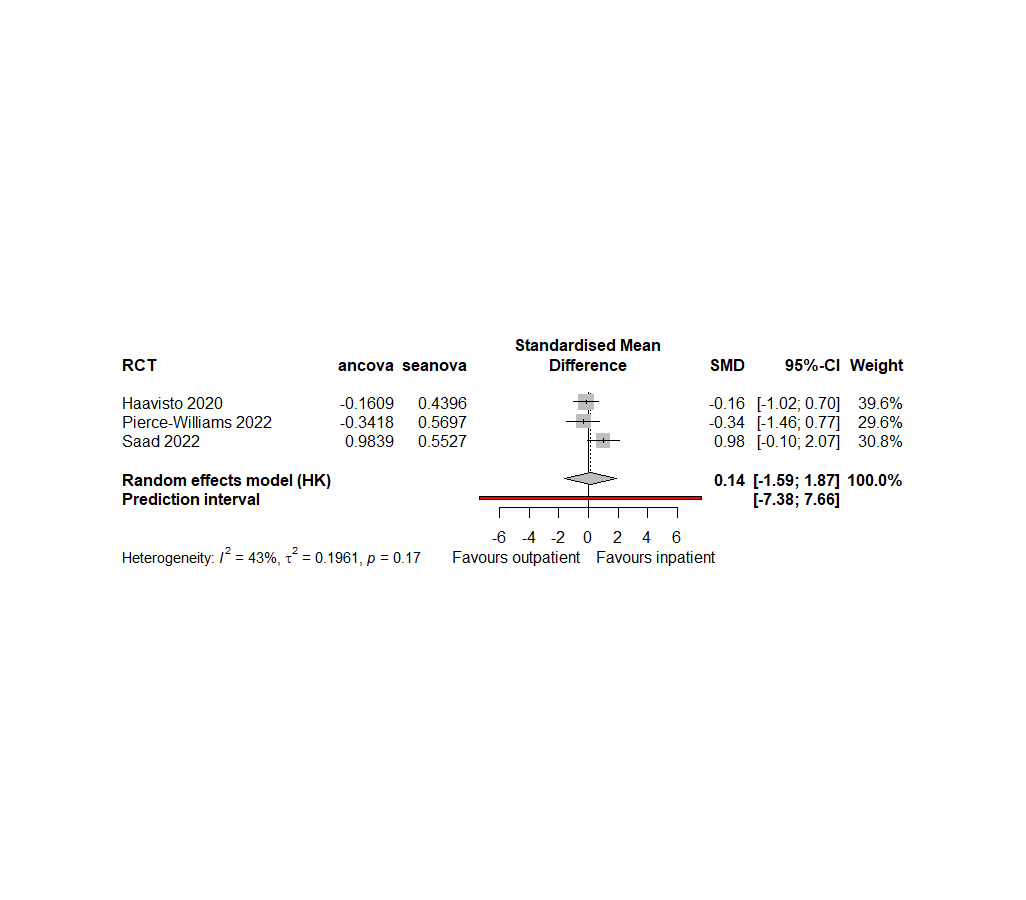
Figure S8: Overall satisfaction score (two-stage meta-analysis).

Figure S9: Use of epidural analgesia (two-stage meta-analysis).


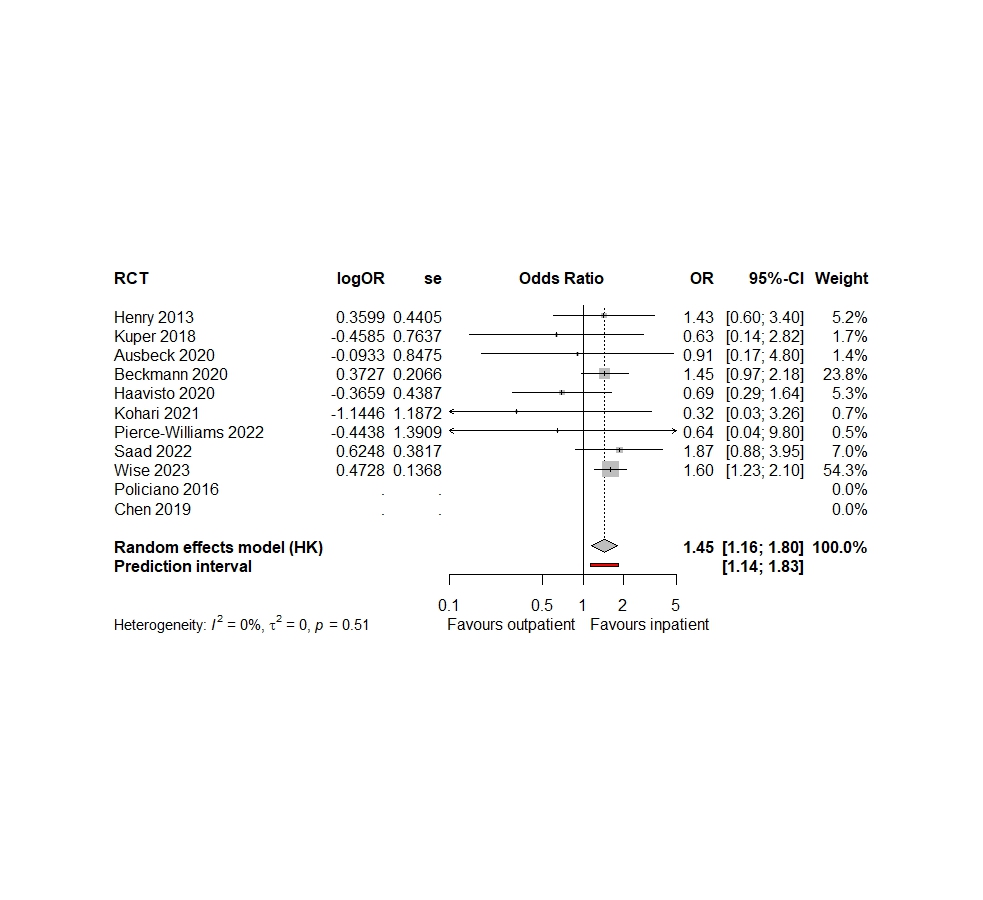


Figure S10: Use of more than one method for cervical ripening (two-stage meta-analysis).


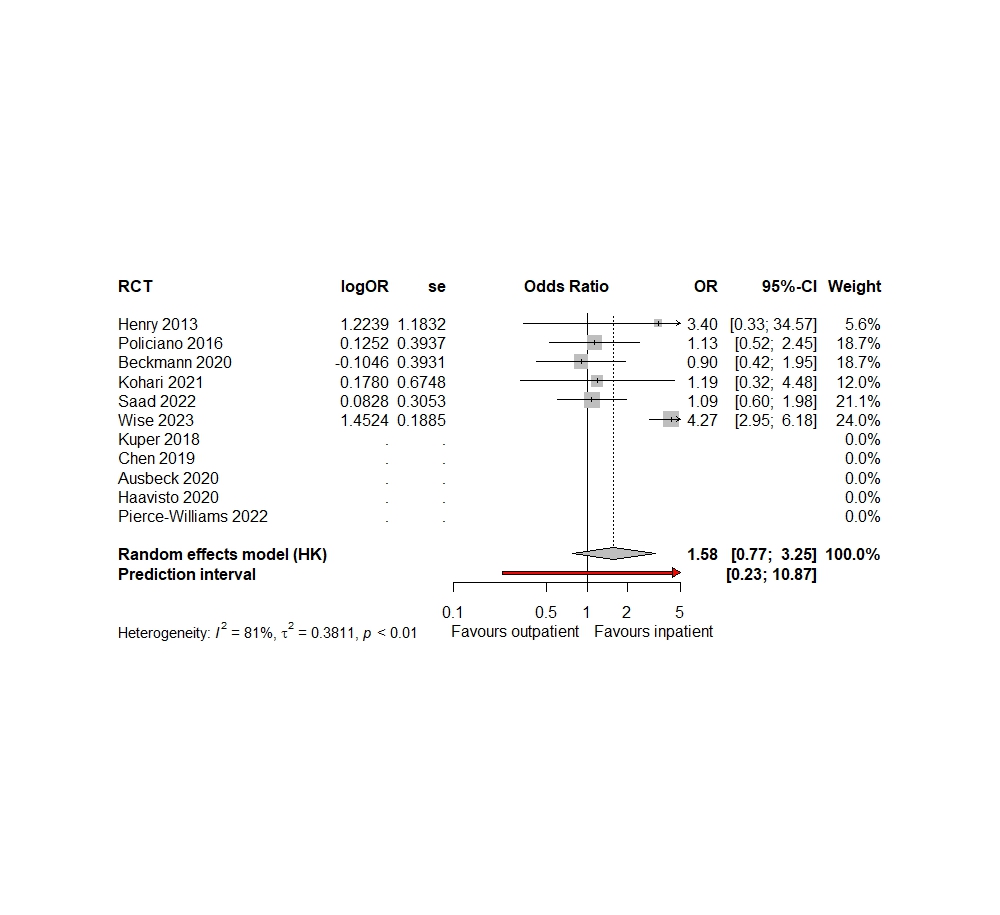


**Figures S11 - S13 show forest plots for the secondary outcomes of balloon catheter use in outpatient and inpatient groups.**


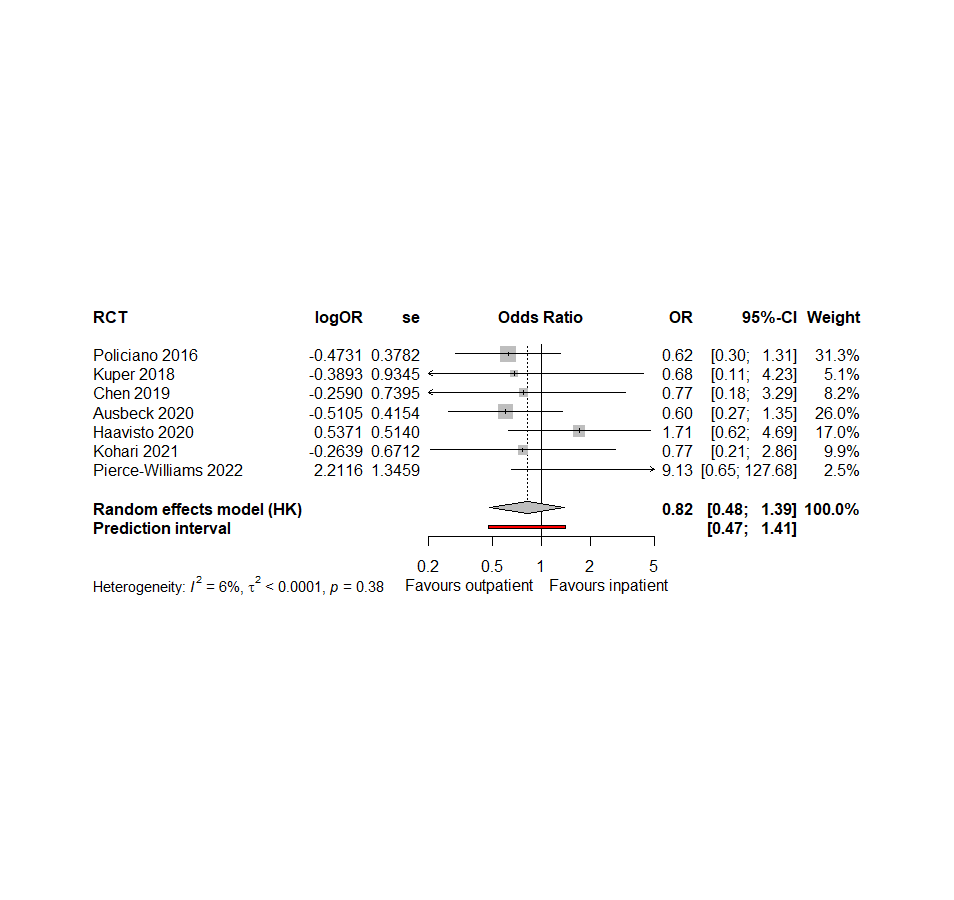
Figure S11: Rate of caesarean deliveries (two-stage meta-analysis).

Figure S12: Rate of unassisted vaginal deliveries (two-stage meta-analysis).


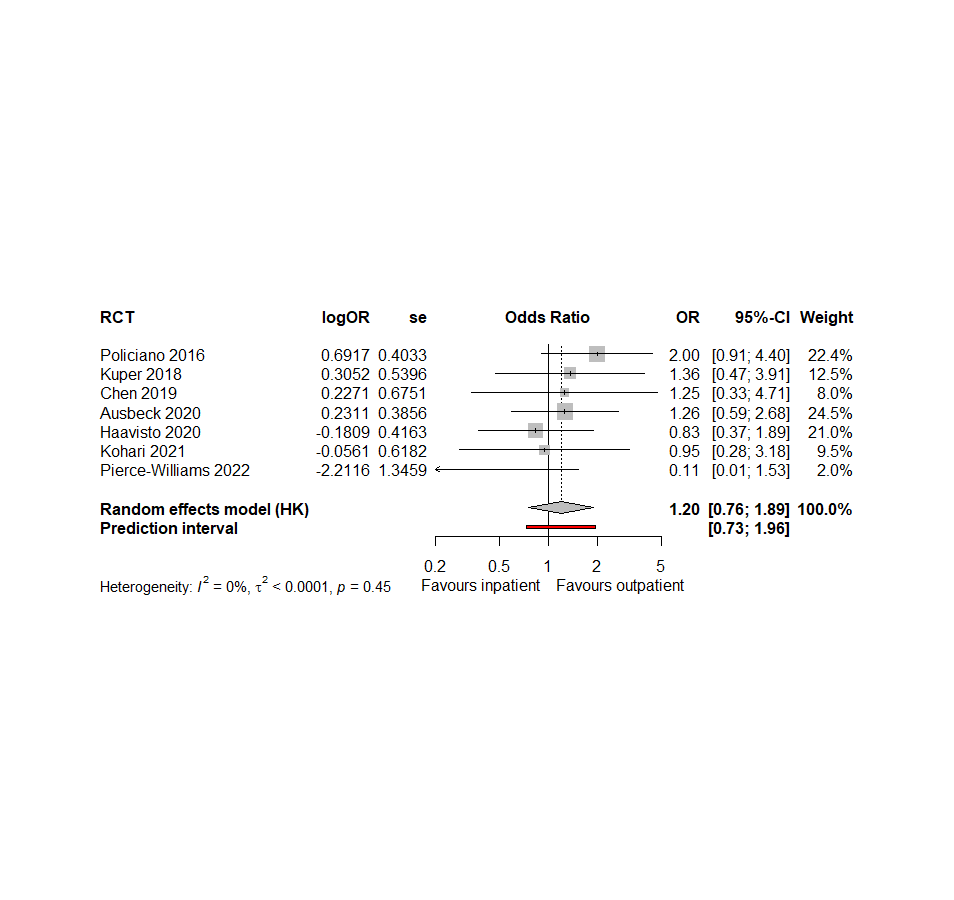


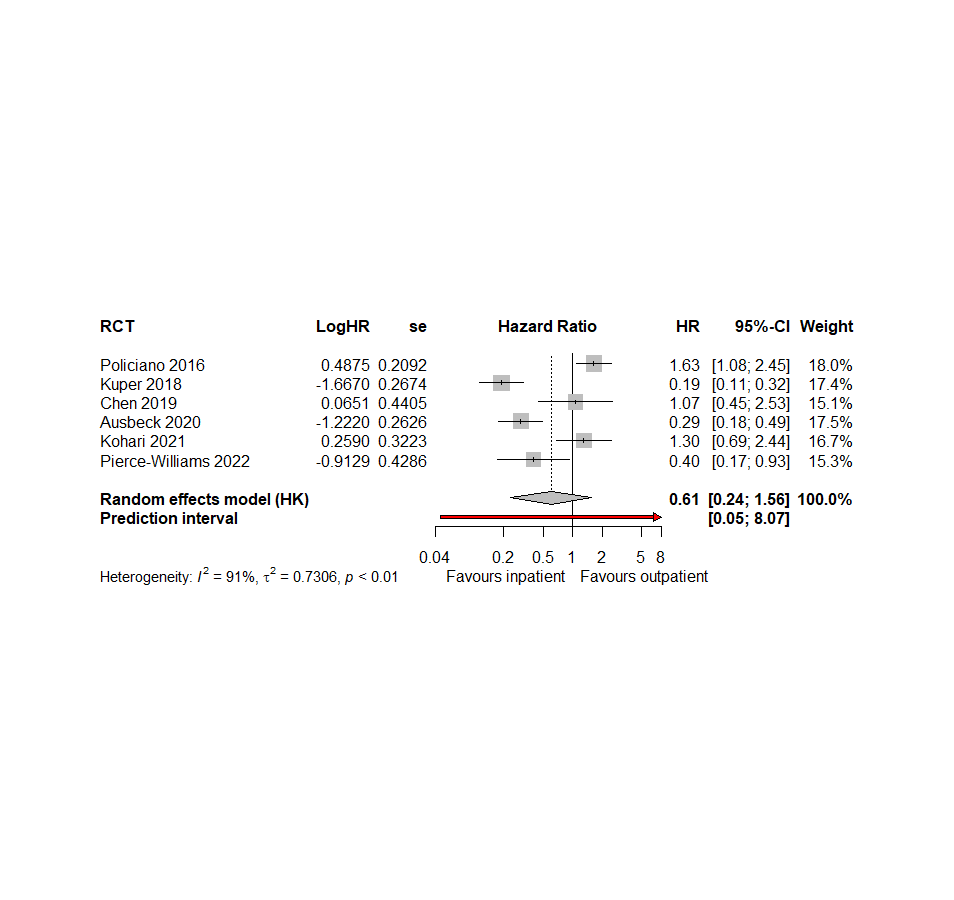
Figure S13: Time to vaginal birth (two-stage meta-analysis).

**Figure S14: Forest plots of IPD meta-analyses of the primary outcomes according to the body mass index category -** **overall comparison of all the methods in both groups.**

Figure S14A: Primary outcomes: Odds of vaginal delivery between two methods (one-stage).

| **BMI category** | **No. of women: Outpatient vs inpatient** | **aOR (95% CI), p-value** |
| --- | --- | --- |
| Underweight/  normal BMI | 324/453 vs 325/466 | 1.09 (0.81;1.46), p=0.56 |
| Overweight | 228/349 vs 244/342 | 0.80 (0.38;1.68), p=0.55 |
| Obese | 310/469 vs 342/489 | 0.91 (0.58;1.43), p=0.68 |

Figure S14B: Primary outcomes: Composite perinatal outcomes between two methods (one-stage).

| **BMI category** | **No. of women: Outpatient vs inpatient** | **aOR (95% CI), p-value** |
| --- | --- | --- |
| Underweight/  normal BMI | 49/453 vs 61/466 | 0.82 (0.47;1.42), p=0.47 |
| Overweight | 36/350 vs 40/343 | 0.86 (0.53;1.40), p=0.54 |
| Obese | 51/469 vs 46/490 | 1.20 (0.79;1.82), p=0.38 |

Figure S14C: Primary outcomes: Composite maternal outcomes between two methods (one-stage).

| **BMI category** | **No. of women: Outpatient vs inpatient** | **aOR (95% CI), p-value** |
| --- | --- | --- |
| Underweight/  normal BMI | 63/453 vs 71/466 | 0.93 (0.65;1.36), p=0.73 |
| Overweight | 46/350 vs 49/343 | 0.85 (0.55;1.33), p=0.48 |
| Obese | 71/469 vs 78/490 | 0.94 (0.66;1.35), p=076 |

**Figure S15: Forest plots of IPD meta-analyses of the primary outcomes according to the body mass index category - balloon catheter use in both outpatient and inpatient groups.**

Figure S15A: Primary outcomes: Odds of vaginal delivery between two methods (one-stage).

| **BMI category** | **No. of women: Outpatient vs inpatient** | **aOR (95% CI), p-value** |
| --- | --- | --- |
| Underweight/  normal BMI | 80/102 vs 76/95 | 0.91 (0.45;1.84), p=0.79 |
| Overweight | 59/79 vs 71/84 | 0.67 (0.19;2.31), p=0.53 |
| Obese | 100/127 vs 84/123 | 1.60 (0.70;3.66), p=0.27 |

Figure S15B: Primary outcomes: Composite perinatal outcomes between two methods (one-stage).

| **BMI category** | **No. of women: Outpatient vs inpatient** | **aOR (95% CI), p-value** |
| --- | --- | --- |
| Underweight/  normal BMI | 6/102 vs 4/95 | 1.80 (0.43;7.53), p=0.42 |
| Overweight | 7/80 vs 4/85 | 1.66 (0.49;5.68), p=0.42 |
| Obese | 7/127 vs 9/124 | 0.83 (0.33;2.04), p=0.68 |

Figure S15C: Primary outcomes: Composite maternal outcomes between two methods (one-stage).

| **BMI category** | **No. of women: Outpatient vs inpatient** | **aOR (95% CI), p-value** |
| --- | --- | --- |
| Underweight/  normal BMI | 9/102 vs 8/95 | 1.11 (0.38;3.26), p=0.84 |
| Overweight | 7/80 vs 5/85 | 1.29 (0.35;4.76), p=0.70 |
| Obese | 17/127 vs 16/124 | 1.18 (0.55;2.58), p=0.67 |

Figure S16: Funnel plot for the primary outcome, vaginal birth. All RCTs (participating and non-participating) (N=19).


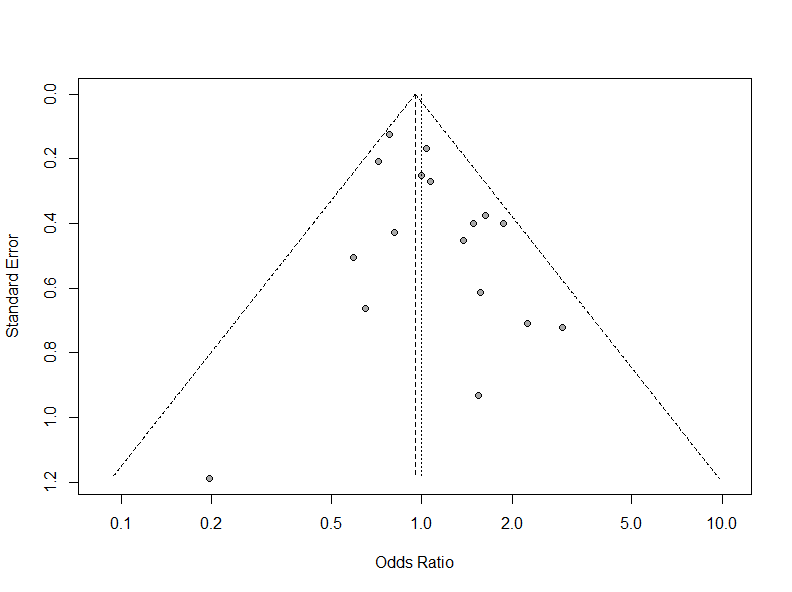


RCT: Randomised controlled trials.

Figure S17: Aggregate data meta-analysis for vaginal birth for all RCTs that did not provide IPD and IPD provided (N=19). Outcomes data were not available from two non-shared RCTs.


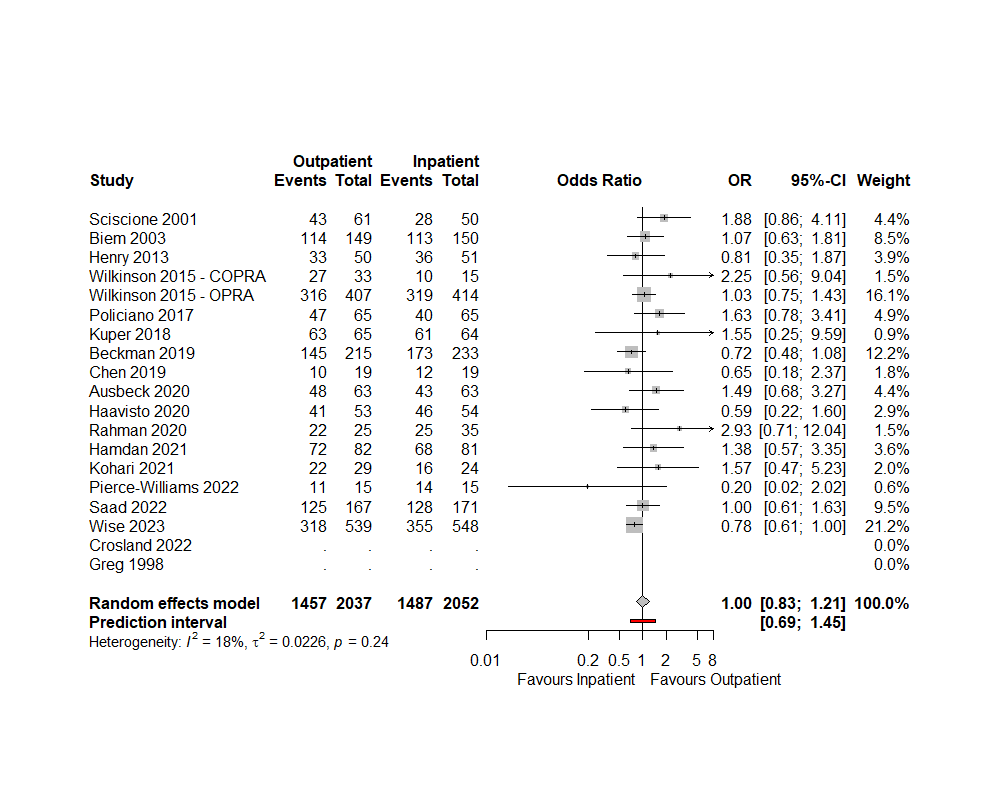


RCT: Randomised controlled trials.

**Supplementary Tables**

Table S1: Definition of outcomes and potential effect modifiers.

| **PRIMARY OUTCOMES**  **Vaginal delivery rate:** rate of vaginal delivery.  **Composite of adverse perinatal outcome:** a composite measure of stillbirth, neonatal death, neonatal Apgar score <7 at 5 minutes, acidosis (pH <7.1), neonatal seizures, hypoxic-ischemic encephalopathy of any stage (HIE), neonatal intensive care unit (NICU) admission, meconium aspiration syndrome^¶^, neonatal infection either clinically suspected (as defined by neonatal antibiotic administration) or proven neonatal infection (culture proven), cord prolapse, endotracheal intubation and/or external cardiac compressions.  **Composite of adverse maternal outcome:** maternal infection (temperature ≥ 38°C at any time during labour or delivery, antibiotics use or clinically diagnosed infection, such as endometritis), maternal admission to intensive care unit, severe postpartum haemorrhage (>1000mL estimated blood loss), uterine rupture or maternal death.  **SECONDARY OUTCOMES**  **Delivery outcome:**   - Cumulative rate of vaginal birth (time-to-event analysis) - Time from commencement of labour induction to delivery - Admission to birth interval - Mode of delivery (caesarean delivery, unassisted vaginal birth, instrumental vaginal birth) - Caesarean delivery, including analysis for fetal compromise or failure to progress (if both fetal compromise and failure to progress apply as indications, fetal compromise prevails). - Indication for instrumental vaginal birth - Total hospital stays for the mother and neonate*   **Labour progression outcomes:**   - Uterine tachysystole - Uterine hyperstimulation - Oxytocin augmentation - Meconium-stained amniotic fluid - Use of epidural analgesia - Use of more methods for cervical ripening (second methods)   **Neonatal safety outcomes:**   - Stillbirths - Neonatal deaths - Cord prolapse - Apgar score <7 at 5 minutes - Arterial umbilical cord pH <7.10 - Neonatal intensive care unit admission   **Maternal safety outcomes:**   - Antibiotic administration* - Maternal fever > 38^o^C - Severe postpartum haemorrhage (≥1000mL estimated blood loss) - Maternal admission to the intensive care unit - Other complications requiring hospital assistance^#^   **Maternal experience aspects:**   - Pain during cervical ripening - Overall maternal satisfaction   **POTENTIAL EFFECT MODIFIERS**   - Maternal parity - Maternal age - Maternal BMI - Initial Bishop score - Indication for induction of labour   - Post-dated gestation   - Hypertensive disorders   - Diabetes or gestational diabetes   - Intra-uterine/fetal growth restriction   - Oligohydramnios   - Obstetric cholestasis*   - Advanced maternal age*   - Elective/maternal request*   - Multiple indications* |
| --- |

All intended outcomes are listed in the statistical analysis plan. ^¶^Neonatal pneumonia resulting from aspiration of meconium. ^#^ We intended to examine complications requiring maternal hospital assistance; however, this was not readily available in the trials included. *Insufficient data were available to perform the planned analysis.

Table S2. Search Strategies.

| **Source** | **Search** |
| --- | --- |
| Ovid Medline/ Ovid EMBASE/ Ovid Emcare | Labo?r ADJ2 (induct* OR induce*) \| Bishop* score \|  (Foley* OR Cook OR Atad) ADJ2 (balloon OR catheter).ti,ab,kf. \|  [Cervi* Ripening](https://ovidsp.dc2.ovid.com/ovid-b/ovidweb.cgi?&Controlled+Vocabulary=Mapping%7c6&Return=mapping&S=EEGNFPIOHMEBKKENIPNJHFAFECFCAA00) or Cervi* priming or Pre-induction or Preinduction) \|  (misoprostol or dinoprostone or "prostaglandin E1" or "prostaglandin E2" or "hygroscopic dilator*" or dilapan or dilapan-S or "laminaria tent*" or "osmotic dilator*").ti,ab,kf.\|  Ambulatory Care \| Outpatient* \| [Home Childbirth](https://ovidsp.dc1.ovid.com/ovid-a/ovidweb.cgi?&Controlled+Vocabulary=Mapping%7c2&Return=mapping&S=FFFGFPFEPDACELIOKPNJBHOIJNNOAA00) \| Out of hospital \| Home |
| CINHAL Plus | Labo#r N2 (induct* OR induce*) \|  [Cervi* Ripening](https://ovidsp.dc2.ovid.com/ovid-b/ovidweb.cgi?&Controlled+Vocabulary=Mapping%7c6&Return=mapping&S=EEGNFPIOHMEBKKENIPNJHFAFECFCAA00) \| Cervi* priming Preinduction OR Pre-induction \|  (Foley* OR Cook OR Atad) ADJ2 (balloon OR catheter) \|  (misoprostol or dinoprostone or "prostaglandin E1" or "prostaglandin E2" or "hygroscopic dilator*" or dilapan or dilapan-S or "laminaria tent*" or "osmotic dilator*") \|  Ambulatory Care \| Outpatient* \| [Home Childbirth](https://ovidsp.dc1.ovid.com/ovid-a/ovidweb.cgi?&Controlled+Vocabulary=Mapping%7c2&Return=mapping&S=FFFGFPFEPDACELIOKPNJBHOIJNNOAA00) \| Out of hospital \| Home |
| Cochrane Library  (Trials only) | Labo?r ADJ2 (induct* OR induce*) \| Bishop* score \|  (Foley* OR Cook OR Atad) ADJ2 (balloon OR catheter).ti,ab,kf. \|  [Cervi* Ripening](https://ovidsp.dc2.ovid.com/ovid-b/ovidweb.cgi?&Controlled+Vocabulary=Mapping%7c6&Return=mapping&S=EEGNFPIOHMEBKKENIPNJHFAFECFCAA00) or Cervi* priming or Pre-induction or Preinduction) \|  (misoprostol or dinoprostone or "prostaglandin E1" or "prostaglandin E2" or "hygroscopic dilator*" or dilapan or dilapan-S or "laminaria tent*" or "osmotic dilator*").ti,ab,kf.\|  Ambulatory Care \| Outpatient* \| [Home Childbirth](https://ovidsp.dc1.ovid.com/ovid-a/ovidweb.cgi?&Controlled+Vocabulary=Mapping%7c2&Return=mapping&S=FFFGFPFEPDACELIOKPNJBHOIJNNOAA00) \| Out of hospital \| Home |
| ClinicalTrials.gov | outpatient OR outpatients OR out-patient OR out-patients OR out of hospital OR out-of-hospital OR ambulatory care \| Interventional Studies \| induction of labor OR cervical ripening OR cervical priming OR cervix priming OR cervix ripening OR preinduction OR pre-induction OR (labor NOT work) OR (labour NOT work) |
| WHO ICTRP | Induction AND labour OR labour induction OR Cervical ripening OR Cervical priming Pre-induction OR Preinduction AND out-patient* OR outpatient* OR out of hospital OR out-of-hospital OR ambulatory care |
| Scopus | ( ( TITLE-ABS-KEY ( "Induc*"  W/2  labo?r )  OR  TITLE-ABS-KEY ( bishop*  AND score )  OR  TITLE-ABS-KEY ( pre-induction )  OR  TITLE-ABS-KEY ( preinduction )  OR  TITLE-ABS-KEY ( cervi*  AND ripening )  OR  TITLE-ABS-KEY ( cervi*  AND priming )  OR  TITLE-ABS-KEY ( ( foley*  OR  cook*  OR  atad )  W/2  ( balloon  OR  catheter ) )  OR  TITLE-ABS-KEY ( "Double balloon" )  OR  TITLE-ABS-KEY ( "Single balloon" )  OR  TITLE-ABS-KEY ( "Prostaglandin E2" )  OR  TITLE-ABS-KEY ( dinoprostone )  OR  TITLE-ABS-KEY ( "Prostaglandin E1" )  OR  TITLE-ABS-KEY ( misoprostol )  OR  TITLE-ABS-KEY ( "hygroscopic dilator*" )  OR  TITLE-ABS-KEY ( dilapan* )  OR  TITLE-ABS-KEY ( "laminaria tent*" )  OR  TITLE-ABS-KEY ( "osmotic dilator*" )  OR  TITLE-ABS-KEY ( oxytocin )  OR  TITLE-ABS-KEY ( oxytocics )  AND  TITLE-ABS-KEY ( out-patient* )  OR  TITLE-ABS-KEY ( outpatient* )  OR  TITLE-ABS-KEY ( "out of hospital" )  OR  TITLE-ABS-KEY ( "out-of-hospital" )  OR  TITLE-ABS-KEY ( ambulatory ) ) )  AND  ( ( randomi?ed  AND  controlled  AND  trial* ) )  AND  ( controlled  AND  clinical  AND  trial* ) |

Table S3. Characteristics of trials for which enquiry was sent but did not contribute to IPD meta-analysis (n=8).

| **First author and year** | **Published or conference abstract only** | **Recruitment period** | **Country** | **Method used for outpatient group** | **Method used for inpatient group** | **Other group (if any)** | **N** | **Study population** | **Reason for not contributing IPD** |
| --- | --- | --- | --- | --- | --- | --- | --- | --- | --- |
| *Data not shared: Outpatient balloon catheter versus inpatient balloon catheter comparison* | | | | | | | | | |
| Sciscione 2001 (1) | Published | May  1998 to December 1999 | USA | 16F Foley catheter  Filled with 30-mL sterile water: sent home after a normal CTG | 16F Foley catheter  Filled with 30-mL sterile water: CTG post-insertion | - | 111 | Primip + multip  Low-risk  Gest: ≥ 37.0  ROM: No  BS ≤ 5  AFI > 5^th^ centile  Prev CS: No | Data not available any more |
| Wilkinson 2015 – COPRA (2) | Published | October 2012 to July 2013 | Australia | Cooks^®^ Cervical Ripening (double-balloon) Balloon inflated each  balloon with 70–80 mL of sterile water: sent home after a normal CTG | Cooks^®^ Cervical Ripening (double-balloon) Balloon inflated each  balloon with 70–80 mL of sterile water: 20 min CTG post-insertion | **-** | 48 | Primip + multip  Low-risk  Gest: 37-42  ROM: No  BS ≤ 7  Prev CS: No | Not willing to share |
| Rahman 2020 (3) | Published (pre-print) | August 2017 to May 2018 | Malaysia | 16 F or 18 F latex Foley catheter and the balloon was inflated with 60 mL of distilled water: no post-insertion CTG | 16 F or 18 F latex Foley catheter and the balloon was inflated with 60 mL of distilled water: no post-insertion CTG | - | 60 | Primip + multip  Low-risk  Gest: ≥ 37.0  ROM: No  BS ≤ 6  Prev CS: No | Not willing to share |
| Hamdan 2021 (4) | Published | February 2019 to April 2020 | Malaysia | 18F Foley catheter  Filled with 60 mL sterile water: sent home after a normal CTG | 18F Foley catheter  Filled with 60 mL sterile water: post-insertion CTG | - | 163 | Multip  Low-risk  Gest: ≥ 37.0  ROM: No  BS ≤ 6  Prev CS: No | Not willing to share |
| Crosland (5) | Published as an abstract | 2016 to 2020 | USA | Foley catheter | Foley catheter | - | 81 | Primip + multip  Low-risk  Gest: ≥ 37.0  ROM: NS  BS NS  Prev CS: NS | Not willing to share |
| *Data not shared: Outpatient vaginal dinoprostone versus inpatient vaginal dinoprostone comparison* | | | | | | | | | |
| Greg 1998 (6) | Published as an abstract | NA | Canada | Prostaglandin E2 gel | Prostaglandin E2 gel | - | NA | NA | Data not available any more |
| Biem 2002 (7) | Published | July 1999 and September 2001 | Canada | Controlled-release prostaglandin E2 pessary 10mg: sent home after normal CTG | Controlled-release prostaglandin E2 pessary 10mg | - | 300 | Primip + multip  Low-risk  Gest: ≥ 37.0  ROM: No  BS ≤ 6  Prev CS: No | Unable to trace the authors |
| Wilkinson 2015 – OPRA (8) | Published | August 2008 to May 2011 | Australia | Nulliparous women received 2 mg of Prostaglandin E2, and parous  women 1 mg of Prostaglandin E2: minimum  satisfactory 40-minute electronic fetal monitoring | Nulliparous women received 2 mg of Prostaglandin E2, and parous  women 1 mg of Prostaglandin E2: 20-minute electronic fetal monitoring and monitoring for uterine activity | - | 827 | Primip + multip  Low-risk  Gest: 37-42  ROM: No  BS NS  Prev CS: No | Not willing to share |

*Wing et al 2008 is a three-arm trial providing data for both high-dose and low-dose groups. Nullip=Nulliparous women; Multip=multiparous women; NS: Not stated; BS: Bishop score, ROM: Rupture of membranes; AFI: Amniotic fluid index; CS: Caesarean section.

**References**

1. Sciscione AC, Muench M, Pollock M, Jenkins TM, Tildon-Burton J, Colmorgen GH. Transcervical Foley catheter for preinduction cervical ripening in an outpatient versus inpatient setting. Obstet Gynecol. 2001;98(5 Pt 1):751-6.

2. Wilkinson C, Adelson P, Turnbull D. A comparison of inpatient with outpatient balloon catheter cervical ripening: a pilot randomized controlled trial. BMC Pregnancy Childbirth. 2015;15:126.

3. Rahman RAM, A. Prospective randomized controlled trial comparing inpatient and outpatient Foley catheter cervical ripening (Pre-print). Research Square. 2020.

4. Hamdan M, Shuhaina S, Hong JGS. Outpatient vs inpatient Foley catheter induction of labor in multiparas with unripe cervixes: A randomized trial. 2021;100(11):1977-85.

5. Crosland AS, V. Naidoo, D. Patient satisfaction during outpatient versus inpatient Foley catheter induction of labor. American Journal of Obstetrics & Gynecology. 2022:S680.

6. Greg R. Randomized controlled trial of inpatient vs. outpatient administration of prostaglandin E2, gel for induction of labour at term. American Journal of Obstetrics and Gynecology 1998;178(1 Pt 2):S92.

7. Biem SR, Turnell RW, Olatunbosun O, Tauh M, Biem HJ. A randomized controlled trial of outpatient versus inpatient labour induction with vaginal controlled-release prostaglandin-E2: effectiveness and satisfaction. J Obstet Gynaecol Can. 2003;25(1):23-31.

8. Wilkinson C, Bryce R, Adelson P, Turnbull D. A randomised controlled trial of outpatient compared with inpatient cervical ripening with prostaglandin E₂ (OPRA study). BJOG. 2015;122(1):94-104.

Table S4. Baseline characteristics of participants in the eleven included RCTs.

| **Characteristics** | | | **Outpatient group** | **Inpatient group** | | |
| --- | --- | --- | --- | --- | --- | --- |
| **Henry 2013** | | | | | | |
|  | | | N=50 | N=51 | | |
| Maternal age (years), mean (SD) | | | 32.7 (4.4) | 32.9 (5.1) | | |
| BMI kg/m^2^, mean (SD) | | | 24.1 (4.2) | 23.0 (4.2) | | |
| Gestational age (weeks), median (IQR) | | | 41.2 (0.8) | 41.2 (1.1) | | |
| Ethnicity | | |  |  | | |
| Caucasian | | | 41/50 (82.0) | 41/51 (80.4) | | |
| Hispanic | | | - | - | | |
| Asian | | | 5/50 (10.0) | 9/51 (17.6) | | |
| Black | | | - | - | | |
| Other | | | 4/50 (8.0) | 1/51 (2.0) | | |
| Initial Bishop score*, median (IQR) | | | 2.7 (1.7) | 2.9 (1.6) | | |
| Parity, n/N (%) | | |  |  | | |
| 0 | | | 45/50 (90.0) | 46/51 (90.2) | | |
| >=1 | | | 5/50 (10.0) | 5/51 (9.8) | | |
| Indication for IOL, n/N(%) | | |  |  | | |
| other/unknown | | | 2/50 (4.0) | 4/51 (7.8) | | |
| hypertensive disorders | | | 3/50 (6.0) | 2/51 (3.9) | | |
| post-dated pregnancy | | | 38/50 (76.0) | 35/51 (68.6) | | |
| diabetes/gestational diabetes | | | 3/50 (6.0) | 6/51 (11.8) | | |
| fetal growth restriction | | | 1/50 (2.0) | 3/51 (5.9) | | |
| obstetric cholestasis | | | 3/50 (6.0) | 1/51 (1.9) | | |
| No missing data | | | | | | |
| **Policiano 2016** | | | | | | |
|  | | | N=65 | N=65 | | |
| Maternal age (years)*, mean (SD) | | | 30.6 (6.3) | 31.7 (5.5) | | |
| BMI kg/m^2^, mean (SD) | | | 24.4 (6.2) | 25.7 (6.4) | | |
| Gestational age (weeks), median (IQR) | | | 41.0 (1.0) | 40.0 (3.0) | | |
| Ethnicity | | |  |  | | |
| Caucasian | | | 59/65 (90.8) | 56/65 (86.2) | | |
| Hispanic | | | - | - | | |
| Asian | | | - | - | | |
| Black | | | - | - | | |
| Other | | | 6/65 (9.2) | 9/65 (13.9) | | |
| Initial Bishop score*, median (IQR) | | | 2.0 (2.0) | 2.0 (2.0) | | |
| Parity, n/N (%) | | |  |  | | |
| 0 | | | 50/65 (76.9) | 47/65 (72.3) | | |
| >=1 | | | 15/65 (23.1) | 18/65 (27.7) | | |
| Indication for IOL, n/N(%) | | |  |  | | |
| other/unknown | | | 0 | 4/65 (6.2) | | |
| hypertensive disorders | | | 4/65 (6.2) | 4/65 (6.2) | | |
| post-dated pregnancy | | | 43/65 (66.2) | 35/65 (53.9) | | |
| diabetes/gestational diabetes | | | 8/65 (12.3) | 6/65 (9.2) | | |
| fetal growth restriction | | | 8/65 (12.3) | 9/65 (13.8) | | |
| obstetric cholestasis | | | 2/65 (3.1) | 7/65 (10.8) | | |
| No missing data | | | | | | |
| **Kuper 2018** | | | | | | |
|  | | | N=65 | | N=64 | |
| Maternal age (years), mean (SD) | | | 25.9 (4.2) | | 25.3 (4.2) | |
| BMI, mean (SD) | | | 33.9 (6.9) | | 33.2 (6.4) | |
| Gestational age (weeks), median (IQR) | | | 39.4 (0.4) | | 39.3 (0.4) | |
| Ethnicity | | |  | |  | |
| Caucasian | | | 8/65 (12.3) | | 5/64 (7.8) | |
| Hispanic | | | 1/65 (1.5) | | 0 | |
| Asian | | | - | | - | |
| Black | | | 55/65 (84.6) | | 58/64 (90.6) | |
| Other | | | 1/65 (1.5) | | 1/64 (1.6) | |
| Initial Bishop score, median (IQR) | | | 2.0 (0.5) | | 2.0 (0.7) | |
| Parity, n/N (%) | | |  | |  | |
| 0 | | | 0 | | 0 | |
| >=1 | | | 65 (100) | | 64 (100) | |
| Indication for IOL, n/N (%) | | |  | |  | |
| other/unknown | | | 59/65 (90.7) | | 61/64 (95.3) | |
| hypertensive disorders | | | 0 | | 0 | |
| post-dated pregnancy | | | 4/65 (6.1) | | 1/64 (1.6) | |
| diabetes/gestational diabetes | | | 1/65 (1.5) | | 2/64 (3.1) | |
| fetal growth restriction | | | 0 | | 0 | |
| >one indication | | | 1/65 (1.5) | | 0 | |
| No missing data | | |  | |  | |
| **Beckmann 2019** | | | | | | |
|  | | | N=215 | | N=233 | |
| Maternal age (years), mean (SD) | | | 30.2 (5.3) | | 30.7 (5.1) | |
| BMI, mean (SD) | | | 28.1 (6.3) | | 28.5 (6.6) | |
| Gestational age (weeks), median (IQR) | | | 41.0 (1.1) | | 41.0 (0.9) | |
| Ethnicity | | |  | |  | |
| Caucasian | | | 172/215 (80.0) | | 194/233 (83.3) | |
| Hispanic | | | - | | - | |
| Asian | | | 21/215 (9.8) | | 23/233 (9.9) | |
| Black | | | 2/215 (0.9) | | 1/233 (0.4) | |
| Other | | | 20/215 (9.3) | | 14/233 (6.0) | |
| Initial Bishop score*, median (IQR) | | | 4.0 (2.0) | | 3.0 (2.0) | |
| Parity, n/N (%) | | |  | |  | |
| 0 | | | 157/215 (73.0) | | 155/233 (66.5) | |
| >=1 | | | 58/215 (27.0) | | 78/233 (33.5) | |
| Indication for IOL, n/N (%) | | |  | |  | |
| other/unknown | | | 21/215 (9.8) | | 18/233 (7.7) | |
| hypertensive disorders | | | 0 | | 0 | |
| post-dated pregnancy | | | 152/215 (70.7) | | 172/233 (73.8) | |
| diabetes/gestational diabetes | | | 8/215 (3.7+) | | 10/233 (4.3) | |
| fetal growth restriction | | | 0 | | 0 | |
| advanced maternal age | | | 10/215 (4.6) | | 9/233 (3.9) | |
| elective/social reasons | | | 24/215 (11.2) | | 24/233 (10.3) | |
| **Chen 2019** | | | | | | |
|  | | | N=19 | | N=19 | |
| Maternal age (years), mean (SD) | | | 34.0 (3.6) | | 34.0 (4.2) | |
| BMI*, mean (SD) | | | 24.0 (6.0) | | 24.0 (8.3) | |
| Gestational age (weeks), median (IQR) | | | 41.4 (1.9) | | 41.3 (2.9) | |
| Ethnicity | | |  | |  | |
| Caucasian | | | 15/19 (78.9) | | 15/19 (78.9) | |
| Hispanic | | | - | | - | |
| Asian | | | 1/19 (5.3) | | 3/19 (15.8) | |
| Black | | |  | |  | |
| Other | | |  | |  | |
| Initial Bishop score*, median (IQR) | | | Not reported | | Not reported | |
| Parity, n/N (%) | | |  | |  | |
| 0 | | | 17/19 (89.5) | | 17/19 (89.5) | |
| >=1 | | | 2/19 (10.5) | | 2/19 (10.5) | |
| Indication for IOL, n/N (%)* | | |  | |  | |
| other/unknown | | | 2/19 (10.5) | | 1/19 (5.3) | |
| hypertensive disorders | | | 1/19 (5.3) | | 3/19 (15.8) | |
| post-dated pregnancy | | | 12/19 (63.2) | | 9/19 (47.4) | |
| diabetes/gestational diabetes | | | 2/19 (10.5) | | 2/19 (10.5) | |
| fetal growth restriction | | | 1/9 (5.3) | | 2/19 (10.5) | |
| oligohydramnios | | | 0 | | 1/19 (5.3) | |
| * 2 missing indications | | | | | | |
| **Ausbeck 2020** | | | | | | |
|  | | | N=63 | | N=63 | |
| Maternal age (years), mean (SD) | | | 22.5 (4.4) | | 21.8 (3.6) | |
| BMI, mean (SD) | | | 31.0 (5.4) | | 33.9 (7.7) | |
| Gestational age (weeks), median (IQR) | | | 39.3 (0.3) | | 39.4 (0.3) | |
| Ethnicity | | |  | |  | |
| Caucasian | | | 10/63 (15.9) | | 6/63 (9.5) | |
| Hispanic | | | - | | - | |
| Asian | | | 2/63 (3.2) | | 0 | |
| Black | | | 44/63 (69.8) | | 49/63 (77.8) | |
| Other | | | 6/63 (9.5) | | 6/63 (9.5) | |
| Initial Bishop score*, median (IQR) | | | 1.0 (1.0) | | 1.0 (1.0) | |
| Parity, n/N (%) | | |  | |  | |
| 0 | | | 63/63 (100) | | 63/63 (100) | |
| >=1 | | | 0 | | 0 | |
| Indication for IOL, n/N (%) | | |  | |  | |
| other/unknown | | | 59/63 (93.6) | | 58/63 (92.0) | |
| hypertensive disorders | | | Not reported | | Not reported | |
| post-dated pregnancy | | | 4/63 (6.3) | | 5/63 (7.9) | |
| diabetes/gestational diabetes | | | Not reported | | Not reported | |
| fetal growth restriction | | | Not reported | | Not reported | |
| **Haavisto 2020** | | | | | | |
|  | | | N=57 | | N=56 | |
| Maternal age (years), mean (SD) | | | 30.0 (4.2) | | 30.4 (4.4) | |
| BMI*, mean (SD) | | | 25.8 (4.7) | | 26.0 (3.9) | |
| Gestational age (weeks), median (IQR) | | | 41.7 (0.3) | | 41.6 (1.1) | |
| Ethnicity | | | Not reported | | Not reported | |
| Initial Bishop score*, median (IQR) | | | 4.0 (2.0) | | 4.0 (2.0) | |
| Parity, n/N (%) | | |  | |  | |
| 0 | | | 36/57 (63.2) | | 35/56 (62.5) | |
| >=1 | | | 21/57 (36.8) | | 21/56 (37.5) | |
| Indication for IOL, n/N (%) | | | Not reported | | Not reported | |
| **Kohari 2021** | | | | | | |
|  | | | N=29 | | N=24 | |
| Maternal age (years), mean (SD) | | | 31.4 (5.3) | | 33.3 (6.5) | |
| BMI*, mean (SD) | | | 25.7 (11.6) | | 25.0 (10.3) | |
| Gestational age (weeks), median (IQR) | | | 39.9 (1.6) | | 40.4 (1.8) | |
| Ethnicity | | |  | |  | |
| Caucasian | | | 15/29 (51.7) | | 17/24 (70.8) | |
| Hispanic | | | 5/29 (17.2) | | 2/24 (8.3) | |
| Asian | | | 5/29 (17.2) | | 0 | |
| Black | | | 2/29 (6.9) | | 3/24 (12.5) | |
| Other | | | 2/29 (6.9) | | 2/24 (8.3) | |
| Initial Bishop score*, median (IQR) | | | 2.0 (1.0) | | 1.5 (1.0) | |
| Parity, n/N (%) | | |  | |  | |
| 0 | | | 24/29 (82.8) | | 20/24 (83.3) | |
| >=1 | | | 5/29 (17.2) | | 4/24 (16.7) | |
| Indication for IOL, n/N (%) | | | Not reported | | Not reported | |
| **Pierce-Williams 2022** | | | | | | |
|  | | N=15 | | | N=15 | |
| Maternal age (years), mean (SD) | | 27.2 (6.2) | | | 25.7 (4.7) | |
| BMI*, mean (SD) | | 36.4 (6.3) | | | 34.5 (7.0) | |
| Gestational age (weeks), median (IQR) | | 39.3 (0.6) | | | 39.1 (1.0) | |
| Ethnicity | |  | | |  | |
| Caucasian | | 4/15 (26.7) | | | 3/15 (20.0) | |
| Hispanic | | 1/15 (6.7) | | | 0 | |
| Asian | | 0 | | | 1/15 (6.7) | |
| Black | | 10/15 (66.7) | | | 11/15 (73.3) | |
| Other | | - | | | - | |
| Initial Bishop score*, median (IQR) | | 3.0 (3.0) | | | 4.0 (2.0) | |
| Parity, n/N (%) | |  | | |  | |
| 0 | | 7/15 (46.7) | | | 8/15 (53.3) | |
| >=1 | | 8/15 (53.3) | | | 7/15 (46.7) | |
| Indication for IOL, n/N (%) | |  | | |  | |
| other/unknown | | 1/15 (6.7) | | | 1/15 (6.7) | |
| hypertensive disorders | | 2/15 (13.3) | | | 0 | |
| post-dated pregnancy | | 2/15 (13.3) | | | 0 | |
| diabetes/gestational diabetes | | 1/15 (6.7) | | | 1/15 (6.7) | |
| fetal growth restriction | | 0 | | | 0 | |
| obstetric cholestasis | | 1/15 (6.7) | | | 0 | |
| elective/social reasons | | 8/15 (53.3) | | | 13/15 (86.7) | |
| **Saad 2022** | | | | | | |
|  | | | N=167 | N=171 | | |
| Maternal age (years), mean (SD) | | | 25.2 (5.6) | 25.9 (5.6) | | |
| BMI*, mean (SD) | | | 33.3 (6.3) | 33.5 (6.7) | | |
| Gestational age (weeks), median (IQR) | | | 39.0 (0.4) | 39.0 (0.3) | | |
| Ethnicity | | |  |  | | |
| Caucasian | | | 38/167 (22.7) | 26/171 (15.2) | | |
| Hispanic | | | 112/167 (67.0) | 120/171 (70.2) | | |
| Asian | | | - | - | | |
| Black | | | 15/167 (8.9) | 21/171 (12.3) | | |
| Other | | | 2/167 (1.2) | 4/171 (2.3) | | |
| Initial Bishop score*, median (IQR) | | | 3.0 (3.0) | 2.0 (2.0) | | |
| Parity, n/N (%) | | |  |  | | |
| 0 | | | 83/167 (49.7) | 78/171 (45.6) | | |
| >=1 | | | 84/167 (50.3) | 93/171 (54.4) | | |
| Indication for IOL, n/N (%) | | |  |  | | |
| other/unknown | | | 5/167 (3.0) | 3/171 (1.7) | | |
| hypertensive disorders | | | 0 | 0 | | |
| post-dated pregnancy | | | 0 | 0 | | |
| diabetes/gestational diabetes | | | 0 | 1/171 (0.6) | | |
| fetal growth restriction | | | 0 | 0 | | |
| obstetric cholestasis | | | 1/167 (0.6) | 0 | | |
| elective/social reasons | | | 161/167 (96.4) | 167/171 (97.6) | | |
| **Wise 2023** | | | | | | |
|  | N=539 | | | | | N=548 |
| Maternal age (years), mean (SD) | 32.3 (5.5) | | | | | 32.3 (5.0) |
| BMI*, mean (SD) | 27.4 (6.6) | | | | | 27.6 (6.7) |
| Gestational age (weeks), median (IQR) | 40.0 (2.2) | | | | | 40.0 (2.1) |
| Ethnicity |  | | | | |  |
| Caucasian | 315/539 (58.4) | | | | | 350/548 (63.9) |
| Hispanic | - | | | | | - |
| Asian | 109/539 (20.2) | | | | | 92/548 (16.8) |
| Black | - | | | | | - |
| Other | 115/539 (21.3) | | | | | 106/548 (19.3) |
| Initial Bishop score*, median (IQR) | Not reported | | | | | Not reported |
| Parity, n/N (%) |  | | | | |  |
| 0 | 420/539 (77.9) | | | | | 417/548 (76.1) |
| >=1 | 119/539 (22.1) | | | | | 131/548 (23.9) |
| Indication for IOL, n/N (%) |  | | | | |  |
| other/unknown | 64/539 (11.9) | | | | | 68/548 (12.4) |
| hypertensive disorders | 41/539 (7.6) | | | | | 36/548 (6.6) |
| post-dated pregnancy | 171/539 (31.7) | | | | | 177/548 (32.3) |
| diabetes/gestational diabetes | 146/539 (27.0) | | | | | 136/548 (24.8) |
| fetal growth restriction | 51/539 (9.5) | | | | | 54/548 (9.8) |
| oligohydramnios | 3/539 (0.6) | | | | | 5/548 (0.9) |
| advanced maternal age | 34/539 (6.3) | | | | | 28/548 (5.1) |
| obstetric cholestasis | 12/539 (2.2) | | | | | 11/548 (2.0) |
| elective/social reasons | 17/539 (3.1) | | | | | 33/548 (6.0) |

IOL: Induction of labour; BMI: Body mass index; IUGR: Intrauterine growth restriction; IQR: Inter-quartile range; SD: Standard deviation; RCT: Randomized controlled trials.

.

Table S5: Outpatient balloon catheter compared to an inpatient balloon catheter for cervical ripening and induction of labour for secondary outcomes. Both balloon catheters alone in both groups and the RCTs with the use of concurrent oxytocin in the inpatient group.

| **Secondary outcome** | **No. of trials** | **No. of women** | **Crude incidence (%) -**  **Outpatient versus Inpatient methods** | **aOR (95% CI)** | **I^2^ (95% CI)** | **Analysis method*** | **Certainty of the evidence**  **(GRADE)** |
| --- | --- | --- | --- | --- | --- | --- | --- |
| **Delivery outcomes** | | | | | | | |
| Caesarean delivery | 7 | 616 | 20.5 vs 23.4 | 0.82 (0.48;1.39) | 6 (0.0;72.6) | two-stage | ⊕⊕⊕⊝‡  Moderate |
| Caesarean delivery for failure to progress | 7 | 610 | 10.8 vs 12.9 | 0.82 (0.49;1.37) |  | one-stage | ⊕⊕⊕⊝‡  Moderate |
| Caesarean delivery for fetal distress | 7 | 610 | 7.2 vs 7.3 | 0.98 (0.52;1.85) |  | one-stage | ⊕⊕⊕⊝‡  Moderate |
| Unassisted vaginal birth | 7 | 616 | 63.1 vs 59.9 | 1.20 (0.76;1.89) | 0 (0.0;70.8) | two-stage | ⊕⊕⊕⊝‡  Moderate |
| Instrumental vaginal birth | 7 | 616 | 14.7 vs 16.4 | 0.88 (0.55;1.41) |  | one-stage | ⊕⊕⊕⊝‡  Moderate |
| Instrumental vaginal birth for failure to progress in the second stage | 7 | 501 | 8.7 vs 6.0 | 1.70 (0.80;3.61) |  | one-stage | ⊕⊕⊝⊝#‡  Low |
| Instrumental vaginal birth for fetal distress | 7 | 501 | 3.6 vs 7.2 | 0.46 (0.20;1.08) |  | one-stage | ⊕⊕⊕⊝‡  Moderate |
| Time to vaginal birth (hours) | 6 | 478 | 30.9 vs 25.9^¥^ | sHR 0.63 (0.23;1.74) | 91 (84;95) | two-stage | ⊕⊕⊝⊝†‡  Low |
| Total maternal hospital stay (hours) | - | - | - | Insufficient data | - | - | - |
| Total neonatal hospital stay (hours) | - | - | - | Insufficient data | - | - | - |
| **Labour progression outcomes** | | | | | | | |
| Uterine tachysystole | - | - | - | Insufficient data | - | - | - |
| Uterine hyperstimulation | - | - | - | Insufficient data | - | - | - |
| Oxytocin augmentation | 7 | 613 | 75.6 vs 75.7 | 0.98 (0.60;1.59) | - | one-stage | ⊕⊕⊕⊝‡  Moderate |
| Meconium-stained amniotic fluid | 7 | 346 | 14.8 vs 18.8 | 0.74 (0.42;1.32) | - | one-stage | ⊕⊕⊕⊝‡  Moderate |
| Use of epidural analgesia | 5 | 448 | 84.2 vs 88.2 | 0.67 (0.48;0.93) | 0 (0;79) | two-stage | ⊕⊕⊕⊝‡  Moderate |
| Use of more methods for cervical ripening (second methods) | 2 | 183 | 52.1 vs 49.4 | 1.15 (0.86;1.54) | 0 | two-stage | ⊕⊝⊝⊝^#^†‡  Low |
| **Maternal safety outcomes** | | | | | | | |
| Maternal infection | 7 | 618 | 9.9 vs 6.9 | 1.52 (0.84;2.76) | - | one-stage | ⊕⊕⊝⊝  Low^#^ ^‡^ |
| Severe postpartum haemorrhage | 6 | 498 | 1.6 vs 3.6 | 0.35 (0.07;1.69) | - | one-stage | ⊕⊕⊝⊝  Low^#^ ^‡^ |
| Maternal ICU admission | - | - | - | Insufficient data | - | - | - |
| **Neonatal safety outcomes** | | | | | | | |
| Stillbirths | - | - | No stillbirths were reported in either group | - | - | - | - |
| Neonatal deaths | - | - | No neonatal deaths were reported in either group | - | - | - | - |
| Cord prolapse | 3 | 385 | 0.52 vs 1.04 | 0.32 (0.02;4.30) | - | one-stage | ⊕⊕⊝⊝  Low^#^ ^‡^ |
| Apgar score < 7 at 5 mins | 7 | 602 | 1.0 vs 1.7 | 0.60 (0.14;2.55) | - | one-stage | ⊕⊕⊝⊝  Low^#^ ^‡^ |
| Arterial umbilical cord pH < 7.10 | 3 | 326 | 5.9 vs 5.2 | 1.10 (0.43;2.81) | - | one-stage | ⊕⊕⊝⊝  Low^#^ ^‡^ |
| NICU admission | 6 | 471 | 5.9 vs 6.4 | 0.89 (0.42;1.88) | - | one-stage | ⊕⊕⊝⊝  Low^#^ ^‡^ |
| **Pain and satisfaction** | | | | | | | |
| Pain during cervical ripening | - | - | - | Insufficient data | - | - | - |
| Overall satisfaction score | - | - | - | Insufficient data | - | - | - |
| aOR: adjusted odds ratio; sHR: sub-distribution hazard ratio; ICU: intensive care unit; NICU: neonatal intensive care unit. ^¥^Median values reported.  *Two-stage as the primary strategy, one-stage used when zero events are encountered in any arm of any included study. All analyses adjusted for maternal age and parity.  # Downgraded one level for imprecision  † Downgraded one level for inconsistency  ‡ Downgraded one level for concerns on data completeness | | | | | | | |

Table S6. Treatment-covariate interactions for vaginal birth in outpatient versus inpatient IOL.

| **Effect modifier** | **No. of trials** | **No. of women** | **aOR (95% CI) with interaction term, p-value** | **aOR (95% CI) without interaction term, p-value** | **Ratio of two odds ratios^¥^ (95% CI), p-value** | **Likelihood ratio test p-value^¶^** |
| --- | --- | --- | --- | --- | --- | --- |
| Parity | 5 | 2,116 | 0.78 (0.39;1.56), 0.37 | 0.94 (0.56;1.58), 0.77 | 0.80 (0.45;1.42), 0.35 | 0.31 |
| Maternal age | 11 | 2,593 | 0.99 (0.96; 1.02), 0.33 | 0.95 (0.70;1.30), 0.74 | 1.01 (0.72;1.42), 0.94 | 0.37 |
| BMI | 11 | 2,593 | 0.97 (0.94;1.00), 0.09 | 0.91 (0.69;1.22), 0.50 | 1.07 (0.81;1.40), 0.61 | 0.05 |
| Initial Bishop score | 9 | 1,468 | 0.97 (0.83;1.13), 0.63 | 1.02 (0.70;1.50), 0.90 | 0.92 (0.64;1.32), 0.61 | 0.43 |
| Gestational age | 10 | 2,563 | 1.09 (0.91;1.30), 0.33 | 0.98 (0.73;1.32), 0.89 | 1.01 (0.58;1.77), 0.95 | 0.63 |
| Indication for IOL*^#^ |  |  |  |  |  |  |
| - Hypertensive disorders | 4 | 1,371 | 1.12 (0.54;2.29), 0.69 | 0.89 (0.68;1.18), 0.32 | 1.24 (0.65;2.35), 0.40 | 0.73 |
| - Diabetes/ gestational diabetes | 4 | 1,766 | 0.89 (0.70;1.12), 0.26 | 0.82 (0.65;1.03), 0.07 | 1.10 (0.92;1.30), 0.23 | 0.57 |
| *All compared to induction of labour for post-dates (reference) | | | |  |  |  |

aOR: adjusted odds ratio; 95% CI: 95% Confidence Interval; IOL: Induction of labour; BMI: Body mass index.

^#^Data were insufficient for fetal growth restriction, oligohydramnios, obstetric cholestasis, advanced maternal age, elective/maternal request and multiple indications; ^¥^With versus without interaction term; ^¶^Stouffer’s Z-Score Method.

Table S7. Strata-specific treatment effect of outpatient versus inpatient IOL for vaginal birth.

| **Effect modifier** | **Stratum** | **No. of trials** | **No. of women** | **aOR (95% CI), p-value:** |
| --- | --- | --- | --- | --- |
| Maternal age | <35 years | 11 | 2,009 | 0.96 (0.69; 1.34), 0.79 |
|  | ≥35 years | 11 | 584 | 0.87 (0.63; 1.19), 0.33 |
| BMI | <30 kg/m^2^ | 11 | 1,634 | 0.94 (0.66; 1.33), 0.68 |
|  | ≥30 kg/m^2^ | 11 | 959 | 0.85 (0.54; 1.34), 0.43 |
| Gestational age | <40 weeks | 10 | 1,257 | 1.05 (0.60; 1.86), 0.84 |
|  | ≥40 weeks | 10 | 1,336 | 0.89 (0.74; 1.08), 0.20 |

aOR: adjusted odds ratio; 95% CI: 95% Confidence Interval; IOL: Induction of labour;

BMI: Body mass index

Table S8. Two-stage and one-stage (sensitivity analysis) analyses comparing outpatient versus inpatient methods for induction of labour.

| **Primary outcome** | **No. of trials** | **No. of women** | **Two-stage method*** | | **One-stage method^¶^** | **Certainty of the evidence (GRADE)** |
| --- | --- | --- | --- | --- | --- | --- |
|  |  |  | **aOR (95% CI)** | **I^2^ (95% CI)** | **aOR (95% CI)** |  |
| Vaginal delivery | 11 | 2,584 | 0.95 (0.70;1.30) | 33 (0;67) | 0.95 (0.73;1.22) | ⊕⊕⊕⊝†  Moderate |
| Composite maternal outcome | 10 | 2,470 | 0.89 (0.65;1.20) | 6 (0;65) | 0.90 (0.70;1.16) | ⊕⊕⊕⊝‡  Moderate |
| Composite perinatal outcome | 9 | 2,581 | 0.93 (0.75;1.16) | 0 (0;65) | 0.95 (0.74;1.22) | ⊕⊕⊕⊝‡  Moderate |
| aOR: adjusted odds ratio; 95% CI: 95% Confidence Interval. *Random-effects model adjusted for age and parity, Restricted maximum likelihood (REML) estimator with Hartung-Knapp-Sidik-Jonkman variance correction (untruncated)  ¶ A stratified intercept by study and a random treatment effect, covariates age and parity as a fixed effect, maximum likelihood (ML) estimator  † Downgraded one level for inconsistency  ‡ Downgraded one level for concerns about data completeness | | | | | | |

Table S9. As treated analyses comparing vaginal misoprostol versus vaginal dinoprostone for labour induction.

| **Primary outcome** | **No. of trials** | **No. of women** | **aOR (95% CI)** | **I^2^ (95% CI)** |
| --- | --- | --- | --- | --- |
| Vaginal delivery | 7 | 2,159 | 0.85 (0.67;1.07) | 0 (0;71) |
| Composite maternal outcome | 6 | 2,046 | 0.93 (0.66;1.31) | 9 (0;77) |
| Composite perinatal outcome | 7 | 2,159 | 0.86 (0.59;1.24) | 12 (0;74) |

aOR: adjusted odds ratio; 95% CI: 95% Confidence Interval.
